# Palladium decorated on a new dendritic complex with nitrogen ligation grafted to graphene oxide: fabrication, characterization, and catalytic application†

**DOI:** 10.1039/c9ra04511b

**Published:** 2019-09-02

**Authors:** Mohsen Golestanzadeh, Hossein Naeimi

**Affiliations:** Departetment of Organic Chemistry, Faculty of Chemistry, University of Kashan Kashan 8731751167 Iran naeimi@kashanu.ac.ir golestanzadeh@grad.kashanu.ac.ir +98-31-55912397 +98-31-55912388; Child Growth and Development Research Center, Research Institute for Primordial Prevention of Non-Communicable Disease, Isfahan University of Medical Sciences Isfahan 8174673461 Iran

## Abstract

Immobilized Pd nanoparticles on a new ligand, namely, tris(pentaethylene-pentamine)triazine supported on graphene oxide (Pd_np_-TPEPTA_(L)_-GO) was introduced as a novel and robust heterogeneous catalyst for use in C–C bond formation reaction. The Pd_np_-TPEPTA_(L)_-GO catalyst was synthesized by complexation of Pd with TPEPTA as a ligand with high N-ligation sites that were supported on graphene oxide through 3-chloropropyltrimethoxysilane. The prepared catalyst was characterized using some microscopic and spectroscopic techniques. The TPEPTA_(L)_-GO substrate is a 2D heterogeneous catalyst with a high specific surface area and a large amount of N-ligation sites. The Pd_np_-TPEPTA_(L)_-GO catalyst used in the C–C bond formation reaction between aryl or heteroaryl and phenylboronic acid derivatives was applied towards the synthesis of biaryl units in high isolated yields. Notably, a series of competing experiments were performed to establish the selectivity trends of the presented method. Also, this catalyst system was reusable at least six times without a significant decrease in its catalytic activity.

## Abstract 2

Immobilized Pd nanoparticles on a new ligand, namely, tris(pentaethylene-pentamine)triazine supported on graphene oxide (Pd_np_-TPEPTA_(L)_-GO) was introduced as a novel and robust heterogeneous catalyst for use in C–C bond formation reaction.

## Graphical abstract

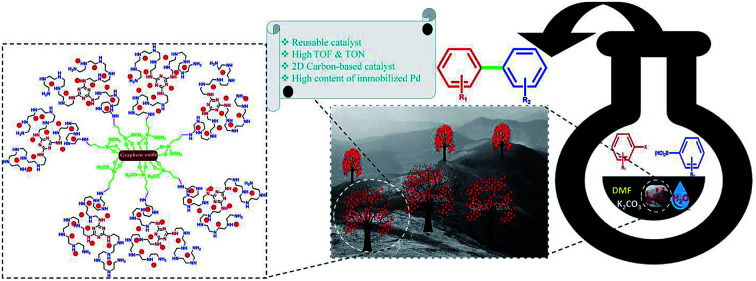

## Introduction

**Scheme 1:**
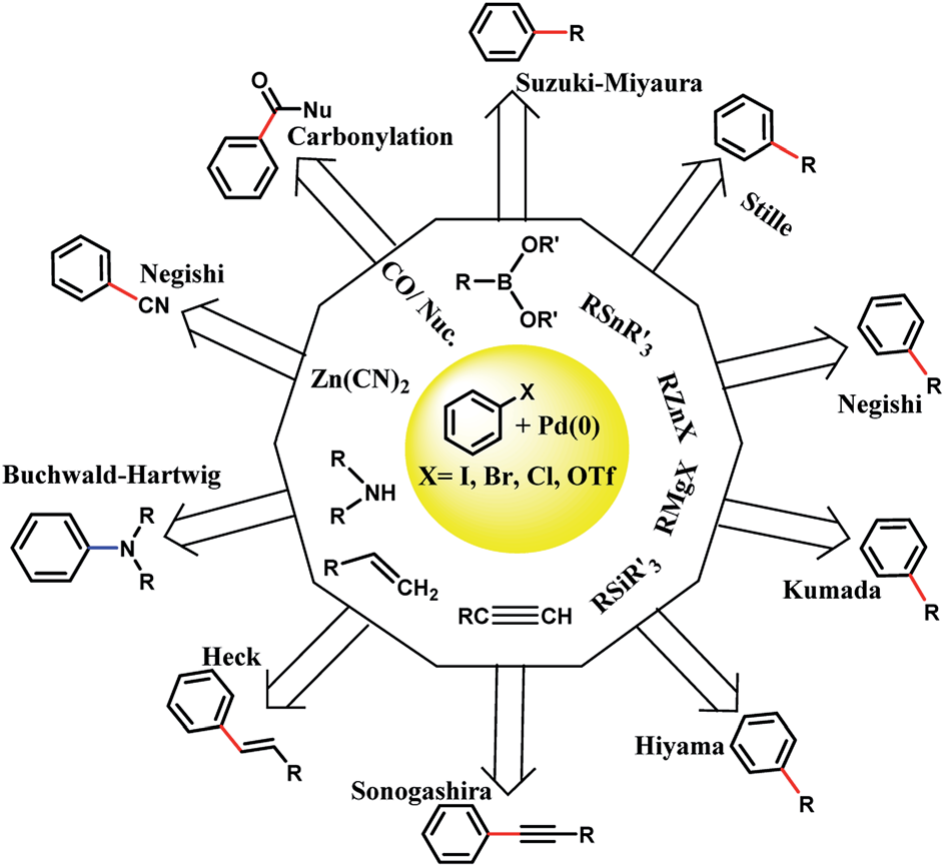
The cross-coupling organic reactions catalyzed by Pd(0). Palladium(0) catalysis has gained widespread use in industrial and academic synthetic chemistry laboratories as a powerful methodology for the formation of carbon–carbon and carbon–heteroatom bonds. R: usually sp^2^ hybridized carbon; the nature of R′ and *M* is dependent on the specific coupling being performed. The new carbon–carbon bond is shown in red color and the new carbon–nitrogen bond is shown in blue color.

In 1803, palladium was discovered by Wollaston in his investigation on refining platinum. Palladium has the ground state electronic configuration of [Kr] 4d^10^ and belongs to the fifth period and 10th group of the periodic table. Palladium has different oxidation states including 0, +1, +2 (the most stable for Pd), and +4. Also, the other unstable oxidation states of palladium are +3, +5, and +6.^1–3^ Most of the Pd(0) complexes are simply oxidized in air and therefore, the development of new Pd(0) complexes is still appealing. Importantly, the heterogeneous stabilized Pd(0) in less conventional ways should be cited. The ability of palladium(0) to construct new bonds between carbon–carbon and carbon–heteroatom is important in organic synthesis (Scheme 1).

**Fig. 1:**
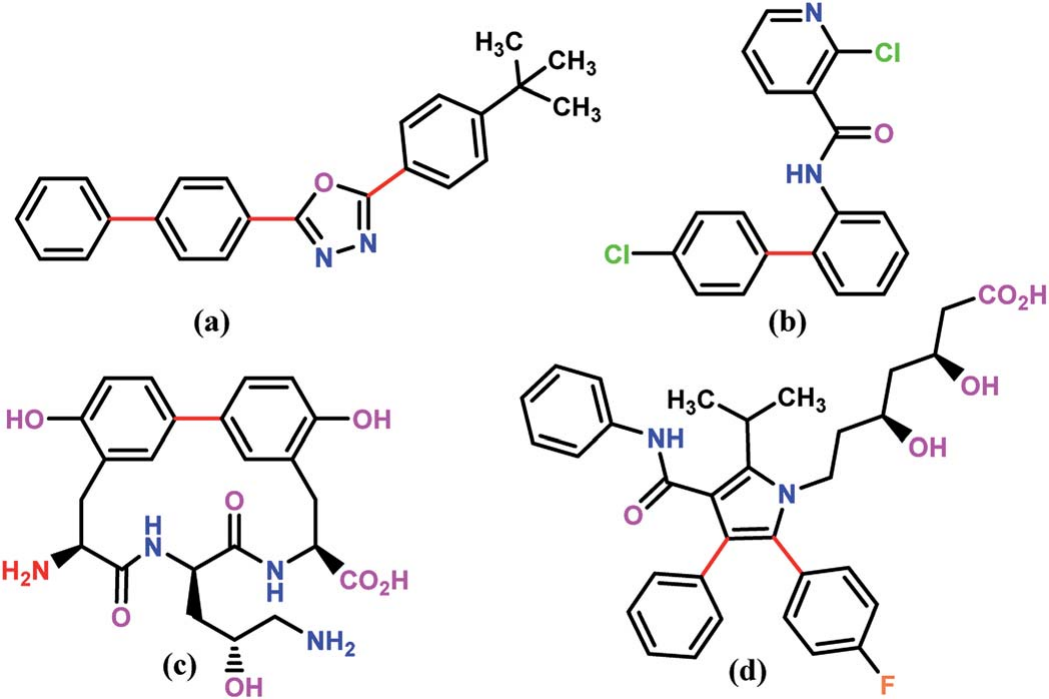
The chemical structure of selected industrially important compounds containing bi(hetero)aryl moiety where Suzuki–Miyaura coupling is used to form the key carbon–carbon bonds (shown in red); (a) is a component of OLEDs (organic light-emitting diodes) or electron conducting material (PBD). (b) is an agrochemical that is most commonly used as a fungicide (Boscalid). (c) is an antibiotic (Biphenomycin B). (d) is a pharmaceutical compound that lowers blood cholesterol (Lipitor).

For example, the Suzuki–Miyaura cross coupling reaction is a highly efficient and robust organic reaction that includes cross coupling between a sp^2^-hybridized halide and sp^2^-hybridized boronic acid to form a Csp^2^–Csp^2^ bond. The biaryl or biheteroaryl motifs are ubiquitous among the wide range of industrially and pharmaceutically important compounds (Fig. 1).

Generally, the homogeneous catalysts of Pd complexes catalyze the cross coupling Suzuki–Miyaura reaction.^4–6^ However, using homogeneous Pd complexes has several disadvantages including low efficiency in the separation of the catalyst and high cost of the Pd element at the industrial scale. Therefore, the immobilization of Pd complexes or Pd nanoparticles on different organic, inorganic, and hybrid supports has been made to provide heterogeneous Pd catalysts. Unfortunately, the supported Pd complex catalysts often suffer from low specific surface area (SSA), space confinement in mesoporous supports, less reactivity of support, reduced availability of the metal complex, and low thermal and chemical stability with respect to leaching of metals.^7–9^

**Fig. 2:**
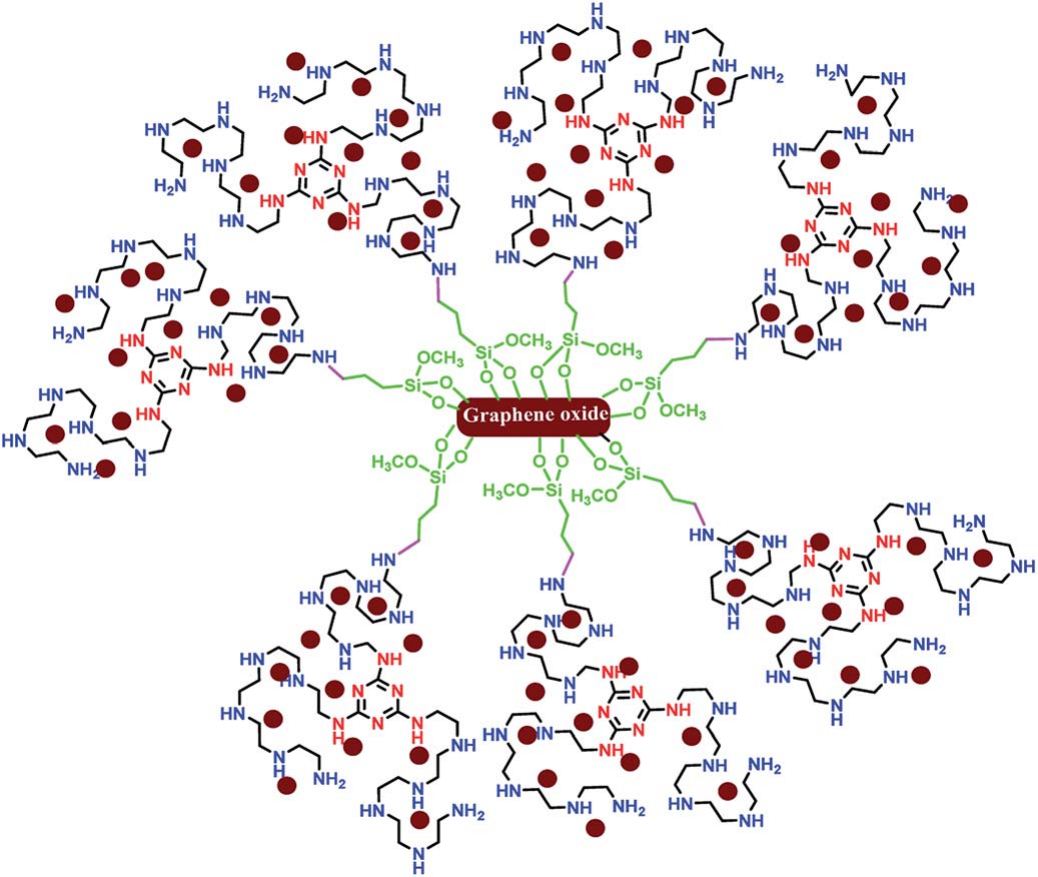
A representation of the chemical structure of Pd_np_-TPEPTA_(L)_-GO. The nitrogen atoms of triazine are shown in red color and the nitrogen atoms in pentaethylenepentamine are shown in blue color. The new bond between the nitrogen atom of the ligand and the carbon atom of propyl trimethoxysilane supported on graphene oxide is shown in pink color.

Recently, two-dimensional supports such as graphene,^10^ graphene oxide (GO),^11^ and reduced graphene oxide (RGO)^12^ have attracted extensive attention in heterogeneous catalysis due to their high SSA,^13^ more surface active sites,^14^ excellent photocatalyst support,^15^ superior electron mobility,^16^ the availability of functional groups from all side of the sheets,^17,18^ and other excellent properties.^19,20^ As examples of the utilization of two-dimensional graphene in coupling reactions, we have considered previously reported papers. Very recently, Huang and co-workers reported a robust 3D ionic liquid supported on RGO/Pd nanocomposites for the Suzuki cross coupling reaction.^21^ Sengupta *et al.* reported an efficient aminobis(phosphine)-Pd^II^ complex on GO in different coupling reactions.^22^ Also, the effect of GO support on the catalytic performance of Pd–Fe_3_O_4_/GO, Pd–Co_3_O_4_/GO and Pd–Ni(OH)_2_/GO in the Suzuki cross coupling reaction was considered by Elazab and co-workers.^23,24^ In accordance with the various, constantly evolving fields, the development of efficient two-dimensional graphene/Pd nanocomposites is of enormous importance.^25–43^ In addition, Li *et al.* reported a new catalyst (palladium decorated on nitrogen-doped graphene nanoshells) for *N*-allylation reaction.^44^ Herein, it is notable that most of the reported catalysts applied in coupling reactions were unstable in air, less reusable or totally non-reusable, and had low loading of active sites. Undoubtedly, the design, preparation, and application of superior heterogeneous catalysts with high SSA and more reactive sites as well as highly coordinating donor atoms for the stabilization of Pd(0) in cross coupling reactions is still appealing. In continuation of our previous studies on two-dimensional heterogeneous catalysts,^45–48^ this research highlights the application of graphene oxide modified with a novel ligand with high N-ligation sites for the stabilization of palladium nanoparticles with oxidation state (0) in the Suzuki–Miyaura cross coupling reaction. The schematic representation of Pd_np_-TPEPTA_(L)_-GO is shown in Fig. 2.

## Result and discussion

### Catalyst synthesis and its characterization

**Scheme 2:**
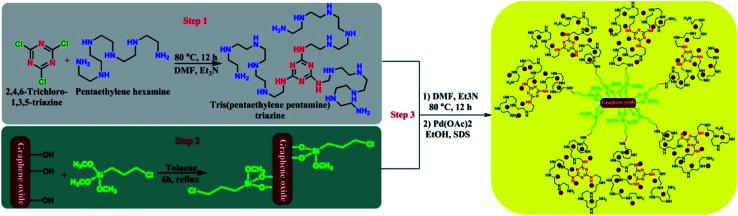
A synthetic pathway for the synthesis of Pd_np_-TPEPTA_(L)_-GO.

The supported palladium nanoparticles on graphene oxide including tris-pentaethylenepentaminetriazine as a novel ligand (Pd_np_-TPEPTA_(L)_-GO) were prepared according to the following synthetic route, as shown in Scheme 2, through three steps. First, the preliminary ligand was prepared from the reaction of 2,4,6-trichloro-1,3,5-triazine (TCT) and pentaethylenehexamine (PEHA). We checked the reaction of TCT and PEHA under different conditions to obtain a high yield of tris(pentaethylene-pentamine)triazine (TPEPTA).^49–53^ This step has a vital role in the preparation of TPEPTA with high N-ligation sites because a decrease in the substitution of PEHA with chlorine atom in TCT and the total yield of TPEPTA have a significant effect on the highimmobilization of palladium nanoparticles. To obtain the optimized reaction conditions for this step, the reaction of TCT and PEHA was investigated under different circumstances and the obtained results are shown in Table 1S (see ESI†). In addition, TPEPTA was analyzed using a CHNS analyzer and the obtained results confirmed the successful substitution of three equivalents of PEHA on TCT substrate (see ESI†).

In the second step of the preparation of the Pd_np_-TPEPTA_(L)_-GO catalyst, 3-chloropropyltrimethoxysilane was anchored on graphene oxide, according to the previous literature with some modification.^54,55^ Similar to the first step, the second step is also very important because a large amount of 3-chloropropyl on GO results in the stabilization of the highest amount of ligand on the surface of GO. We determined the amount of chloropropylsilyl groups on the surface of GO using Mohr's method (see ESI†).^56,57^ The total density of chloropropylsilyl groups on GO was estimated to be about 0.95 mmol g^−1^. The chloropropylsilyl groups not only cover the GO sheets but also make them ready for further surface functionalization.^54^ Finally, in the third step, through two sequential steps, the stabilization of TPEPTA on the surface of GO was performed at 80 °C for 12 h. Then, TPEPTA_(L)_-GO was reacted with Pd(OAc)_2_, leading to the immobilization of palladium nanoparticles in the presence of sodium dodecyl sulfate (SDS) as the surfactant for controlling the size of palladium particles^58,59^ in order to produce Pd_np_-TPEPTA_(L)_-GO catalyst.

To confirm the functionalization of GO, the Pd_np_-TPEPTA_(L)_-GO catalyst was characterized by the following techniques: Scanning Electron Microscopy (SEM), Energy Dispersive X-ray spectroscopy (EDX), Transmission Electron Microscopy (TEM), Fourier Transform Infrared Spectroscopy (FT-IR), Powder X-ray Diffraction (XRD), and Thermogravimetric Analysis (TGA).

**Fig. 3:**
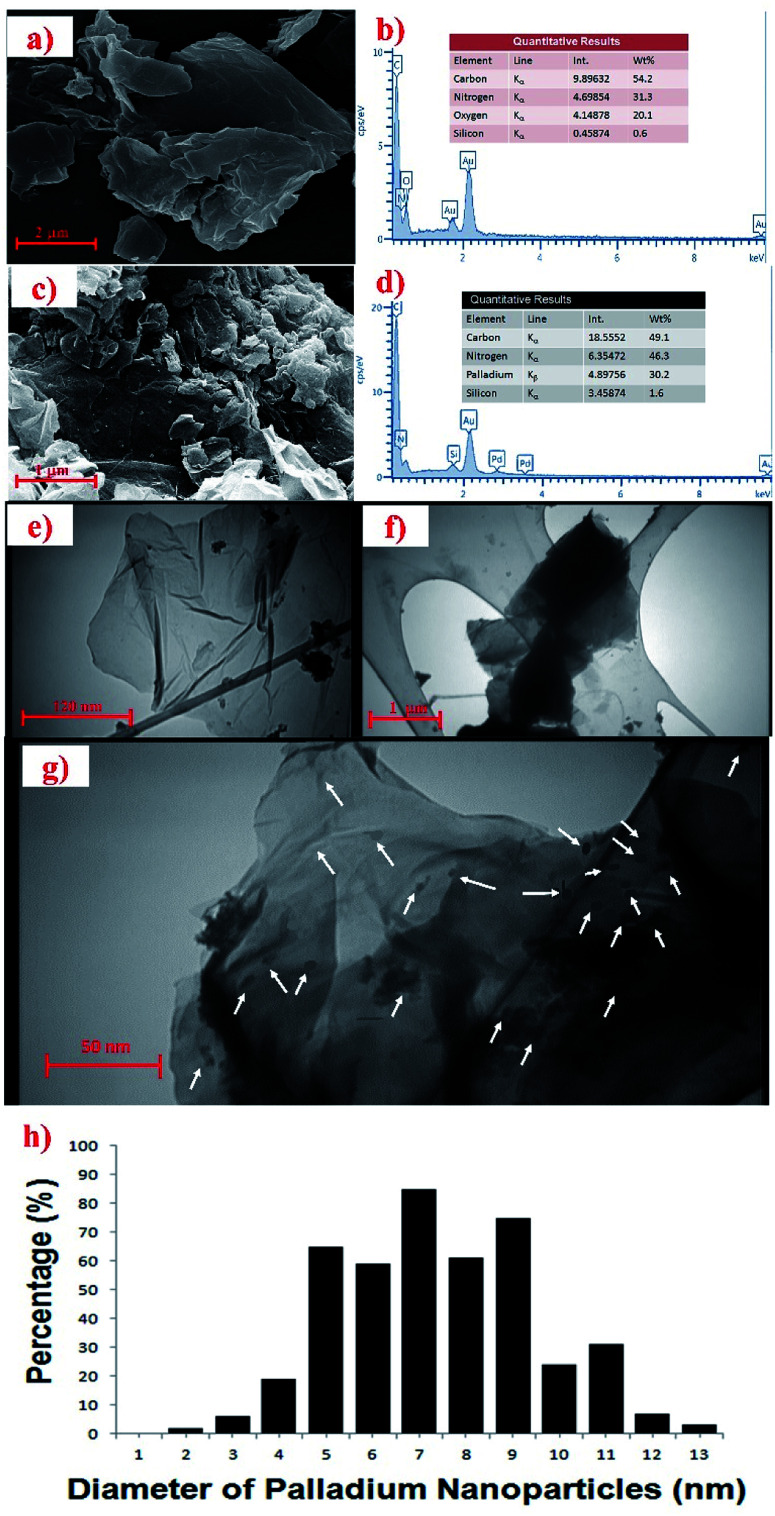
a) FE-SEM of GO [details of imaging: scale bar 2 μm, SEM HV: 15.00 kV, WD: 6.060 mm, view field: 8.668 μm, SEM magnification: 25.00k×]. (b) The EDX analysis of synthesized GO. (c) FE-SEM of Pd_np_-TPEPTA_(L)_-GO [details of imaging: scale bar 1 μm, EHT: 15.00 kV, WD: 8.9 mm, signal A: SE2, SEM magnification: 10.000k×]. (d) The EDX analysis of Pd_np_-TPEPTA_(L)_-GO catalyst. (e), (f), and (g) TEM images of the Pd_np_-TPEPTA_(L)_-GO catalyst with different scale bars (1 μm, 120 nm, and 50 nm) and situations [details of imaging: 80 kV, limit of detection of 2 nm or less] (the white arrows on (g) show the palladium nanoparticles). (h) Particle size distribution results for the Pd_np_-TPEPTA_(L)_-GO catalyst.

In order to study the surface morphologies of GO and the Pd_np_-TPEPTA_(L)_-GO catalyst, SEM images were recorded. As shown in Fig. 3a, the plate-like forms including the flaky layered texture of GO was observed. The EDX analysis of GO is shown in Fig. 3b. The results of EDX analysis show the presence of carbon, nitrogen, and oxygen. The absence of metal elements such as potassium and sodium clearly showed that the washing step was successful. The surface morphology of the Pd_np_-TPEPTA_(L)_-GO catalyst was also studied by SEM (Fig. 3c). As shown in this figure, the sheets of GO were covered by the foreign matter Pd_np_-TPEPTA_(L)_. In this image, both the plate-like sheets of GO and Pd nanoparticles were observed. Moreover, the Pd_np_-TPEPTA_(L)_-GO catalyst was investigated by EDX (Fig. 3d). This analysis clearly shows the presence of Pd and N in the concentration of 30.2 wt% and 46.3 wt%, respectively. According to the Pd wt%, the total mmol of Pd nanoparticles on TPEPTA_(L)_-GO was calculated to be 2.58 mmol g^−1^. Notably, the amount of Pd element in the Pd_np_-TPEPTA_(L)_-GO catalyst measured using atomic absorption spectrometry (AAS) was 28 wt% (see ESI†). To the best of our knowledge, this amount is the highest amount of Pd immobilized on GO or 2D supports. In addition, the amount of TPEPTA ligand supported on the GO sheets was calculated using EDX analysis.^60^ Based on this method, the total amount of TPEPTA ligand detected from the nitrogen content was 1.43 mmol g^−1^ (see ESI†). In order to quantify the amount of Pd in the catalyst, ICP analysis was carried out after its treatment with HCl (37%) and HNO_3_ (65%). Based on the ICP results, the Pd quantity was measured to be 286 mg L^−1^ (286 ppm). This amount is equal to 28.6 wt% (∼2.57 mmol g^−1^). The quantitative analysis is in good agreement with the EDX data.

Then, the Pd_np_-TPEPTA_(L)_-GO catalyst was investigated by TEM in order to investigate the size of Pd nanoparticles and 2D layers of the GO support (Fig. 3e–g). From the TEM images in Fig. 3f and g, the layers of GO were found to be covered by functional groups such as the Pd_np_-TPEPTA complexes. Also, the particle size distribution of the Pd nanoparticles was estimated using Image J software and the average size was about 7.63 ± 0.5 nm.^61^ This data indicates that the Pd nanoparticles did not aggregate on TPEPTA_(L)_-GO and the SDS surfactant assisted this event as well as delete the formation of smaller sized Pd particles.^62^

**Fig. 4:**
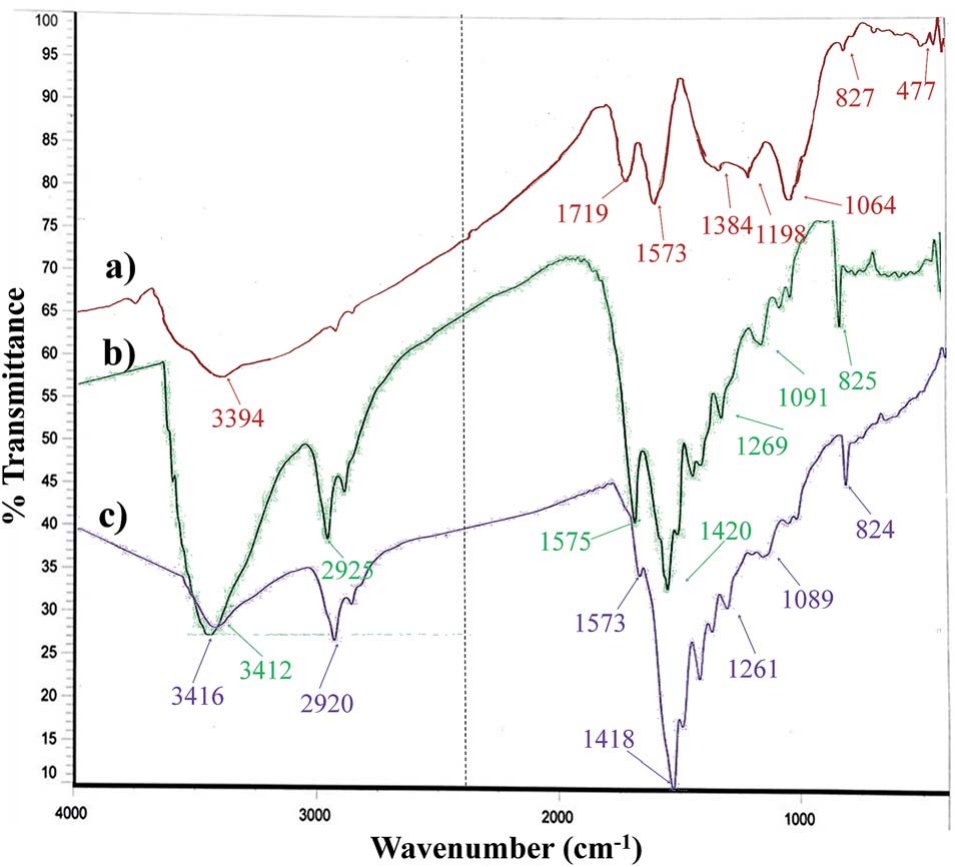
FT-IR spectra of (a) GO, (b) TPEPTA ligand, (c) Pd_np_-TPEPTA_(L)_-GO catalyst.

The FT-IR spectrum of the Pd_np_-TPEPTA_(L)_-GO catalyst and its comparison with GO and TPEPTA ligand shows the peaks that confirm the successful synthesis of the abovementioned catalysts (Fig. 4). Investigating the FT-IR spectrum of GO, the bands appearing at 3394, 1719, 1573, and 1198 cm^−1^ may be attributed to the stretching vibrations of O–H, C=O, C=C, and C–O, respectively.^63^ According to the FT-IR spectrum of the TPEPTA ligand, the absorption peaks observed at 3412, 2925, 1575, and 1091 cm^−1^ are associated with the vibrations of N–H, C_(sp^3^)_–H, C=N, and C–N, respectively.^64,65^ The presence of vibration bands of GO and the TPEPTA ligand in the FT-IR spectrum of Pd_np_-TPEPTA_(L)_-GO confirms the successful synthesis of the target catalyst. The observation of a slight shift (∼4 cm^−1^) in the N–H bands and other peaks is due to the chemical environment of Pd–N coordination.^66–68^

**Fig. 5:**
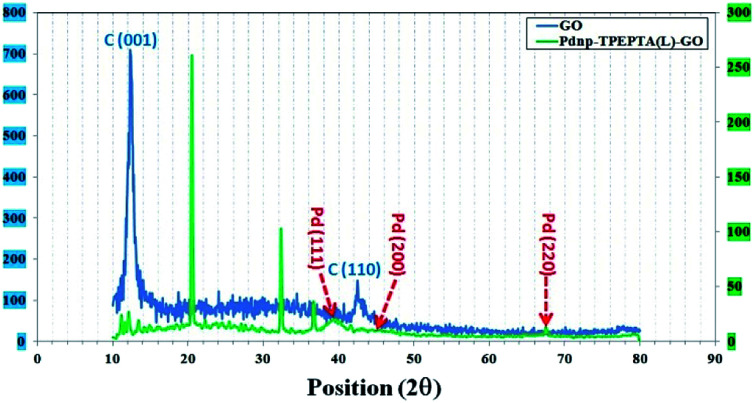
The XRD patterns of GO (blue) and the Pd_np_-TPEPTA_(L)_-GO catalyst (green); the vertical axes at left and right are the intensity values of GO and the Pd_np_-TPEPTA_(L)_-GO catalyst, respectively.

In addition, the crystalline structure of Pd_np_-TPEPTA_(L)_-GO was studied by XRD analysis and the obtained data are displayed in Fig. 5. In the XRD pattern of GO, the peak at 26.4° (002),^69^ which is related to the crystalline structure of graphite, is moved to a lower Bragg angle of 12.1° in the crystalline structure of GO corresponding to the (001) plane with *d*-spacing of 0.92 nm.^70^

The increase in *d*-spacing value is due to the presence of different functional groups including oxygen motif between the graphite layers during the oxidation process using KMnO_4_ and HNO_3_.^69^ However, in the XRD patterns of Pd_np_-TPEPTA_(L)_-GO, the peaks are indexed as the (111) 38.4°, (200) 44.1°, and (220) 67.8° plane, which can be attributed to the face-centered-cubic (fcc) structure of the palladium nanoparticles on TPEPTA_(L)_-GO (JCPDS card 26-1081 and 01-0646).^71,72^ Also, a peak appeared at 21.2° (002), whereas the reflection corresponding to the (001) plane of GO at 12.1° disappeared. This observation confirms the functionalization of GO sheets with TPEPTA ligand.^73^ Furthermore, using Scherrer's equation, the size of the palladium nanoparticles 10.5 nm was calculated.^74^

**Fig. 6:**
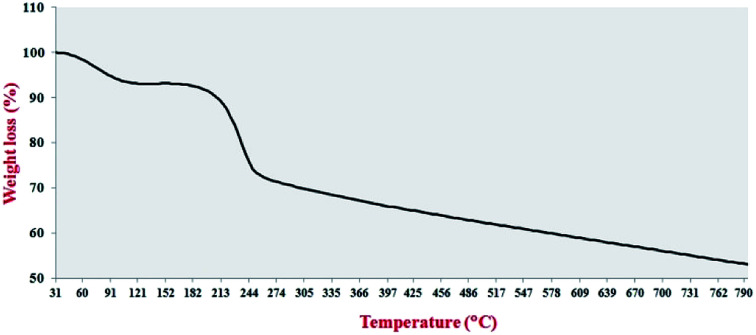
The thermogravimetric analysis curve of Pd_np_-TPEPTA_(L)_-GO. Details of this experiment: sample: 3.0800 mg, range of temperature: 25–800 °C, rate of temperature change: 20 K min^−1^, flow rate of N_2_ gas: 50.0 mL min^−1^, synchronization enabled, sample holder: alumina 70 μL.

TGA analysis was used to investigate the thermal stability of the Pd_np_-TPEPTA_(L)_-GO catalyst (Fig. 6). The TGA of the catalyst shows two main weight losses. The first weight loss was obtained at 88–112 °C, which is attributed to the trapped water and organic solvents.^75^ The second weight loss of the catalyst occurred at 197–239 °C, which can be related to the TPEPTA ligand.^76^ Notably, the remaining non-decomposable sample was approximately 29 wt%, which released the amount of Pd immobilized on the structure of the catalyst. In addition, in accordance with the weight loss amount, the concentration of the TPEPTA ligand per C atoms in the graphene layers was measured, which was found to be one ligand per ninety six carbon atoms (see ESI†).

### Pd_np_-TPEPTA_(L)_-GO-catalyzed Suzuki–Miyaura cross coupling reaction

First, the Suzuki–Miyaura cross coupling reaction of phenylboronic acid (1) and 4-iodoanisole (2) in the presence of Pd_np_-TPEPTA_(L)_-GO catalyst was selected as the model reaction for the optimization of reaction parameters such as type of base, molar ratio of the reactant, loading of the catalyst, type of the solvent, and temperature of the reaction (Table 1). The reaction was performed in the presence of different bases including Et_3_N, NaOH, Na_3_PO_4_, Na_2_CO_3_, and K_2_CO_3_ (Table 1, entries 1–5). However, amongst them, K_2_CO_3_ was found to be the most efficient base.^77^ The main role of the base in the Suzuki reaction is to convert the phenylboronic acid to the more reactive organoborate (PhB(OH)_3_^−^), which facilitates the *trans*-metalation step with the Pd^(II)^-halide motif to afford Pd^(II)^-Ph intermediate.^78^ Then, the model reaction was performed in the presence of different organic solvents (Table 1, entries 6–11). Among the solvent experiments, DMF : H_2_O (2 : 1) was selected as the best solvent for the reaction. In these experiments, the influence of solvent on the catalytic activity of Pd_np_-TPEPTA_(L)_-GO catalyst has been highlighted including the solvent effects on the solubility of the reagents, mass transfer, interactions with the starting materials or products that activate or deactivate the reaction or selectivity in the reaction, solvent interaction with the catalyst, and transition state stabilization.^79^ In addition, DMF is an organic solvent with high dielectric constant with bulk properties similar to water and high ability to stabilize ionic species.^80^ Furthermore, the influence of catalyst amount and the molar ratio of phenylboronic acid and 4-iodoanisole were also considered (Table 1, entries 12, 13). The best results were obtained for the ratio of 1.2 : 1 : 2 for (1) : (2) : base. Also, the effect of catalyst amount (Table 1, entries 14, 15) and temperature (Table 1, entries 16, 17) were explored. Therefore, we obtained and concluded that the optimum reaction conditions were (1) (1.2 mmol), (2) (1 mmol), K_2_CO_3_ (2 mmol), DMF : H_2_O (2 : 1) (6 mL), T: 80 °C, and 30 mg of Pd_np_-TPEPTA_(L)_-GO catalyst (Table 1, entry 5).

**Table 1:** Optimization of the Pd_np_-TPEPTA_(L)_-GO catalyzed Suzuki reaction between 4-iodoanisole and phenylboronic acid^a^

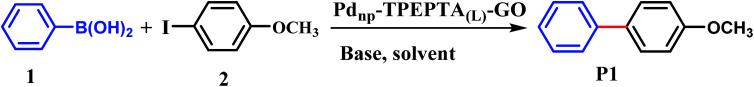							
Entry	Base	Ratio (1 : 2 : base)	Catalyst (mg)	Solvent	*T* (°C)	Time (min)	Yield^b^ (%)
1	Et_3_N	1.2 : 1 : 2	30	DMF : H_2_O (2 : 1)	80	60	20
2	NaOH	1.2 : 1 : 2	30	DMF : H_2_O (2 : 1)	80	40	45
3	Na_3_PO_4_	1.2 : 1 : 2	30	DMF : H_2_O (2 : 1)	80	24	50
4	Na_2_CO_3_	1.2 : 1 : 2	30	DMF : H_2_O (2 : 1)	80	15	75
5	K_2_CO_3_	1.2 : 1 : 2	30	DMF : H_2_O (2 : 1)	80	10	95
6	K_2_CO_3_	1.2 : 1 : 2	30	DMF : H_2_O (1 : 1)	80	15	79
7	K_2_CO_3_	1.2 : 1 : 2	30	H_2_O	Reflux	20	75
8	K_2_CO_3_	1.2 : 1 : 2	30	DMF	80	15	75
9	K_2_CO_3_	1.2 : 1 : 2	30	EtOH : H_2_O (1 : 1)	Reflux	25	65
10	K_2_CO_3_	1.2 : 1 : 2	30	NMP^c^ : H_2_O (1 : 1)	80	20	78
11	K_2_CO_3_	1.2 : 1 : 2	30	NMP^c^ : H_2_O (2 : 1)	80	15	87
12	K_2_CO_3_	1.2 : 1 : 1.5	30	DMF : H_2_O (2 : 1)	80	20	55
13	K_2_CO_3_	1 : 1 : 2	30	DMF : H_2_O (2 : 1)	80	20	70
14	K_2_CO_3_	1.2 : 1 : 2	25	DMF : H_2_O (2 : 1)	80	15	72
15	K_2_CO_3_	1.2 : 1 : 2	35	DMF : H_2_O (2 : 1)	80	10	93
16	K_2_CO_3_	1.2 : 1 : 2	30	DMF : H_2_O (2 : 1)	100	10	95
17	K_2_CO_3_	1.2 : 1 : 2	30	DMF : H_2_O (2 : 1)	60	15	60
18^b^	K_2_CO_3_	1.2 : 1 : 2	80	DMF : H_2_O (2 : 1)	80	25	85

a Reaction conditions: phenylboronic acid (1) (*x* mmol), 4-iodoanisole (2) (*y* mmol), base (*z* mmol), solvent (6 mL).

b Isolated yields.

c *N*-Methylpyrrolidone.

d The Pd-TPEPTA catalyst without graphene oxide as the support was used.

**Scheme 3:**
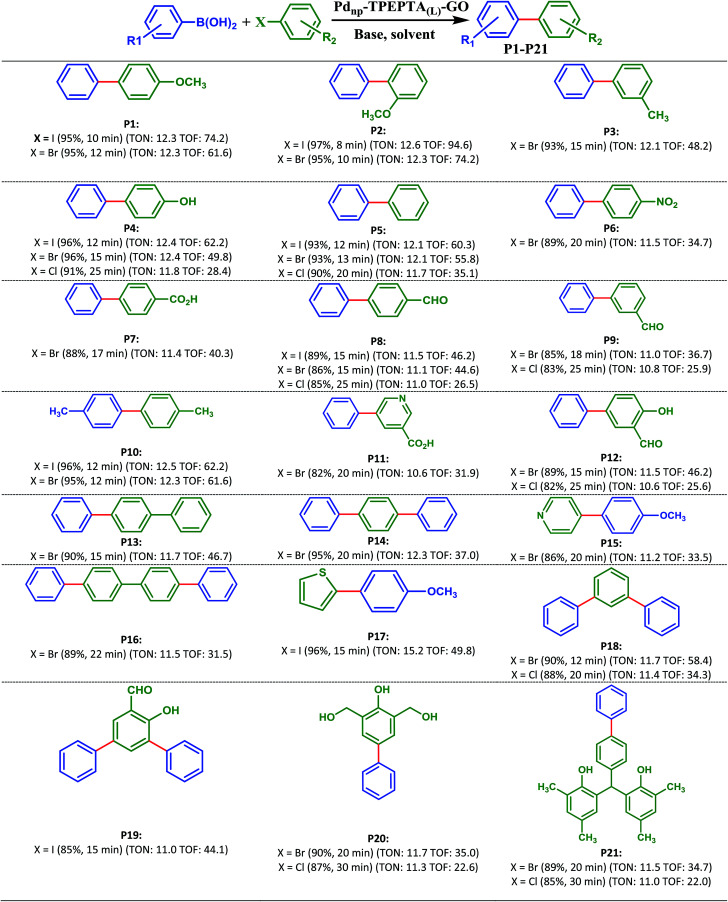
Products of Suzuki–Miyaura reaction catalyzed by Pd_np_-TPEPTA_(L)_-GO. Reaction conditions: aryl(hetero)halide (1 mmol) (shown in green), phenylboronic acids (1.2 mmol) (shown in blue), K_2_CO_3_ (2 mmol), solvent: DMF : H_2_O (2 : 1) (6 mL), Pd_np_-TPEPTA_(L)_-GO (30 mg), T: 80 °C. All yields refer to isolated products. New carbon–carbon bond is shown in red. When the aryl halide had two halogens, phenylboronic acids (2.4 mmol) and K_2_CO_3_ (4 mmol) were used (for products: P14, P16, P18, and P19). TON: turnover number (mmol of product/mmol of catalyst), TOF: turnover frequency ([mmol of product/mmol of catalyst]/time of the reaction) (unit: h^−1^).

With optimization results in hand, the generality and scope of this methodology was evaluated in the synthesis of diverse biaryl units using reactions of different substrates containing I, Br, and/or Cl with phenylboronic acid derivatives (Scheme 3). As shown in Scheme 3, the target products (P1-P21) were obtained in 85–97% and 82–96% yields from the reaction of aryl iodides and bromides involving electron-donating and electron-withdrawing substituents, and phenylboronic acid and 4-methoxyboronic acid as the starting materials, respectively. As we know from the previously reported literature, the presence of electron-withdrawing groups in aryl halide or electron-donating groups in aryl boronic acid enhances the reaction rate and yield of the Suzuki cross coupling reaction due to the facilitation of the rate limiting oxidative addition step.^81–83^ It is notable in this research that the catalyst can improve the Suzuki reaction irrespective of the nature of the substituent (electron-withdrawing or electron-donating) on the aryl halides and aryl boronic acids, and the type of halogen in the aryl halide, compared with the previously reported methods.^21,37,40^

In addition, it is important to note that the aryl chlorides are less reactive than aryl iodides and bromides in the Suzuki cross coupling reaction due to the bond strength. However, many reported papers have focused on the utilization of aryl chlorides in the Suzuki cross coupling reaction.^84–90^ All of the papers report interesting results but research is still appealing in this field. Due to the less reactivity of aryl chlorides, some researchers used a higher amount of the catalyst.^91–94^ In order to study the applicability and efficiency of the Pd_np_-TPEPTA_(L)_-GO catalyst, the Suzuki cross coupling reaction using aryl chlorides to produce products P4, P5, P8, P9, P12, P18, P20, and P21 was studied using the same amount of catalyst as that for the Suzuki reaction using aryl iodides and aryl bromides. The obtained results were very promising. The products were obtained in 82–91% isolated yields in 20–30 minutes. When the reactions were carried out at 120 °C, the isolated yields of the mentioned products increased slightly but the time of the reactions decreased similar to the time of the reactions for aryl iodides and bromides (see ESI-Table 2S†).

**Scheme 4:**
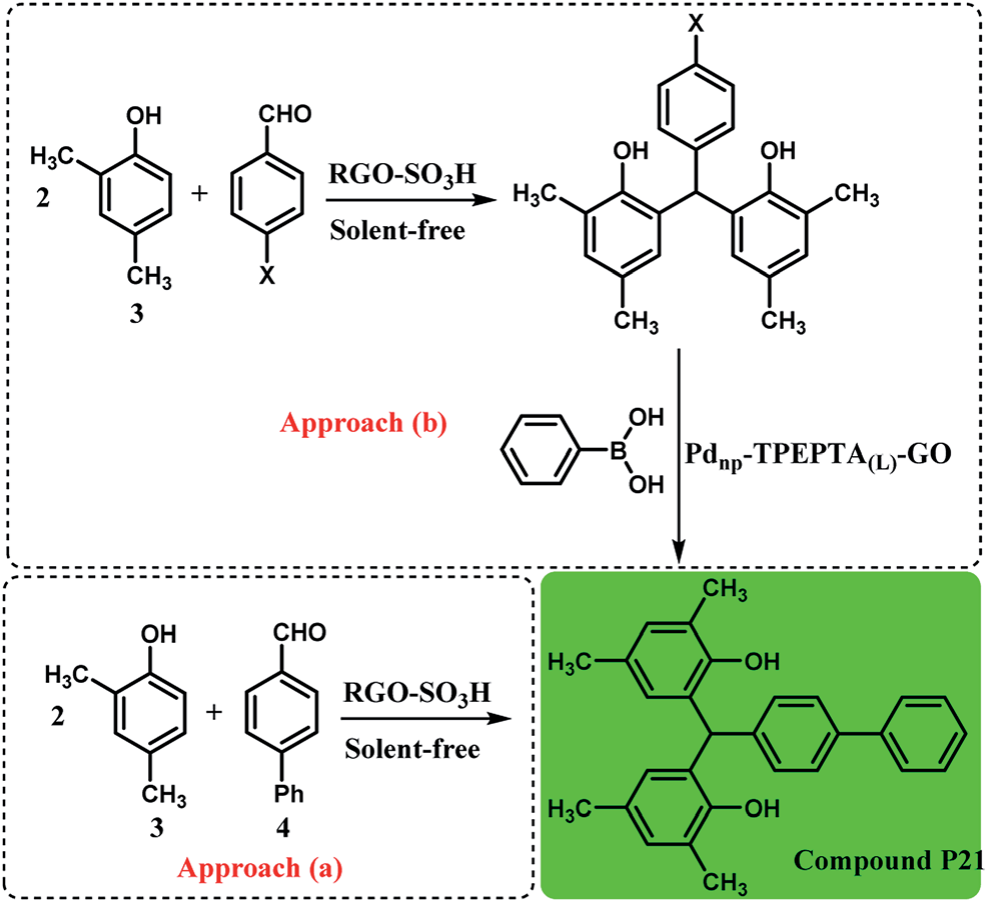
Synthesis of compound 21 using two synthetic pathways, reaction conditions for the approach (a): 2,4-dimethylphenol (3) (6 mmol), 4-phenylbenzaldehyde (4) (2 mmol), solvent-free, *T*: 100 °C, RGO-SO_3_H (sulfonated reduced graphene oxide): 40 mg, time: 2.5 h, isolated yield: 81%. Reaction conditions for approach (b): the condensation step was similar to the approach (a) and X: Br or Cl, the reaction conditions for the Suzuki reaction are the same as the footnote in Scheme 3. Time: 90 min for X: Br and 110 min for X: Cl, and for X: Br and X: Cl the final yields obtained were 91% and 86%, respectively (see ESI†).

Interestingly, the preparation of compound P21 was studied directly from the reaction of 2,4-dimethylphenol (3) and 4-phenylbenzaldehyde (4) by a condensation reaction in the presence of RGO-SO_3_H as the catalyst under solvent free conditions, according to a previous work^71^ and the Suzuki cross coupling reaction through two synthetic pathways (Scheme 4). As can be seen, the approach (b) is more efficient and interesting when the researchers do not use 4-phenylbenzaldehyde. Also, the approach (a) is less unfavorable due to the formation of *O*-alkylated product during the condensation reaction^95,96^ and higher reaction time.

**Scheme 5:**
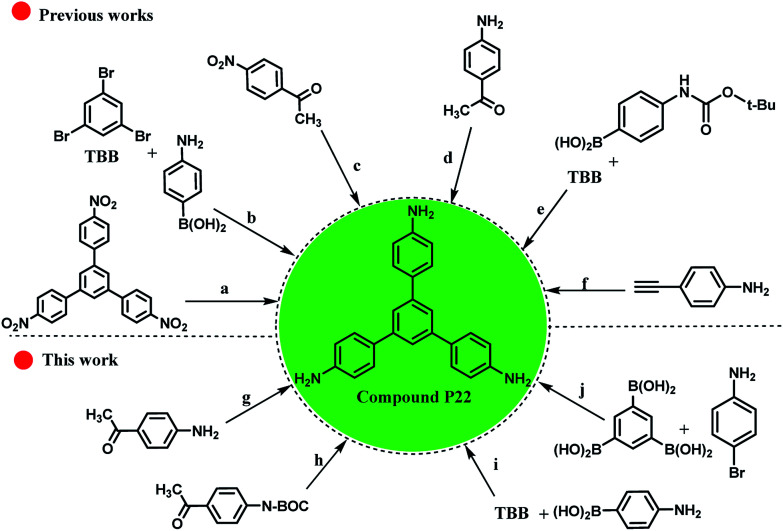
Different approaches for the synthesis of compound P22 from various starting materials (A comparison study between our work and previously reported works). Reaction conditions for (a): 10% palladium on activated charcoal, N_2_H_4_ in ethanol, time: 10.5 h, *T*: 70 °C, yield: 85%.^102^ (b) tetrakis(triphenylphosphine)palladium^(0)^, Aliquat 336, K_2_CO_3_ in H_2_O, toluene, time: 24 h, *T*: 100 °C, inert atmosphere, yield: 62%, TBB is 1,3,5-tribromobenzene.^97^ (c) Two reaction steps, 1: CF_3_SO_3_H, toluene, heating, 2 : 32%, hydrazine hydrate, RANEY^®^ nickel, THF, time: 6 h, *T*: 85 °C.^98^ (d) Toluene-4-sulfonic acid, H_2_O, neat (no solvent), time: 16 h, *T*: 142 °C, yield: 71% (ref. 100) and toluene-4-sulfonic acid, time: 16 h, *T*: 145 °C, yield: 25%.^101^ (e) Two reaction steps, 1: Na_2_CO_3_ in H_2_O, tetrakis(triphenylphosphine)palladium^(0)^ [Pd(PPh_3_)_4_], 1,2-dimethoxy-ethane, time: 18 h, heating, 2: 76%, CF_3_SO_3_H, CH_2_CL_2_, time: 2 h, *T*: 20 °C.^99^ (f) Arachno-[(η^5^-C_5_Me_5_RuCO)_2_B_2_H_6_] in toluene, time: 40 h, *T*: 75 °C, inert atmosphere.^103^ (g) Cyclotrimerization reaction, RGO-SO_3_H (80 mg), toluene, reflux, time: 16 h, yield: 62% (see ESI†) [this work]. (h) Three reaction steps, 1: protection of amino groups using (Boc)_2_O (*tert*-butyloxycarbonyl), 2: cyclotrimerization reaction, RGO-SO_3_H (80 mg), toluene, time: 12 h, reflux, 3: de-protection of N-Boc, total yield: 45%, (see ESI†) [this work]. (i) Pd_np_-TPEPTA_(L)_-GO (30 mg), DMF : H_2_O (2 : 1) (6 mL), K_2_CO_3_, T: 80 °C, time: 1 h, yield: 89% (see ESI†) [this work]. (j): Pd_np_-TPEPTA_(L)_-GO (60 mg), DMF : H_2_O (2 : 1) (6 mL), K_2_CO_3_, T: 100 °C, time: 1.5 h, yield: 80% (see ESI†) [this work].

As shown in Scheme 5, it is possible to obtain the compound P22 (as an important chemical feedstock, CAS-RN: 118727-34-7 and Reaxys-RN: 7822565) using different approaches (Scheme 5, approaches: a–f). Despite much attempts for the synthesis of compound P22 using different starting materials, the successful synthesis with high yields and lower reaction time have been largely overlooked. For example, Schwab and co-workers reported the synthesis of compound P22 using [Pd(PPh_3_)_4_] catalyst in 62% isolated yield (Scheme 5, approach b).^97^ Also, for different approaches with two sequential steps, the total yields were lower than those for the one step approaches (Scheme 5, approaches c and e).^98,99^ Therefore, some experiments were conducted to obtain an efficient method for the preparation of the desired product (Scheme 5, approaches g–j). The cyclotrimerization of three equivalents of 4-aminoacetophenone was carried out using RGO-SO_3_H as the heterogeneous acid catalyst and the target product was produced in 62% yield within 16 h (approach g *vs.* approach d^100,101^ in Scheme 5). However, the presence of NH_2_ group on the starting material caused a higher amount of the used catalyst to be used and the amount of by-product produced was higher during the process. To overcome this problem, we protected the NH_2_ group using *tert*-butyloxycarbonyl ((Boc)_2_O), and then applied RGO-SO_3_H and de-protection of N-Boc to obtain the compound P22 (Scheme 5, approach h). Due to the three reaction steps, the total yield of the desired product was lower than that in our previous approach. These experiments show the significant role of Pd_np_-TPEPTA_(L)_-GO catalyst in the preparation of compound P22 (Scheme 5, approaches i and j). The yield of the desired product increased to 89% isolated yield when the reaction conditions for approach (i) were used. Furthermore, using diverse starting materials, the desired product was obtained in good yield (80%) (Scheme 5, approach j).

**Scheme 6:**
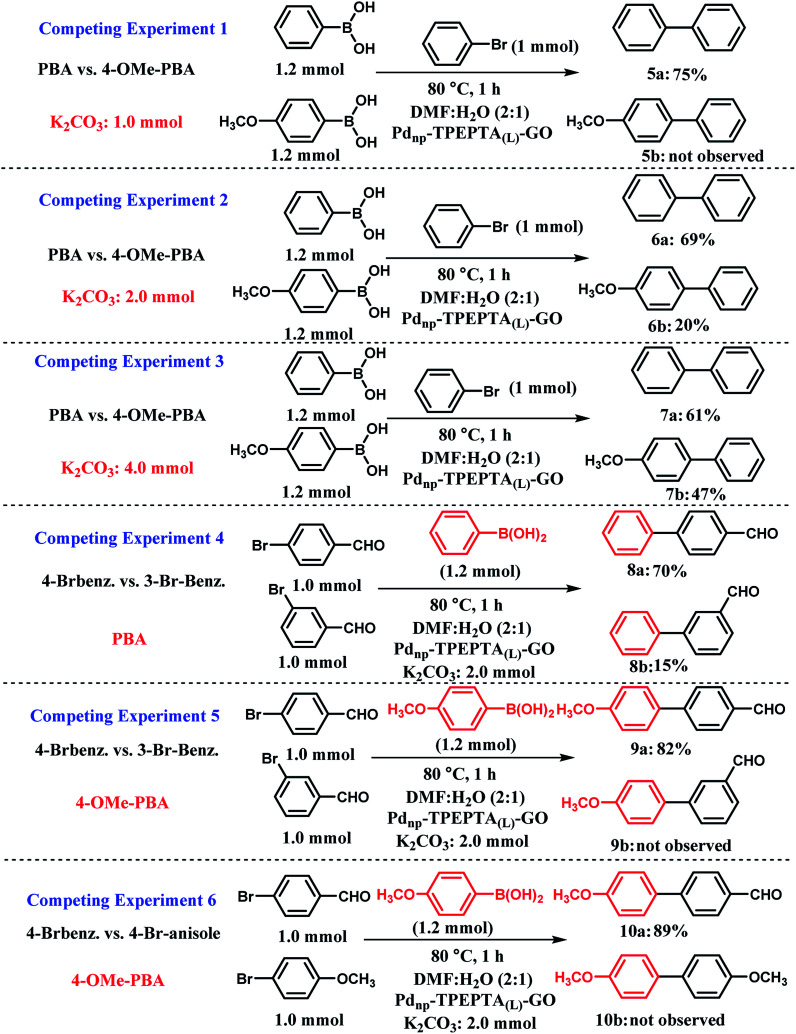
Competing experiments in the Suzuki–Miyaura cross coupling reaction; PBA is phenylboronic acid, 4-OMe-PBA is 4 methoxyphenylboronic acid. Experiments 1–3 for competition between PBA and PBA including electron-donating groups such as OMe at different mmol of K_2_CO_3_ as the base in the presence of the same arylbromide. Experiment 4 for studying the effect of electron-withdrawing group at meta or para positions in the presence of the same phenylboronic acid. Experiment 5 for the investigation of the effect of electron-withdrawing group at meta or para positions in the presence of the same phenylboronic acid including electron-donating groups such as OMe. Experiment 6 for studying the effect of the electron-donating and electron-withdrawing groups on the aryl halide in the Suzuki–Miyaura cross coupling reaction in the presence of the same phenylboronic acid with methoxy group at the para position. General reaction conditions for all the competing experiments: solvent (6 mL), Pd_np_-TPEPTA_(L)_-GO catalyst (30 mg). All the yields refer to the isolated yield. For the typical procedures and separation of the desired compound from the reaction mixture, please see ESI.†

For extended study, a series of competing experiments were also performed to establish the selectivity trends of the presented method (Scheme 6). First, the competing reactions were carried out between two boronic acids including phenylboronic acid (PBA) and 4-methoxyphenylboronic acid (4-OMe-PBA) with 1.2 mmol, 1.0 mmol of bromobenzene, and different amounts of K_2_CO_3_ as the base (Scheme 6, experiments 1–3). The typical procedure for each experiment is illustrated in ESI.† The researchers studying the Pd catalyst have found that a relationship exists between the Suzuki–Miyaura reaction catalytic cycle and acid–base chemistry.^78^ Notably, in the Suzuki cross coupling reaction, the main role of the base such as K_2_CO_3_ is to increase the reactivity of PBA towards the Ph-halide complex by changing it into the corresponding phenylborate (PBO). Hence, the p*K*_a_ values of different PBA were reported in the literature.^104,105^ Therefore, a lower p*K*_a_ value of PBA directly captures the greater tendency to obtain the OH^−^ anion. Among PBA and 4-OMe-PBA, PBA has a lower p*K*_a_ value.^105^ In the competing experiment 1 (CE-1), a limited amount of K_2_CO_3_ was used and good selectivity was observed for the two expected products 5a and 5b. When 1.0 mmol of K_2_CO_3_ was used, PBA with stronger acidity (lower p*K*_a_) reacts more extensively with the OH^−^ anion and the compound 5a was obtained in 75% yield. For further investigation of the amount of base in the selectivity of the present method, we performed CE-2 and CE-3 with 2.0 and 4.0 mmol of K_2_CO_3_, respectively. As shown in these experiments, the selectivity decreases with increase in the stoichiometry of K_2_CO_3_. Additionally, we performed CE-4 and CE-5 for the study of para and meta isomers of arylbromides including electron-withdrawing groups (EWG) in the presence of PBA and 4-OMe-PBA in isolated experiments. In CE-4, 4-bromobenzaldehyde was more reactive relative to 3-bromobenzaldehyde in the presence of PBA. According to the reported literature,^106^ arylhalides with EWG accelerate the oxidative addition step in the mechanism of the Suzuki reaction. Also, this differentiation between 8a and 8b is related to the influence of inductive and resonance effects on the electronic charge stabilization in the Suzuki cross coupling reaction pathway. As shown in Scheme 6, CE-5, 4-OMe-PBA is less reactive towards the base (OH^−^ anion) and a very good selectivity was observed for the two compounds 9a and 9b. Finally, in CE-6, the competition between aryl bromides including EWG and electron-donating groups (EDG) at the para positions were performed and we exclusively observed that aryl bromide including EDG cannot react with 4-OMe-PBO in the presence of aryl bromide including EWG. This observation refers to the mechanistic aspect of the Suzuki reaction progress, thus representing the existence of EDG and EWG on the aryl halides.^106^

**Scheme 7:**
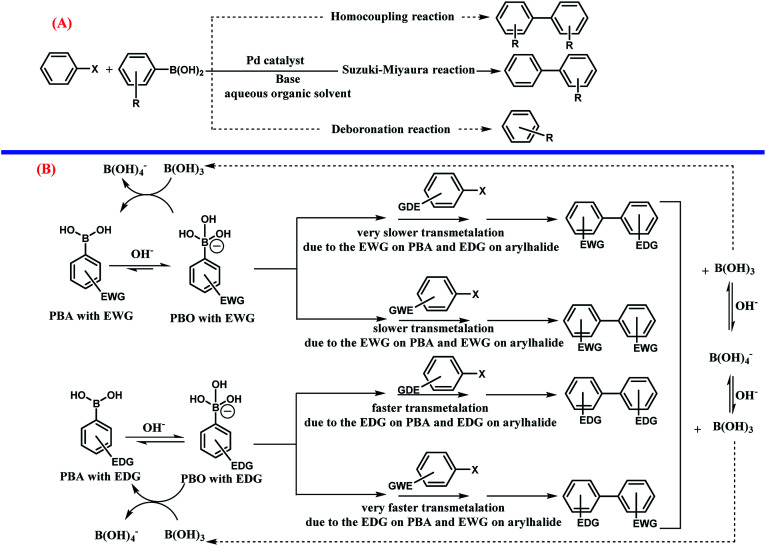
(A) The competing reactions (homocoupling and deboronation) with the Suzuki–Miyaura cross coupling reaction (----: competition reaction,—: main reaction). (B) The effect of electron-withdrawing group (EWG) and electron-donating group (EDG) on phenylboronic acid (PBA), phenylborate (PBO), and aryl halides on the rate of the Suzuki–Miyaura cross coupling reaction.

Usually, there are two competing reactions with the main coupling reaction (Scheme 7, part A). In this study, all the possible products including the homocoupling, deboronation, and Suzuki–Miyaura reactions were monitored during the processes using gas chromatography (GC). These analyses revealed that in all the experiments, the conversion of PBA and aryl halides was 100% and the reaction occurred until completion. Also, in all the experiments, the main product of Suzuki reaction was obtained in large yields and two other byproducts from the homocoupling and deboronation reactions were detected at 0.1–0.5% and 2.3–3.0%, respectively. Interestingly, the present method is highly appealing and selective in limiting the production of by-products relative to the other methods.

It seems that the influence of the substituent plays a dual role in the mechanism of the Suzuki–Miyaura cross coupling reaction (Scheme 7 part B).^78^ As can be seen, PBA with EWG at different positions, especially the para position, increases the acidity of PBA due to delocalization of the negative charge on PBO as a conjugate base by the resonance effect. The EWG substitution on PBA decreased the nucleophilicity of PBO towards the electrophilic Pd complex, which can decrease the rate of the transmetalation step.^107^ The aryl halide with EDG substitution has a slower rate of transmetalation than that for the aryl halide with EWG. The presence of EDG on PBA has the opposite effects. However, according to some previous studies,^78^ a higher rate of transmetalation can be obtained when the EDG and EWG exist on PBA and the aryl halide, respectively.

**Scheme 8:**
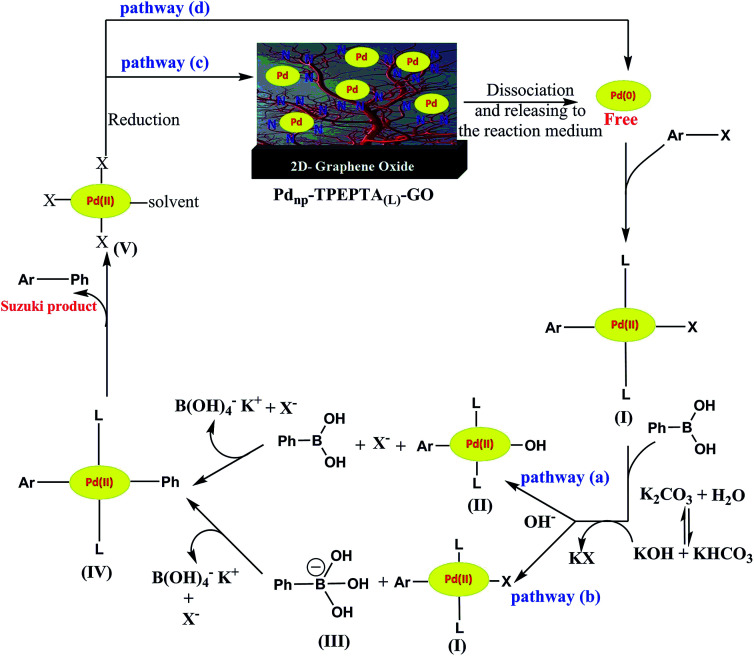
The proposed reaction pathway for Suzuki reaction using the Pd_np_-TPEPTA_(L)_-GO catalyst.

We have proposed a plausible reaction mechanism for this protocol, according to the literature,^108–110^ as shown in Scheme 8. Depending on the reaction conditions such as the concentration and type of the base, the stability of Pd nanoparticles immobilized on the ligand and reduced reaction conditions, the reaction can be performed through different pathways. First, the free Pd(0) was released in the reaction medium near the high SSA of the Pd_np_-TPEPTA_(L)_-GO catalyst. Then, the oxidative addition of Pd(0) to the aryl halide to produce the organopalladium complex intermediate (I) occurred. The two main pathways (a) and (b) can be proposed according to the concentration and power of the base in the reaction medium. When the base has a significant nucleophilicity to replace X as the leaving group from the complex (I), the pathway (a) was followed. However, in the opposite conditions, the base was attached to PBA and formed PBO (III) (pathway b). Subsequently, the migration of the Ph group to intermediates (I) or (II) took place in pathways (a) or (b) to form the intermediate (IV) through the transmetalation step. The Suzuki product was obtained through the reductive elimination of the Pd^(II)^ complex (IV). At this point, the Pd^(II)^ complex (V) was reduced and free Pd(0) can follow two main pathways including rebounding to the catalyst *via* pathway (c) or the initiation of the next catalytic cycle *via* pathway (d).

The greatest advantages of Pd_np_-TPEPTA_(L)_-GO catalyst are the ease of separation and compatibility with the organic compounds as reactants due to the large organic ligand supported on GO as the inorganic support. Also, the reusability and recyclability of Pd_np_-TPEPTA_(L)_-GO catalyst is very important from the economic, environmental, and industrial points of view. Therefore, the reusability of the Pd_np_-TPEPTA_(L)_-GO catalyst was tested in the Suzuki–Miyaura cross coupling reaction for the preparation of compound P1 under the optimized conditions within 10 minutes. The Pd_np_-TPEPTA_(L)_-GO catalyst showed a reusability of at least six runs without a remarkable decrease in its catalytic activity performance (Table 2). As shown in this table, the first catalytic run resulted in the production of 95% of the desired product within 10 minutes. In the 5th catalytic run, approximately 87% of the final product was obtained. According to the recovered catalyst values in each run, about 97% of the Pd_np_-TPEPTA_(L)_-GO catalyst remained until the 6th run. However, the decreasing catalytic activity in the 6th run can be related to the lost catalyst during catalyst recovery and not to palladium leaching. During the reusability experiments, the Pd content of the catalyst decreased by only 6 ppm (about 2.3%) of the total Pd content immobilized on TPEPTA_(L)_-GO. Therefore, this observation showed that the Pd_np_-TPEPTA_(L)_-GO catalyst is a robust and satisfactory heterogeneous catalyst. For the completion of our observation related to the Pd leaching, the hot filtration study for this catalyst was performed and the catalyst immediately following the end of the reaction was isolated. According to the ICP analysis obtained for the hot filtration test, the Pd detected in the 1st, 3rd, and 6th runs were 0.08, 0.1, and 0.06 ppm, respectively. After the isolation of the catalyst, the hot filtration reaction mixture was charged with iodobenzene and PBA. The GC yields for the 1st, 3rd, and 6th runs were only < 2%. This experiment strongly revealed that the leaching of Pd nanoparticles during the reaction progress is very low and the Pd_np_-TPEPTA_(L)_-GO catalyst is a reusable and air-stable heterogeneous catalyst in practice. For further investigation of the used catalyst after the 6th run, the morphology of Pd_np_-TPEPTA_(L)_-GO was studied and the catalyst remained mostly unchanged after use in the Suzuki–Miyaura cross coupling reaction, according to the FT-IR and TEM studies (see ESI†).

**Table 2:** Reusability of the Pd_np_-TPEPTA_(L)_-GO catalyst in the synthesis of compound P1^a^

Run	First	Second	Third	Fourth	Fifth	Sixth
Time (min)	10	10	10	10	10	10
Yield (%)^b^	95	95	93	92	90	87
TON	12.3	12.3	12.1	11.9	11.7	11.3
TOF (h^−1^)	74.1	74.1	72.9	71.7	70.5	68.1
Recovered catalyst (%)	>99	>99	>98	>98	>97	>97
Pd amount (ppm)	258	257	ND	ND	253	252
Pd leached after hot filtration test (ppm)	0.08	ND^c^	0.1	ND	ND	0.06
Yield of hot filtration test (%)^d^	<2	ND	<2	ND	ND	<2

a 4-Iodoanisole (1 mmol), phenylboronic acids (1.2 mmol), K_2_CO_3_ (2 mmol), solvent: DMF : H_2_O (2 : 1) (6 mL), Pd_np_-TPEPTA_(L)_-GO (30 mg), *T*: 80 °C.

b Isolated yields.

c Not determined.

d GC yield.

## Conclusion

In this research, we have introduced an efficient synthetic methodology for the synthesis of biaryl units using aryl halide and phenylboronic acid derivatives. A heterogeneous Pd catalyst system was developed by immobilizing Pd on TPEPTA as a ligand with high N-ligation sites. The complex was supported on graphene oxide through 3-chloropropyltrimethoxysilane. The synthetic route for the preparation of Pd_np_-TPEPTA_(L)_-GO catalyst is simple and the starting materials are available and cheap. The Pd_np_-TPEPTA_(L)_-GO catalyst shows high catalytic activity in the Suzuki–Miyaura cross coupling reaction. A range of aryl or heteroaryl halides including electron-withdrawing and electron-donating substituents and different halogens were employed. The catalyst was efficient and reusable for three types of halogens involving Cl, Br, and I in the aryl halides. The Pd_np_-TPEPTA_(L)_-GO catalyst was recyclable for six runs. Moreover, a series of competing experiments was performed to establish the selectivity trends of the presented method.

## Experimental

### General

All the solvents, materials, and reagents were used without further purification. The melting points of the synthesized compounds were determined with a Thermo-scientific micro apparatus in capillary tubes and are uncorrected. Graphite powder and the inorganic materials were purchased from Merck chemical company. The NMR spectra of the synthesized compounds were recorded on a Brucker DRX-400 MHz spectrometer. *δ* (ppm) chemical shifts are given relative to CDCl_3_ and DMSO-d_6_ as the solvents: the reference peak for CDCl_3_ were 7.24 ppm (^1^H NMR) and 77.0 ppm (^13^C NMR), and for DMSO-d_6_ were 2.47 ppm (^1^H NMR) and 39.94 ppm (^13^C NMR). The FT-IR spectra were recorded on a PerkinElmer 781 spectrophotometer in the range of 400–4000 cm^−1^ using KBr pellets.

### General procedure for the Suzuki–Miyaura cross coupling reaction using Pd_np_-TPEPTA_(L)_-GO catalyst

Aryl halide (1.0 mmol), phenyl boronic acid derivatives (1.2 mmol), Pd_*np*_-TPEPTA_(L)_-GO (30 mg), K_2_CO_3_ (2.0 mmol), and DMF : H_2_O (2 : 1) (6.0 mL) were placed in a 25 mL round-bottom-flask equipped with a condenser and magnetic stirring bar, and heated at 80 °C. The progress of the reaction was monitored using TLC until 100% conversion of the aryl halide was confirmed. After the completion of the reaction, 5.0 mL of hot water and 5.0 mL of ethyl acetate were added to the reaction mixture. The Pd_np_-TPEPTA_(L)_-GO catalyst was separated under reduced pressure using a vacuum pomp over sintered-glass grade-4. The organic solution was evaporated on a rotary evaporator and the crude product was obtained. For further purification of each of the synthesized compounds, different techniques were applied (see ESI†).

## Conflicts of interest

The authors declare no conflict of interest.

## Abbreviation

TPEPTA: Tris(pentaethylene-pentamine)triazine
GO: Graphene oxide
1D: One-Dimensional
2D: Two-Dimensional
3D: Three-Dimensional
SSA: Specific Surface Area
TCT: 2,4,6-Trichloro-1,3,5-triazine
PEHA: Pentaethylenehexamine
SDS: Sodium dodecyl sulfate
AAS: Atomic absorption spectrometry
PBA: Phenylboronic acid
4-OMe-PBA: 4-Methoxyphenylboronic acid
PBO: Phenylborate
EWG: Electron-withdrawing group
EDG: Electron-donating group

## Supplementary Material

RA-009-C9RA04511B-s001

## References

[cit1] Anderson G. and Hartley F., Chemistry of Platinum Group Metals, Recent Developments, Elsevier, 1st edn, 1991

[cit2] Wilkinson G. and Gillard R. D., Comprehensive coordination chemistry: the synthesis, reactions, properties & applications of coordination compounds, Middle transition elements, Pergamon, 1987

[cit3] Cotton S. A. , in Chemistry of Precious Metals, Springer Netherlands, Dordrecht, 1997, pp. 173–272, 10.1007/978-94-009-1463-6_3

[cit4] Stevens P. D., Li G., Fan J., Yen M., Gao Y. (2005). Chem. Commun..

[cit5] Peris E., Crabtree R. H. (2004). Coord. Chem. Rev..

[cit6] Fortman G. C., Nolan S. P. (2011). Chem. Soc. Rev..

[cit7] Blaser H.-U., Indolese A., Schnyder A., Steiner H., Studer M. (2001). J. Mol. Catal. A: Chem..

[cit8] Toebes M. L., van Dillen J. A., de Jong K. P. (2001). J. Mol. Catal. A: Chem..

[cit9] Mubofu E. B., Clark J. H., Macquarrie D. J. (2001). Green Chem..

[cit10] Navalon S., Dhakshinamoorthy A., Alvaro M., Antonietti M., García H. (2017). Chem. Soc. Rev..

[cit11] Lyu Q., Yan H., Li L., Chen Z., Yao H., Nie Y. (2017). Polymers.

[cit12] Wang B., Yan T., Chang T., Wei J., Zhou Q., Yang S., Fang T. (2017). Carbon.

[cit13] Wang C., Zhang L., Guo Z., Xu J., Wang H., Zhai K., Zhuo X. (2010). Microchim. Acta.

[cit14] Kou R., Shao Y., Wang D., Engelhard M. H., Kwak J. H., Wang J., Viswanathan V. V., Wang C., Lin Y., Wang Y., Aksay I. A., Liu J. (2009). Electrochem. Commun..

[cit15] Xiang Q., Yu J., Jaroniec M. (2012). Chem. Soc. Rev..

[cit16] Bolotin K. I., Sikes K. J., Jiang Z., Klima M., Fudenberg G., Hone J., Kim P., Stormer H. L. (2008). Solid State Commun..

[cit17] Fu Q., Bao X. (2017). Chem. Soc. Rev..

[cit18] Deng D., Novoselov K. S., Fu Q., Zheng N., Tian Z., Bao X. (2016). Nat. Nanotechnol..

[cit19] Dikin D. A., Stankovich S., Zimney E. J., Piner R. D., Dommett G. H. B., Evmenenko G., Nguyen S. T., Ruoff R. S. (2007). Nature.

[cit20] Zhu Y., Murali S., Cai W., Li X., Suk J. W., Potts J. R., Ruoff R. S. (2010). Adv. Mater..

[cit21] Huang Y., Wei Q., Wang Y., Dai L. (2018). Carbon.

[cit22] Sengupta D., Pandey M. K., Mondal D., Radhakrishna L., Balakrishna M. S. (2018). Eur. J. Inorg. Chem..

[cit23] Elazab H. A., Moussa S., Siamaki A. R., Gupton B. F., El-Shall M. S. (2017). Catal. Lett..

[cit24] Elazab H. A., Siamaki A. R., Moussa S., Gupton B. F., El-Shall M. S. (2015). Appl. Catal., A.

[cit25] Wang C., Salmon L., Ciganda R., Yate L., Moya S., Ruiz J., Astruc D. (2017). Chem. Commun..

[cit26] Wang X., Chen W., Yan L. (2014). Mater. Chem. Phys..

[cit27] Song H.-q., Zhu Q., Zheng X.-j., Chen X.-g. (2015). J. Mater. Chem. A.

[cit28] Singh V. V., Kumar U., Tripathi S. N., Singh A. K. (2014). Dalton Trans..

[cit29] Shi X., Cai C. (2018). New J. Chem..

[cit30] Sharavath V., Ghosh S. (2014). RSC Adv..

[cit31] Scheuermann G. M., Rumi L., Steurer P., Bannwarth W., Mülhaupt R. (2009). J. Am. Chem. Soc..

[cit32] Sarvestani M., Azadi R. (2017). Appl. Organomet. Chem..

[cit33] Rana S., Maddila S., Yalagala K., Jonnalagadda S. B. (2015). Appl. Catal.,A.

[cit34] Qu K., Wu L., Ren J., Qu X. (2012). ACS Appl. Mater. Interfaces.

[cit35] Nie R., Shi J., Du W., Hou Z. (2014). Appl. Catal., A.

[cit36] Moghadam M., Salavati H., Pahlevanneshan Z. (2018). J. Iran. Chem. Soc..

[cit37] Mahdavi H., Rahmani O. (2016). Catal. Lett..

[cit38] Joshi H., Sharma K. N., Sharma A. K., Singh A. K. (2014). Nanoscale.

[cit39] Gómez-Martínez M., Buxaderas E., Pastor I. M., Alonso D. A. (2015). J. Mol. Catal. A: Chem..

[cit40] Fareghi-Alamdari R., Haqiqi M. G., Zekri N. (2016). New J. Chem..

[cit41] Hashemi Fath R., Hoseini S. J. (2017). J. Organomet. Chem..

[cit42] Jafar Hoseini S., Dehghani M., Nasrabadi H. (2014). Catal. Sci. Technol..

[cit43] Fareghi-Alamdari R., Golestanzadeh M., Bagheri O. (2016). RSC Adv..

[cit44] Li X., Zhao Q., Feng X., Pan L., Wu Z., Wu X., Ma T., Liu J., Pan Y., Song Y., Wu M. (2019). ChemSusChem.

[cit45] Golestanzadeh M., Naeimi H., Zahraie Z. (2016). ChemistrySelect.

[cit46] Naeimi H., Golestanzadeh M. (2015). New J. Chem..

[cit47] Golestanzadeh M., Naeimi H., Zahraie Z. (2017). Mater. Sci. Eng., C.

[cit48] Naeimi H., Golestanzadeh M., Zahraie Z. (2016). Int. J. Biol. Macromol..

[cit49] Dainyte A., Gudeika D., Buika G., Grazulevicius J. V. (2014). Mol. Cryst. Liq. Cryst..

[cit50] Lupton J. M., Hemingway L. R., Samuel I. D. W., Burn P. L. (2000). J. Mater. Chem..

[cit51] Bandgar B. P., Joshi N. S., Kamble V. T. (2006). Tetrahedron Lett..

[cit52] Blotny G. (2006). Tetrahedron.

[cit53] Samaritani S., Signore G., Malanga C., Menicagli R. (2005). Tetrahedron.

[cit54] Gemeay A. H., El-Halwagy M. E., El-Sharkawy R. G., Zaki A. B. (2017). J. Environ. Chem. Eng..

[cit55] Sobhani S., Zarifi F., Skibsted J. (2017). New J. Chem..

[cit56] Doughty H. W. (1924). J. Am. Chem. Soc..

[cit57] Mohanazadeh F., Amini H. (2010). Bull. Korean Chem. Soc..

[cit58] Baier G., Baki A., Tomcin S., Mailänder V., Alexandrino E., Wurm F., Landfester K. (2014). Macromol. Symp..

[cit59] Mafuné F., Kohno J.-y., Takeda Y., Kondow T., Sawabe H. (2000). J. Phys. Chem. B.

[cit60] Nielsen R., Kingshott P., Uyar T., Hacaloglu J., Larsen K. L. (2011). Surf. Interface Anal..

[cit61] Pyrz W. D., Buttrey D. J. (2008). Langmuir.

[cit62] Mortazavi-Derazkola S., Zinatloo-Ajabshir S., Salavati-Niasari M. (2015). RSC Adv..

[cit63] Li D., Müller M. B., Gilje S., Kaner R. B., Wallace G. G. (2008). Nat. Nanotechnol..

[cit64] Clougherty L., Sousa J., Wyman G. (1957). J. Org. Chem..

[cit65] Rao C. N. R., Venkataraghavan R. (1962). Spectrochim. Acta.

[cit66] Durig J. R., Layton R., Sink D. W., Mitchell B. R. (1965). Spectrochim. Acta.

[cit67] Mizuguchi M., Nara M., Ke Y., Kawano K., Hiraoki T., Nitta K. (1997). Eur. J. Biochem..

[cit68] Wahl R., Engelhardt H., Pompe W., Mertig M. (2005). Chem. Mater..

[cit69] Li Z. Q., Lu C. J., Xia Z. P., Zhou Y., Luo Z. (2007). Carbon.

[cit70] Some S., Kim Y., Yoon Y., Yoo H., Lee S., Park Y., Lee H. (2013). Sci. Rep..

[cit71] Naeimi H., Golestanzadeh M. (2014). RSC Adv..

[cit72] Khalafi-Nezhad A., Panahi F. (2011). Green Chem..

[cit73] Anasdass J. R., Kannaiyan P., Raghavachary R., Gopinath S. C. B., Chen Y. (2018). PLoS One.

[cit74] Borchert H., Shevchenko E. V., Robert A., Mekis I., Kornowski A., Grübel G., Weller H. (2005). Langmuir.

[cit75] Park S., An J., Potts J. R., Velamakanni A., Murali S., Ruoff R. S. (2011). Carbon.

[cit76] Guo S., Dong S., Wang E. (2010). ACS Nano.

[cit77] Zhang H., Kwong F. Y., Tian Y., Chan K. S. (1998). J. Org. Chem..

[cit78] Lima C. F. R. A. C., Rodrigues A. S. M. C., Silva V. L. M., Silva A. M. S., Santos L. M. N. B. F. (2014). ChemCatChem.

[cit79] Dyson P. J., Jessop P. G. (2016). Catal. Sci. Technol..

[cit80] Anslyn E. V. and Dougherty D. A., Modern physical organic chemistry, University science books, 2006

[cit81] Fairlamb I. J. S., Kapdi A. R., Lee A. F. (2004). Org. Lett..

[cit82] Littke A. F., Dai C., Fu G. C. (2000). J. Am. Chem. Soc..

[cit83] Guan Z., Li B., Hai G., Yang X., Li T., Tan B. (2014). RSC Adv..

[cit84] Arvela R. K., Leadbeater N. E. (2005). Org. Lett..

[cit85] LeBlond C. R., Andrews A. T., Sun Y., Sowa J. R. (2001). Org. Lett..

[cit86] Cho S.-D., Kim H.-K., Yim H.-s., Kim M.-R., Lee J.-K., Kim J.-J., Yoon Y.-J. (2007). Tetrahedron.

[cit87] John A., Shaikh M. M., Ghosh P. (2010). Inorg. Chim. Acta.

[cit88] Xu L.-Y., Liu C.-Y., Liu S.-Y., Ren Z.-G., Young D. J., Lang J.-P. (2017). Tetrahedron.

[cit89] Liu L., Wang W., Xiao C. (2014). J. Organomet. Chem..

[cit90] Wolfe J. P., Singer R. A., Yang B. H., Buchwald S. L. (1999). J. Am. Chem. Soc..

[cit91] Corma A., García H., Leyva A. (2006). J. Catal..

[cit92] Mino T., Shirae Y., Sakamoto M., Fujita T. (2005). J. Org. Chem..

[cit93] Kostas I. D., Andreadaki F. J., Kovala-Demertzi D., Christos P., Demertzis M. A. (2005). Tetrahedron Lett..

[cit94] Deng C.-L., Guo S.-M., Xie Y.-X., Li J.-H. (2007). Eur. J. Org. Chem..

[cit95] Fareghi-Alamdari R., Golestanzadeh M., Zekri N., Mavedatpoor Z. (2015). J. Iran. Chem. Soc..

[cit96] Fareghi-Alamdari R., Mansouri F., Golestanzadeh M., Zekri N. (2018). Curr. Org. Chem..

[cit97] Schwab M. G., Hamburger M., Feng X., Shu J., Spiess H. W., Wang X., Antonietti M., Müllen K. (2010). Chem. Commun..

[cit98] Yan P., Chowdhury A., Holman M. W., Adams D. M. (2005). J. Phys. Chem. B.

[cit99] Ishi-i T., Kuwahara R., Takata A., Jeong Y., Sakurai K., Mataka S. (2006). Chem.–Eur. J..

[cit100] Zhao Y., Li J., Li C., Yin K., Ye D., Jia X. (2010). Green Chem..

[cit101] Gattuso G., Grasso G., Marino N., Notti A., Pappalardo A., Pappalardo S., Parisi M. F. (2011). Eur. J. Org. Chem..

[cit102] Xu W.-Q., Fan Y.-Z., Wang H.-P., Teng J., Li Y.-H., Chen C.-X., Fenske D., Jiang J.-J., Su C.-Y. (2017). Chem.–Eur. J..

[cit103] Geetharani K., Tussupbayev S., Borowka J., Holthausen M. C., Ghosh S. (2012). Chem.–Eur. J..

[cit104] Hall D. , Preparation and Applications in Organic Synthesis, Medicine and Materials, Wiley, VCH, 2011, vol. 1 and 2

[cit105] Yan J., Springsteen G., Deeter S., Wang B. (2004). Tetrahedron.

[cit106] Zim D., Monteiro A. L., Dupont J. r. (2000). Tetrahedron Lett..

[cit107] Lima C. F. R. A. C., Rodriguez-Borges J. E., Santos L. M. N. B. F. (2011). Tetrahedron.

[cit108] Miyaura N. (2002). J. Organomet. Chem..

[cit109] Carrow B. P., Hartwig J. F. (2011). J. Am. Chem. Soc..

[cit110] Amatore C., Le Duc G., Jutand A. (2013). Chem.–Eur. J..

