# Supplementary material for: Palladium decorated on a new dendritic complex with nitrogen ligation grafted to graphene oxide: fabrication, characterization, and catalytic application
Source: RSC Adv. 2019 Sep 2;9(47):27560–73. doi: 10.1039/c9ra04511b (PMC9070579; doi:10.1039/c9ra04511b)
Supplement: RA-009-C9RA04511B-s001 [file RA-009-C9RA04511B-s001.pdf]

Electronic Supplementary Material (ESI) for RSC Advances.
This journal is © The Royal Society of Chemistry 2019

Supporting Information for

Palladium Decorated on New Dendritic Complex with Nitrogen Ligation Grafted to Graphene Oxide: Fabrication, Characterization and Catalytic Application

Mohsen Golestanzadeh[a,b]; Hossein Naeimi[a]*

a) *Departetment of Organic Chemistry, Faculty of Chemistry, University of Kashan, Kashan, 8731751167, I. R. Iran*

b) *Child Growth and Development Research Center, Research Institute for Primordial Prevention of Non-Communicable Disease, Isfahan University of Medical Sciences, Isfahan, 8174673461, I. R. Iran*

Email: naeimi@kashanu.ac.ir; or golestanzadeh@grad.kashanu.ac.ir; Tel: +98-31-55912388; Fax: +98-31-55912397

Contents **Page**

Experimental SI-3

Optimization study of the reaction of TCT and PEHA SI-3

Characterization of TPEPTA ligand SI-3

Determination of chlorine atom on GO using Mohr's method SI-44

Determination of Pd nanoparticles using AAS SI-5

Determination of TPEPTA ligand using EDX analysis SI-5

The extent of TPEPTA ligand per C atoms in graphene layers using TGA analysis SI-6

Products of Suzuki-Miyaura reaction from aryl chloride at 120 °C(A comparison study with 80 °C) SI-6

Synthesis of compound P21 .... SI-6

Synthesis of compound P22 .... SI-8

Typically procedures for competing experiments (CE) SI-10

Characterization of reused Pd_{np}-$TPEPTA_{(L)}$-GO catalyst SI-13

The synthetic routes for the preparation of Pd_{np}-$TPEPTA_{(L)}$-GO catalyst SI-14

Spectroscopic and Physical data of the synthesized compounds SI-16

Hard copies of ^{1}H NMR and ^{13}C NMR of the synthesized compounds SI-25

Supporting References: SI-43

Experimental

Optimization study of the reaction of TCT and PEHA

Table 1S, Optimization of the reaction conditions for the preparation of TPEPTA (Ligand)

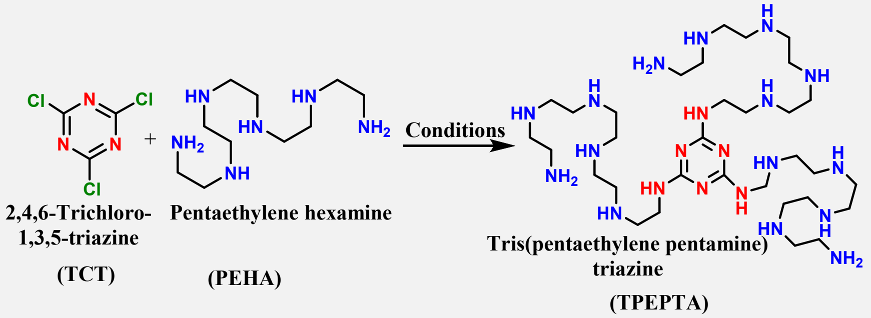

Entry	TCT (mmol)	PEHA (mmol)	Base	Temperature (°C)	Solvent	Time (h)	Yield (%)[a]
1	9	27	Et_3N	60	DMF	12	43
2	9	30	Et_3N	60	DMF	12	50
3	9	33	Et_3N	60	DMF	12	50
4	9	30	Et_3N	80	DMF	12	68
5	9	30	Et_3N	100	DMF	12	67
6	9	30	Pyridine	80	DMF	12	28
7	9	30	N,N-dimethylaniline	80	DMF	12	45
8	9	30	Et_3N	80	H_2O	12	15
9	9	30	Et_3N	80	H_2O/DMF	12	30
10	9	30	Et_3N	80	EtOH	12	40

a)Isolated yields

Into a canonical flask (250 mL), a mixture of TCT (9 mmol, 1.66 g) and PEHA (30 mmol, 6.96 g) was stirred in DMF (25 mL) and 0.1 mL of Et_3N at 80 °C for 12 h under N_2 gas as an inert atmosphere. After completion of the reaction, the content of the flask allowed to cool down to room temperature. The precipitate (dark red-orange) was filtered over sinter glass (G-4) and washed thoroughly with aqueous solution of $NaHCO_3$ (10%) and large amount of warm distilled water. The obtained solid product (TPEPTA) was dried at 80 °C for 24 h under vacuum conditions (Isolated yields: 68%, 4.7 g).

Characterization of TPEPTA ligand

tris(pentaethylene-pentamine)triazine (TPEPTA) (Ligand)

Melting point: 330 °C

Color: dark red-orange

IR (KBr): ῡ (cm^{-1}) 3349, 2929, 1614, 1522, 1489, 998. Anal. Calcd for $C_{33}H_{81}N_{21}$: C, 51.33; H, 10.57; N, 38.09; Found, C, 50.97, H, 10.14, N, 37.94. ^{1}H NMR (400 MHz) (DMSO-d_6): 1.38-1.42 (broad multiplet, 18 H, N$\underline{H_2}$ and N$\underline{H}$ in PEHA), 2.47-2.50 (multiplet, 36 H, C$\underline{H_2}$ in PEHA), 2.63-2.66 (18 H, multiplet, C$\underline{H_2}$ in PEHA), 3.38 (triplet, 6 H, C$\underline{H_2}$ in PEHA), 6.94 (broad multiplet, 3H, N$\underline{H}$ in TCT). ^{13}C NMR (100 MHz) (DMSO-d_6):162.1, 159.3, 65.2, 51.2, 48.5, 47.0, 46.7, 40.8.

Determination of chlorine atom on GO using Mohr's method

The Mohr's method was used to determine the amount of chlorine atom of 3-chloropropylsilyl group supported on GO. At first, 100 mg of 3-chloropropylsilyl group supported on GO was dispersed in distilled water (10 mL) and 10.0 mL of $NaHCO_3$ (0.005 M) was added to the solution of functionalized GO. Then, the reaction mixture was sonicated for 2 min under ultrasound irradiation and subsequently the reaction mixture was stirred for 1 h at room temperature. Next, the reaction mixture was filtered over sinter glass (G-4) and thoroughly washed several times with distilled water. Therefore, the filtrate solution has sodium chloride as well as excess amount of sodium hydrogen carbonate. At this time, we could determine the amount of chlorine ions in solution using Mohr's method. The pH of the sample solutions should be between 6.5 and 10 (the pH was checked). This method determines the chloride ion concentration of obtained solution by titration with silver nitrate ($AgNO_3$). As the silver nitrate solution is slowly added, a precipitate of silver chloride forms. The end point of the titration occurs when all the chloride ions are precipitated. Then additional silver ions react with the chromate ions of the indicator, potassium chromate (K_2CrO_4), to form a red-brown precipitate of silver chromate. For Mohr's method, the following solutions are needed. Silver nitrate solution: (0.1 mol L^{-1}) If possible, dry 5.0 g of $AgNO_3$ for 2 h at 100°C and allow cooling. Accurately weigh about 4.25 g of solid $AgNO_3$ and dissolve it in 25.0 mL of distilled water in a conical

flask. Store the solution in a brown bottle. Potassium chromate indicator solution (approximately 0.25 mol L^{-1}) Dissolving 1.0 g of K_2CrO_4 in 20.0 mL of distilled water. In according to Mohr's method, the total of chlorine ion in this solution was 0.95 mmol per 100 g of functionalized graphene. Therefore, we obtained the total density of chloropropylsilyl groups on GO was approximately 0.95 mmol g^{-1}.

Determination of Pd nanoparticles using AAS

The content of Pd nanoparticles on $TPEPTA_{(L)}$-GO was measured according to the previous reported articles [1,2]. A Perkin-Elmer 3110 model atomic absorption spectrometer equipped with an air-acetylene burner and a palladium hollow cathode lamp operated at 20 mA was used for the determination of palladium without background correction. The operating conditions were as follows: wave length: 244.8 nm; band width: 0.2 nm, flow rate of acetylene and air were 2 and 41 min^{-1}, respectively. The total amount of Pd element on the prepared catalyst was estimated 28 wt%.

Determination of TPEPTA ligand using EDX analysis

The amount of TPEPTA ligand grafted on to GO was calculated through the following equation, using the nitrogen content of TPEPTA from EDX analysis:

$$Amount\ (mol/g) = \frac{\left[\frac{Wt \times 100}{X}\right] \times [\frac{100}{(100 - \frac{Wt \times 100}{X})}}{Y}$$

Where Wt is the weight percent of the element measured, X is the theoretical weight percent of the element in the molecule and Y is the theoretical molecular weight of the molecule. TPEPTA has carbon, hydrogen, and nitrogen contents 51.28, 10.49, and 38.07 wt% with 772.16 g mol^{-1}, respectively. Based on this equation, the amount of TPEPTA ligand detected from the nitrogen content is 1.43 mmol g^{-1} [3].

The extent of TPEPTA ligand per C atoms in graphene layers using TGA analysis

For calculation the extent of functionalization of TPEPTA ligand per C atoms in graphene layeres, weight loss values were employed together with the molecular weight of TPEPTA and the following Equation was used. Where X stands for the number of carbon atoms in the graphene layeres sample per each ligand group, R (%) is the residual mass at 600 °C in the TGA plot, L (%) is the weight loss between 197-239 °C, and Mw is the molecular weight of the TPEPTA ligand. In according the following equation, the extent of the TPEPTA ligand per C atoms in the graphene layers was measured which one ligand per ninety six carbon atoms [4].

$$X=\frac{R\ (\%)\times Mw\ (\frac{g}{mol})}{L\ (\%)\times 12\ (\frac{g}{mol})}$$

Products of Suzuki-Miyaura reaction from aryl chloride at 120 °C (A comparison study with 80 °C)

Table 2S. Synthesis of 8 products that start using aryl chlorides at 120 °C for comparison with the same reactions at 80 °C

Entry	Compound	Time (min)[a]	Yield (%)[a]	Time (min)[b]	Yield (%)[b]
1	**P4**	25	93	25	91
2	**P5**	20	90	20	90
3	**P8**	20	85	25	85
4	**P9**	25	85	25	83
5	**P12**	25	85	25	82
6	**P18**	25	92	20	88
7	**P20**	25	85	30	87
8	**P21**	25	85	30	85

a) Reaction conditions: aryl chloride (1 mmol), phenylboronic acids (1.2 mmol), K_2CO_3 (2 mmol), solvent: DMF: H_2O (2:1) (6 mL), Pd_{np}-$TPEPTA_{(L)}$-GO (30 mg), T: **120 °C**. All yields refer to isolated products. b) Reaction conditions: aryl chloride (1 mmol), phenylboronic acids (1.2 mmol), K_2CO_3 (2 mmol), solvent: DMF: H_2O (2:1) (6 mL), Pd_{np}-$TPEPTA_{(L)}$-GO (30 mg), T: **80 °C**. All yields refer to isolated products.

Synthesis of compound P21

Approach (a): Into a canonical flask (50 mL), a mixture of 2,4-dimethylphenol (6.0 mmol), 4-phenylbenzaldehyde (2.0 mmol), and RGO-SO_3H (40 mg) were stirred under solvent-free conditions at 100 °C for 2.5 h. The progress of the reaction was followed by thin layer

chromatography (TLC) (*n*-hexane: ethyl acetate; 10:4). After completion of the reaction confirmed by TLC, the reaction mixture was cooled down to room temperature and 15 mL of acetone (3×5 mL) was added. The RGO-SO_3H was filtered under reduced pressure using vacuum pump over sinter glass grade 4. The solution was recovered by evaporation on a rotary evaporator. After that, the solid materials were washed with *n*-hexane (5 mL) and deionized water, successively, to afford the pure product **P21**. The desired compound **P21** was kept in an oven at 80 °C for 12 h. (isolated yield: 81%, 661.8 mg).

Approach (b): For the preparation of 6,6′-((4-chloro/bromophenyl)methylene)bis(2,4-dimethylphenol), In a 25 mL round-bottom flask equipped with a magnetic bar and condenser, a mixture of 2,4-dimethylphenol (6.0 mmol), 4-bromo/chlorobenzaldehyde (2.0 mmol) and RGO-SO_3H (40 mg) was heated at 100 °C under solvent free conditions for 2.5 h. The progress of the reaction was monitored by TLC (*n*-hexane: ethyl acetate 10:4). At the end of the reaction, the mixture was cooled to room temperature and 15 mL of acetone (3×5 mL) was added. The RGO-SO_3H was filtered under reduced pressure using vacuum pump over sinter glass grade-4. The solution was recovered by evaporation on a rotary evaporator. After that, the crude compound 6,6′-((4-chloro/bromophenyl)methylene)bis(2,4-dimethylphenol) was washed with *n*-hexane (5 mL) and deionized water, successively, to afford pure product 6,6′-((4-chloro/bromophenyl)methylene)bis(2,4-dimethylphenol). The desired product 6,6′-((4-chloro/bromophenyl)methylene)bis(2,4-dimethylphenol) was kept in an oven at 80 °C for 12 h. Then, 6,6′-((4-chloro/bromophenyl)methylene)bis(2,4-dimethylphenol) (1.0 mmol), phenyl boronic acid (1.2 mmol), Pd_{np}-$TPEPTA_{(L)}$-GO (30 mg), K_2CO_3 (2.0 mmol), and DMF: H_2O (2:1) (6.0 mL) were placed in a 25 mL round-bottom-flask equipped with a condenser and magnetic

stirring bar and heated at 80 °C. The progress of the reaction was monitored using TLC until the 100% conversion of 6,6′-((4-chloro/bromophenyl)methylene)bis(2,4-dimethylphenol) was confirmed. After completion of the reaction, 5.0 mL of hot water and 5.0 mL of ethyl acetate were added to the reaction mixture. The Pd_{np}-$TPEPTA_{(L)}$-GO catalyst was separated under reduced pressure using vacuum pomp over sinter-glass grade-4. The organic solution was evaporated on rotary evaporator and the crude product **P21** was obtained. After that, the solid materials were washed with petroleum ether (5.0 mL) and deionized water, successively, to afford the pure product **P21**. The desired compound **P21** was kept in an oven at 80 °C for 12 h. (X: Br, isolated yield: 91%, 371.8 mg; X: Cl, isolated yield: 86%, 351.3 mg).

Synthesis of compound P22

Compound **P22** was synthesized using different approaches from g to j conditions.

Pathway (g): In a round-bottom flask, 3.3 mmol of 4-aminoacetophenone and 80 mg of RGO-SO_3H were stirred in 5.0 mL of toluene as solvent for 16 h under reflux conditions. When the reaction was completed, the flask was cooled to room temperature using cool water. At this time, the product was solidified and the remained solvent was decanted. Then, the hot methanol or ethyl acetate (10 mL) was added to the flask and the catalyst was filtered over sinter-glass grade 4 using vacuum pump. The solution under the sinter-glass was slowly heated during 2 h (approximately 40 °C). The crude product was recrystallized in ethanol to obtain the pure products. The pure products were dried and stored at 70 °C for 24 h (isolated yield: 62%, 217.8 mg). The RGO-SO_3H was prepared according to the previous work [5].

Pathway (h): The protection of 4-aminoacetophenoe was conducted according to the reported work [6]. After this step, the protected 4-aminoacetophenoe was used in cyclotrimerization

reaction to obtain protected compound **P22** based on previous part. For de-protection step, *tert*-butyl carbamates were cleaved under anhydrous acidic conditions (total yield: 45%, 158.1 mg).

Pathway (i): 1,3,5-tribromobenzene (TBB) (1.0 mmol), 4-aminophenyl boronic acid (1.2 mmol), Pd_{np}-$TPEPTA_{(L)}$-GO (30 mg), K_2CO_3 (2.0 mmol), and DMF: H_2O (2:1) (6.0 mL) were placed in a 25 mL round-bottom-flask equipped with a condenser and magnetic stirring bar and heated at 80 °C for 1 h. The progress of the reaction was monitored using TLC until the 100% conversion of TBB was confirmed. After completion of the reaction, 5.0 mL of hot water and 5.0 mL of ethyl acetate were added to the reaction mixture. The Pd_{np}-$TPEPTA_{(L)}$-GO catalyst was separated under reduced pressure using vacuum pomp over sinter-glass grade-4. The organic solution was evaporated on rotary evaporator and the crude product **P22** was obtained. After that, the solid materials were recrystalized in ethanol, successively, to afford the pure product **P22**. The desired compound **P22** was kept in an oven at 80 °C for 12 h. (Yield: 89%, 312.7 mg).

Pathway (j): 4-bromoaniline (3.3 mmol), 1,3,5-phenyltriboronic acid (1.0 mmol), Pd_{np}-$TPEPTA_{(L)}$-GO (60 mg), K_2CO_3 (2.0 mmol), and DMF: H_2O (2:1) (6.0 mL) were placed in a 25 mL round-bottom-flask equipped with a condenser and magnetic stirring bar and heated at 80 °C for 1.5 h. The progress of the reaction was monitored using TLC until the 100% conversion of 4-bromoaniline was confirmed. After completion of the reaction, 5.0 mL of hot water and 5.0 mL of ethyl acetate were added to the reaction mixture. The Pd_{np}-$TPEPTA_{(L)}$-GO catalyst was separated under reduced pressure using vacuum pomp over sinter-glass grade-4. The organic solution was evaporated on rotary evaporator and the crude product **P22** was obtained. After that, the solid materials were recrystalized in ethanol, successively, to afford the pure product **P22**. The desired compound **P22** was kept in an oven at 80 °C for 12 h. (Yield: 80%, 281.1 mg).

Typically procedures for competing experiments (CE)

Procedure for CE-1: Into a canonical flask (50 mL), a mixture of phenylboronic acid (PBA) (1.2 mmol), 4-methoxyphenylboronic acid (4-OMe-PBA) (1.2 mmol), bromobenzene (1.0 mmol), K_2CO_3 (1.0 mmol), $TPEPTA_{(L)}$-GO (30 mg), and DMF: H_2O (2:1) (6.0 mL) were placed in a 25 mL round-bottom-flask equipped with a condenser and magnetic stirring bar and heated at 80 °C for 1 h. The progress of the reaction was monitored using TLC until the 100% conversion of bromobenzene was confirmed. After completion of the reaction, 5.0 mL of hot water and 5.0 mL of ethyl acetate were added to the reaction mixture. The Pd_{np}-$TPEPTA_{(L)}$-GO catalyst was separated under reduced pressure using vacuum pomp over sinter-glass grade-4. The organic solution was evaporated on rotary evaporator and the crude product **5a** was obtained. The crude product **5a** was purified by column chromatography (*n*-hexane/ethyl acetate) to obtain the desired purity in 75% isolated yield.

Procedure for CE-2: Into a canonical flask (50 mL), PBA (1.2 mmol), 4-OMe-PBA (1.2 mmol), bromobenzene (1.0 mmol), K_2CO_3 (2.0 mmol), $TPEPTA_{(L)}$-GO (30 mg), and DMF: H_2O (2:1) (6.0 mL) were placed in a 25 mL round-bottom-flask equipped with a condenser and magnetic stirring bar and heated at 80 °C for 1 h. The progress of the reaction was monitored using TLC until the 100% conversion of bromobenzene was confirmed. After completion of the reaction, 5.0 mL of hot water and 5.0 mL of ethyl acetate or ethanol were added to the reaction mixture. The Pd_{np}-$TPEPTA_{(L)}$-GO catalyst was separated under reduced pressure using vacuum pomp over sinter-glass grade-4. Into the organic solution, 10.0 mL of CH_2Cl_2 was added. After separation of dichloromethane layer from aqueous layer, the aqueous phases were extracted with dichloromethane (2 × 10 mL) again. The combined organic layers were then dried over anhydrous Na_2SO_4, filtered and concentrated in vacuum to yield the mixture of crude products

6a and **6b**. The crude products **6a** and **6b** were purified by column chromatography (*n*-hexane/ethyl acetate) to obtain the desired purity of **6a** and **6b** in 69% and 20% isolated yield, respectively.

Procedure for CE-3: Into a canonical flask (50 mL), PBA (1.2 mmol), 4-OMe-PBA (1.2 mmol), bromobenzene (1.0 mmol), K_2CO_3 (4.0 mmol), TPEPTA$_{(L)}$-GO (30 mg), and DMF: H_2O (2:1) (6.0 mL) were placed in a 25 mL round-bottom-flask equipped with a condenser and magnetic stirring bar and heated at 80 °C for 1 h. The progress of the reaction was monitored using TLC until the 100% conversion of bromobenzene was confirmed. After completion of the reaction, 5.0 mL of hot water and 5.0 mL of ethyl acetate or ethanol were added to the reaction mixture. The Pd$_{np}$-TPEPTA$_{(L)}$-GO catalyst was separated under reduced pressure using vacuum pomp over sinter-glass grade-4. Into the organic solution, 10.0 mL of CH_2Cl_2 was added. After separation of dichloromethane layer from aqueous layer, the aqueous phases were extracted with dichloromethane (2 × 10 mL) again. The combined organic layers were then dried over anhydrous Na_2SO_4, filtered and concentrated in vacuum to yield the mixture of crude products **7a** and **7b**. The crude products **7a** and **7b** were purified by column chromatography (*n*-hexane/ethyl acetate) to obtain the desired purity of **7a** and **7b** in 61% and 47% isolated yield, respectively.

Procedure for CE-4: Into a canonical flask (50 mL), 4-bromobenzaldehyde (1.0 mmol), 3-bromobenzaldehyde (1.0 mmol), PBA (1.2 mmol), K_2CO_3 (2.0 mmol), TPEPTA$_{(L)}$-GO (30 mg), and DMF: H_2O (2:1) (6.0 mL) were placed in a 25 mL round-bottom-flask equipped with a condenser and magnetic stirring bar and heated at 80 °C for 1 h. The progress of the reaction was monitored using TLC until the highly conversion of 3-bromobenzaldehyde and 4-bromobenzaldehyde was confirmed. After completion of the reaction, 5.0 mL of hot water and

5.0 mL of ethyl acetate or ethanol were added to the reaction mixture. The Pd_{np}-$TPEPTA_{(L)}$-GO catalyst was separated under reduced pressure using vacuum pomp over sinter-glass grade-4. Into the organic solution, 10.0 mL of CH_2Cl_2 was added. After separation of dichloromethane layer from aqueous layer, the aqueous phases were extracted with dichloromethane (2 × 10 mL) again. The combined organic layers were then dried over anhydrous Na_2SO_4, filtered and concentrated in vacuum to yield the mixture of crude products **8a** and **8b**. The crude products **8a** and **8b** were purified by column chromatography (*n*-hexane/ethyl acetate) to obtain the desired purity of **8a** and **8b** in 70% and 15% isolated yield, respectively.

Procedure for CE-5: Into a canonical flask (50 mL), 4-bromobenzaldehyde (1.0 mmol), 3-bromobenzaldehyde (1.0 mmol), 4-OMe-PBA (1.2 mmol), K_2CO_3 (2.0 mmol), $TPEPTA_{(L)}$-GO (30 mg), and DMF: H_2O (2:1) (6.0 mL) were placed in a 25 mL round-bottom-flask equipped with a condenser and magnetic stirring bar and heated at 80 °C for 1 h. The progress of the reaction was monitored using TLC until the highly conversion of 3-bromobenzaldehyde and 4-bromobenzaldehyde was confirmed. After completion of the reaction, 5.0 mL of hot water and 5.0 mL of ethyl acetate or ethanol were added to the reaction mixture. The Pd_{np}-$TPEPTA_{(L)}$-GO catalyst was separated under reduced pressure using vacuum pomp over sinter-glass grade-4. The organic solution was evaporated on rotary evaporator and the crude product **9a** was obtained. The crude product **9a** was purified by column chromatography (*n*-hexane/ethyl acetate) to obtain the desired purity in 82% isolated yield.

Procedure for CE-6: A mixture of 4-bromobenzaldehyde (1.0 mmol), 4-bromoanisole (1.0 mmol), 4-OMe-PBA (1.2 mmol), K_2CO_3 (2.0 mmol), $TPEPTA_{(L)}$-GO (30 mg), and DMF: H_2O (2:1) (6.0 mL) were placed in a 25 mL round-bottom-flask equipped with a condenser and

magnetic stirring bar and heated at 80 °C for 1 h. The progress of the reaction was monitored using TLC until the highly conversion of starting compounds was confirmed. After completion of the reaction, 5.0 mL of hot water and 5.0 mL of ethyl acetate were added to the reaction mixture. The Pd_{np}-$TPEPTA_{(L)}$-GO catalyst was separated under reduced pressure using vacuum pomp over sinter-glass grade-4. The organic solution was evaporated on rotary evaporator and the crude product **5a** was obtained. The crude product **10a** was purified by column chromatography (*n*-hexane/ethyl acetate) to obtain the desired purity of **10a** in 89% isolated yield.

Characterization of reused Pd_{np}-$TPEPTA_{(L)}$-GO catalyst

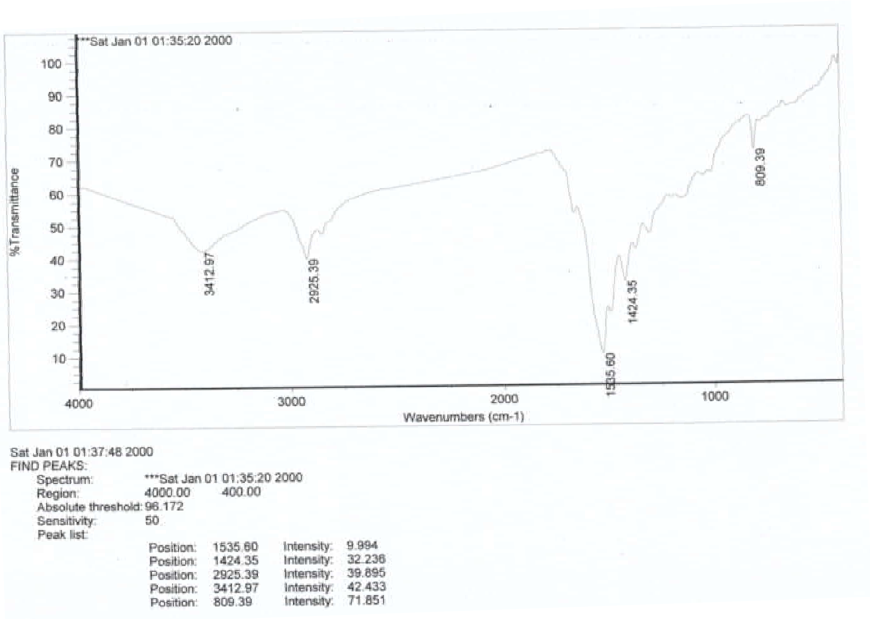

Figure 1S, FT-IR spectrum of reused Pd_{np}-$TPEPTA_{(L)}$-GO catalyst (6th)

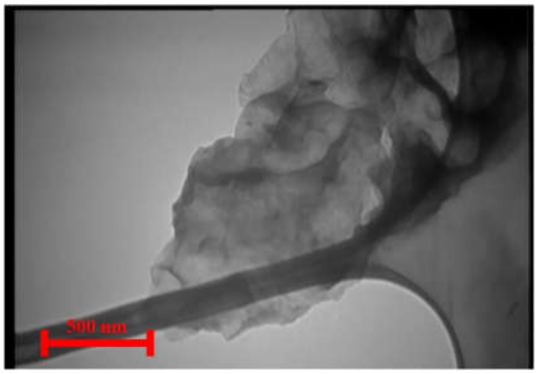

Figure 2S, TEM image of reused Pd_{np}-TPEPTA$_{(L)}$-GO catalyst (6th)

The synthetic routes for the preparation of Pd_{np}-TPEPTA$_{(L)}$-GO catalyst

Synthesis of Pd_{np}-TPEPTA$_{(L)}$-GO catalyst

The Pd_{np}-TPEPTA$_{(L)}$-GO catalyst was prepared according to the following synthetic route shown in **Scheme 2** in the main text of the manuscript.

Step 1: Synthesis of TPEPTA ligand

Into a two-neck round-bottom flask equipped with magnet and condenser, a mixture of TCT (9.0 mmol, 1.66 g), PEHA (30 mmol, 6.96 g), and Et_3N (0.1 mL) were added in dry DMF (25 mL) as solvent at 80 °C for 12 h under N_2 atmosphere. After completion of the reaction, the reaction mixture was cooled down to room temperature and was filtered and washed with aqueous $NaHCO_3$ 10% and warm deionized water. It is noteworthy that washing the TPEPTA with warm deionized water is necessary until all unreacted PEHA and Et_3N are eliminated. After drying in the oven at 80 °C for 24 h, the TPEPTA ligand was obtained as a dark red-orange powder in 68% isolated yield (4.7 g).

Step 2: Preparation of Graphene oxide and functionalization with 3 chloropropyltrimethoxysilane

Graphene oxide was produced from raw graphite powder (Merck Company) regarding our previous work [5]. First, sodium nitrate ($NaNO_3$) (2.5 g) and natural graphite powder (5.0 g) were mixed with H_2SO_4 (98%) (115 mL) in a 1000 mL round-bottom flask equipped with a magnetic stirrer and condenser place in an ice won at 0-5 °C. The mixture was stirred and potassium permanganate ($KMnO_4$) (15.0 g) was slowly added during 1 h, and the stirring of mixture was followed for 2 h. The mixture was transferred to water won (35 ^{o} C) and stirred for

30 min. Then, distilled water (230 mL) was slowly added into the mixture and the temperature of mixture was about 98 °C and stirred for 15 min. Then, distilled water (700 mL) and hydrogen peroxide (H_2O_2) (30%) (50 mL) were sequentially added to the mixture until the reaction was concluded. The final materials were filtered and eluted exhaustively with hydrogen chloride (5%) and distilled water for several times. The solution was filtered under reduced pressure by vacuum pump upon sinter-glass (G4). The graphite oxide powder was obtained after drying in vacuum oven at 60 °C for 12 h. The graphite oxide was dispersed in distilled water to make concentration of 0.5 mg mL^{-1}, and exfoliated by ultrasound (40 W) for 30 min, followed by centrifugation at 3500 rpm for 30 min to delete un-exfoliated graphite oxide.

After preparation of graphene oxide, Into a two-neck round-bottom flask equipped with magnet and condenser under N_2 atmosphere, a mixture graphene oxide (1.0 g) and 3-chloropropyltrimethoxysilane (5.0 mL) were added in dry toluene (25 mL) as solvent for 6 h under reflux conditions. After completion of the reaction, the reaction mixture was cooled down to room temperature and was filtered over sinter glass grade-4 and washed with dry toluene. The functionalized graphene oxide with 3-chloropropyltrimethoxysilane was stored in desiccator under N_2 atmosphere.

Step 3: synthesis of Pd_{np}-TPEPTA$_{(L)}$-GO catalyst

Into a two-neck round-bottom flask equipped (250 mL) with magnet and condenser under N_2 atmosphere, a mixture of TPEPTA ligand (3.0 mmol, 2.3 g), 3-chloropropylsilyl supported on graphene oxide (1.0 g), and Et_3N (0.2 mL) in dry DMF (50 mL), was added and stirred for 12 h at 80 °C. Then, the mixture was filtered and washed with aqueous $NaHCO_3$ 10%, hot deionized water, and 10 mL of DMF (2 × 5 mL). After drying in a vacuum oven at 120 °C overnight, the

TPEPTA(L)-GO was obtained as a dark solid. At the final step, into a two-neck round-bottom flask equipped (100 mL) with magnet and condenser under N_2 atmosphere, TPEPTA(L)-GO (2.0 g), $Pd(OAc)_2$ (0.2 g, 0.9 mmol), and SDS (3.0 mmol) in absolute ethanol (25 mL) at 40 °C for 24 h was added and stirred. After the appropriate time, the dark solid Pd_{np}-TPEPTA(L)-GO catalyst was filtered and washed with deionized water and absolute ethanol and dried at 100 °C overnight.

Spectroscopic and Physical data of the synthesized compounds

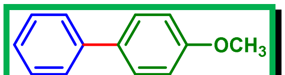

P1

4-Methoxy-1,1′-biphenyl (Scheme 3, compound P1)
M.p.: 87-89 °C; CAS number: (613-37-6)
R_f(*n*-hexane/EtOAc 18:2): 0.62

To a solution of 4-iodoanisole or 4-bromoanisole (1.0 mmol), phenyl boronic acid (1.2 mmol, 0.146 g), Pd_{np}-TPEPTA(L)-GO (30 mg), K_2CO_3 (2.0 mmol, 0.276 g), and DMF: H_2O (2:1) (6.0 mL) were placed in a 25 mL round-bottom-flask equipped with a condenser and magnetic stirring bar and heated at 80 °C. The progress of the reaction was monitored using TLC. After completion of the reaction, 5.0 mL of hot water and 5.0 mL of ethyl acetate were added to the reaction mixture. The Pd_{np}-TPEPTA(L)-GO catalyst was separated under reduced pressure using vacuum pomp over sinter-glass grade-4. The organic solution was evaporated on rotary evaporator and the crude compound P1 was obtained. The crude product was purified by column chromatography, eluting with hexane/EtOAc, to afford a pure product as a white solid powder. (X: I; isolated yield after column chromatography: 95%, 175 mg), (X: Br; isolated yield after column chromatography: 95%, 175 mg).

IR (KBr), ν (cm^{-1}): 3060, 3055, 2959, 1605, 1485, 1249, 1036. ^1H NMR (400 MHz, $CDCl_3$): δ (ppm) 3.85 (3H, s), 6.98 (2H, d, *J* = 8.4 Hz), 7.30 (1H, t, *J* = 7.2 Hz), 7.42 (2H, t, *J* = 7.6 Hz), 7.55 (4H, t, *J* = 7.6 Hz). Anal. Calcd for $C_{13}H_{12}O$: C, 84.75; H, 6.57. Found: C, 83.56; H, 5.86.

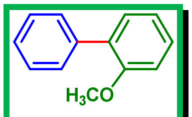

P2

2-Methoxy-1,1′-biphenyl (Scheme 3, compound P2)
M.p.: oil; CAS number: (86-26-0)
R_f(*n*-hexane/EtOAc 18:2): 0.51

To a solution of 2-iodoanisole or 2-bromoanisole (1.0 mmol), phenyl boronic acid (1.2 mmol, 0.146 g), Pd_{np}-TPEPTA(L)-GO (30 mg), K_2CO_3 (2.0 mmol, 0.276 g), and DMF: H_2O (2:1) (6.0 mL) were placed in a 25 mL round-bottom-flask equipped with a condenser and magnetic stirring bar and heated at 80 °C. The progress of the reaction was monitored using TLC. After completion of the reaction, 5.0 mL of hot water and 5.0 mL of ethyl acetate were added to the reaction mixture. The Pd_{np}-TPEPTA(L)-GO catalyst was separated under reduced pressure using vacuum pomp over sinter-glass grade-4. The organic solution was evaporated on rotary evaporator and the crude compound P2 was obtained. The crude product was purified by column chromatography, eluting with hexane/EtOAc, to afford a pure product of compound P2. (X: I; isolated yield after column chromatography: 97%,

178 mg), (X: Br; isolated yield after column chromatography: 95%, 175 mg).

IR (KBr), ν (cm^{-1}): 3059, 2932, 1597, 1481, 1259, 1028. ^1H NMR (400 MHz, $CDCl_3$): δ (ppm) 3.78 (3H, s), 6.98 (1H, d, J = 8.8 Hz), 7.02 (1H, t, J = 8.8 Hz), 7.28-7.33 (3H, m), 7.40 (2H, t, J = 7.2 Hz), 7.53 (2H, d, J = 5.2 Hz). Anal. Calcd for $C_{13}H_{12}O$: C, 84.75; H, 6.57. Found: C, 82.95; H, 5.66.

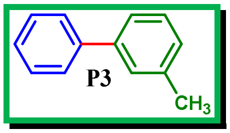

3-Methyl-1,1′-biphenyl (Scheme 3, compound P3)
M.p.: 42-44 °C; CAS number: (643-93-6)
R_f(*n*-hexane/EtOAc 18:2): 0.46

To a solution of 1-bromo-3-methylbenzene (1.0 mmol, 0.171 g), phenyl boronic acid (1.2 mmol, 0.146 g), Pd_{np}-$TPEPTA_{(L)}$-GO (30 mg), K_2CO_3 (2.0 mmol, 0.276 g), and DMF: H_2O (2:1) (6.0 mL) were placed in a 25 mL round-bottom-flask equipped with a condenser and magnetic stirring bar and heated at 80 °C. The progress of the reaction was monitored using TLC. After completion of the reaction, 5.0 mL of hot water and 5.0 mL of ethyl acetate were added to the reaction mixture. The Pd_{np}-$TPEPTA_{(L)}$-GO catalyst was separated under reduced pressure using vacuum pomp over sinter-glass grade-4. The organic solution was evaporated on rotary evaporator and the crude compound P3 was obtained. The crude product was purified by column chromatography, eluting with hexane/EtOAc, to afford a pure product of compound P3. (X: Br; isolated yield after column chromatography: 93%, 156 mg).

IR (KBr): ν (cm^{-1}) 3064, 2922, 1588, 1476, 749, 699. ^1H NMR (400 MHz, $CDCl_3$) δ (ppm): 2.41 (3H, s, CH_3), 7.18 (1H, d, *J=7.6 Hz,* phenyl), 7.41-7.49 (5H, m, phenyl), 7.73-7.76 (3H, m, phenyl).

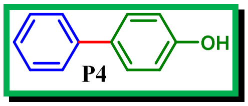

4-Phenylphenol (Scheme 3, compound P4)
M.p.: 156-159 °C; CAS number: (92-69-3)
R_f(*n*-hexane/EtOAc 18:2): 0.66

To a solution of 4-iodophenol or 4-bromophenol or 4-chlorophenol (1.0 mmol), phenyl boronic acid (1.2 mmol, 0.146 g), Pd_{np}-$TPEPTA_{(L)}$-GO (30 mg), K_2CO_3 (2.0 mmol, 0.276 g), and DMF: H_2O (2:1) (6.0 mL) were placed in a 25 mL round-bottom-flask equipped with a condenser and magnetic stirring bar and heated at 80 °C. The progress of the reaction was monitored using TLC. After completion of the reaction, 5.0 mL of hot water and 5.0 mL of ethyl acetate or ethanol were added to the reaction mixture. The Pd_{np}-$TPEPTA_{(L)}$-GO catalyst was separated under reduced pressure using vacuum pomp over sinter-glass grade-4. The organic solution was evaporated on rotary evaporator and the crude compound P4 was obtained. The crude product was purified by column chromatography, eluting with hexane/EtOAc, to afford a pure product of compound P4. (X: I; isolated yield after column chromatography: 96%, 163 mg), (X: Br; isolated yield after column chromatography: 96%, 163 mg), (X: Cl; isolated yield after column chromatography: 91%, 154 mg).

IR (KBr), ν (cm^{-1}): 3406, 3036, 1603, 1486. ^1H NMR (400 MHz, $CDCl_3$): δ (ppm) 6.93 (2H, d, J = 8.4 Hz), 7.33 (1H, t, J = 7.2 Hz), 7.44 (2H, t, J = 7.6 Hz), 7.51 (2H, d, J = 8.4 Hz), 7.56 (2H, t, J = 7.2 Hz), 8.28 (1H, d, J = 6.8 Hz). Anal. Calcd for $C_{12}H_{10}O$: C, 84.68; H, 5.92. Found: C, 83.95; H, 5.66.

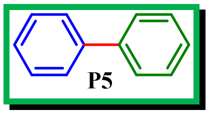

1,1′-Biphenyl (Scheme 3, compound P5)
M.p.: 68-69 °C; CAS number: (92-52-4)
R_f(*n*-hexane/EtOAc 18:2): 0.35

To a solution of 4-iodobenzene or 4-bromobenzene or 4-chlorobenzene (1.0 mmol), phenyl boronic acid (1.2 mmol, 0.146 g), Pd$_{np}$-TPEPTA$_{(L)}$-GO (30 mg), K_2CO_3 (2.0 mmol, 0.276 g), and DMF: H_2O (2:1) (6.0 mL) were placed in a 25 mL round-bottom-flask equipped with a condenser and magnetic stirring bar and heated at 80 °C. The progress of the reaction was monitored using TLC. After completion of the reaction, 5.0 mL of hot water and 5.0 mL of ethyl acetate were added to the reaction mixture. The Pd$_{np}$-TPEPTA$_{(L)}$-GO catalyst was separated under reduced pressure using vacuum pomp over sinter-glass grade-4. The organic solution was evaporated on rotary evaporator and the crude compound P5 was obtained. The crude product was purified by column chromatography, eluting with hexane/EtOAc, to afford a pure product of compound P5. (X: I; isolated yield after column chromatography: 93%, 143 mg), (X: Br; isolated yield after column chromatography: 93%, 143 mg), (X: Cl; isolated yield after column chromatography: 90%, 138 mg).

IR (KBr), ν (cm^{-1}): 3033, 1567, 1477. ^{1}H NMR (400 MHz, $CDCl_3$): δ (ppm) 7.33-7.38 (2H, m), 7.45 (4H, t, J = 7.2 Hz), 7.61 (4H, d, J = 8 Hz). Anal. Calcd for $C_{12}H_{10}$: C, 93.46; H, 6.54. Found: C, 92.75; H, 5.86.

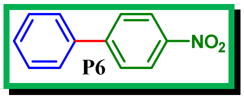

4-Nitro-1,1′-biphenyl (Scheme 3, compound P6)
M.p.: 107-109 °C; CAS number: (92-93-3)
R$_f$ (*n*-hexane/EtOAc 18:2): 0.67

To a solution of 1-bromo-4-nitrobenzene (1.0 mmol, 0.202 g), phenyl boronic acid (1.2 mmol, 0.146 g), Pd$_{np}$-TPEPTA$_{(L)}$-GO (30 mg), K_2CO_3 (2.0 mmol, 0.276 g), and DMF: H_2O (2:1) (6.0 mL) were placed in a 25 mL round-bottom-flask equipped with a condenser and magnetic stirring bar and heated at 80 °C. The progress of the reaction was monitored using TLC. After completion of the reaction, 5.0 mL of hot water and 5.0 mL of ethyl acetate were added to the reaction mixture. The Pd$_{np}$-TPEPTA$_{(L)}$-GO catalyst was separated under reduced pressure using vacuum pomp over sinter-glass grade-4. The organic solution was evaporated on rotary evaporator and the crude compound P6 was obtained. The crude product was purified by column chromatography, eluting with hexane/EtOAc, to afford a pure product of compound P6. (X: Br; isolated yield after column chromatography: 89%, 177 mg).

IR (KBr), ν (cm^{-1}): 3098, 1597, 1513, 1476, 1344. ^{1}H NMR (400 MHz, $CDCl_3$): δ (ppm) 7.46-7.53 (3H, m), 7.64 (2H, d, J = 6.8 Hz), 7.75 (2H, d, J = 8.8 Hz), 8.31 (2H, d, J = 6.8 Hz). Anal. Calcd for $C_{12}H_9NO_2$: C, 72.35; H, 4.55; N, 7.03. Found: C, 71.18; H, 4.66; N, 6.44.

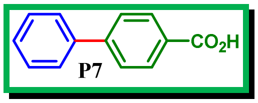

4-Phenylbenzene carboxylic acid (Scheme 3, compound P7)
M.p.: 217-219 °C; CAS number: (92-92-2)
R$_f$ (*n*-hexane/EtOAc 18:2): 0.49

To a solution of 4-bromobenzoic acid (1.0 mmol, 0.201 g), phenyl boronic acid (1.2 mmol, 0.146 g), Pd$_{np}$-TPEPTA$_{(L)}$-GO (30 mg), K_2CO_3 (2.0 mmol, 0.276 g), and DMF: H_2O (2:1) (6.0 mL) were placed in a 25 mL round-bottom-flask equipped with a condenser and magnetic stirring bar and heated at 80 °C. The progress of the reaction was monitored using TLC. After completion of the reaction, 5.0 mL of hot water and 5.0 mL of ethyl acetate or ethanol were added to the reaction mixture. The Pd$_{np}$-TPEPTA$_{(L)}$-GO catalyst was separated under reduced pressure using vacuum pomp over sinter-glass grade-4. The organic solution was evaporated on rotary evaporator and the crude compound P7 was obtained. The crude product was purified by column chromatography, eluting with hexane/EtOAc, to afford a pure product of compound P7. (X: Br; isolated yield after column chromatography: 88%, 174 mg).

IR (KBr), ν (cm^{-1}): 3431, 1679, 1603, 1442, 1306. ^{1}H NMR (400 MHz, $CDCl_3$): δ (ppm) 7.32 (1H, t, J = 7.4 Hz), 7.40 (2H, dd, J = 7.3 Hz), 7.55 (2H, d, J = 6.9 Hz), 7.71 (2H, d, J = 8.2 Hz), 8.15 (2H, d, J = 7.5 Hz). Anal. Calcd for $C_{13}H_{10}O_2$: C, 78.77; H, 5.09; Found: C, 77.67; H, 4.36.

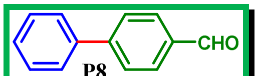

P8

4-Phenylbenzaldehyde (Scheme 3, compound P8)
M.p.: 56-58 °C; CAS number: (3218-36-8)
R_f(*n*-hexane/EtOAc 18:2): 0.71

To a solution of 4-iodobenzaldehyde or 4-bromobenzaldehyde or 4-chlorobenzaldehyde (1.0 mmol), phenyl boronic acid (1.2 mmol, 0.146 g), Pd_{np}-$TPEPTA_{(L)}$-GO (30 mg), K_2CO_3 (2.0 mmol, 0.276 g), and DMF: H_2O (2:1) (6.0 mL) were placed in a 25 mL round-bottom-flask equipped with a condenser and magnetic stirring bar and heated at 80 °C. The progress of the reaction was monitored using TLC. After completion of the reaction, 5.0 mL of hot water and 5.0 mL of ethyl acetate or ethanol were added to the reaction mixture. The Pd_{np}-$TPEPTA_{(L)}$-GO catalyst was separated under reduced pressure using vacuum pomp over sinter-glass grade-4. The organic solution was evaporated on rotary evaporator and the crude compound P8 was obtained. The crude product was purified by column chromatography, eluting with hexane/EtOAc, to afford a pure product of compound P8. (X: I; isolated yield after column chromatography: 89%, 162 mg), (X: Br; isolated yield after column chromatography: 86%, 156 mg), (X: Cl; isolated yield after column chromatography: 85%, 154 mg).

IR (KBr), ν (cm^{-1}): 3066, 1710, 1588, 1476, 765, 691. ^1H NMR (400 MHz, $CDCl_3$) δ (ppm): 7.45 (1H, t, *J= 6.8 Hz*), 7.53 (2H, t, *J= 7.1 Hz*), 7.77 (2H, d, *J= 7.8 Hz*), 7.91 (2H, d, *J= 7.8 Hz*), 7.99 (2H, d, *J= 8.2 Hz*), 10.06 (1H, s, CHO). Anal. Calcd for $C_{13}H_{10}O$: C, 85.69; H, 5.53; Found: C, 84.67; H, 4.76.

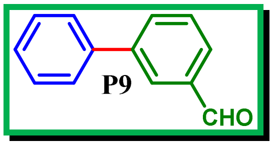

P9

3-Phenylbenzaldehyde (Scheme 3, compound P9)
M.p.: 52-53 °C; CAS number: (1204-60-0)
R_f(*n*-hexane/EtOAc 18:2): 0.68

To a solution of 4-bromobenzaldehyde or 4-chlorobenzaldehyde (1.0 mmol), phenyl boronic acid (1.2 mmol, 0.146 g), Pd_{np}-$TPEPTA_{(L)}$-GO (30 mg), K_2CO_3 (2.0 mmol, 0.276 g), and DMF: H_2O (2:1) (6.0 mL) were placed in a 25 mL round-bottom-flask equipped with a condenser and magnetic stirring bar and heated at 80 °C. The progress of the reaction was monitored using TLC. After completion of the reaction, 5.0 mL of hot water and 5.0 mL of ethyl acetate or ethanol were added to the reaction mixture. The Pd_{np}-$TPEPTA_{(L)}$-GO catalyst was separated under reduced pressure using vacuum pomp over sinter-glass grade-4. The organic solution was evaporated on rotary evaporator and the crude compound P9 was obtained. The crude product was purified by column chromatography, eluting with hexane/EtOAc, to afford a pure product of compound P9. (X: Br; isolated yield after column chromatography: 85%, 154 mg), (X: Cl; isolated yield after column chromatography: 83%, 151 mg).

IR (KBr), ν (cm^{-1}): 3060, 2826, 2728, 1697, 1594, 1476. ^1H NMR (400 MHz, $CDCl_3$): δ (ppm) 7.41-7.45 (1H, m), 7.48-7.52 (2H, m), 7.63 (2H, d, *J* = 7.2 Hz), 7.66 (1H, t, *J* = 1.6 Hz), 7.87-7.89 (2H, dd, *J* = 6.4 Hz), 8.13 (1H, s), 10.11 (1H, s). Anal. Calcd for $C_{13}H_{10}O$: C, 85.69; H, 5.53; Found: C, 85.17; H, 4.96.

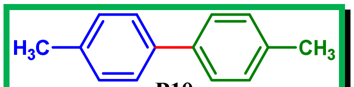

P10

4,4'-Dimethyl-1,1'-biphenyl (Scheme 3, compound P10)
M.p.: 118-120 °C; CAS number: (613-33-2)
R_f(*n*-hexane/EtOAc 18:2): 0.34

To a solution of 1-iodo-4-methylbenzene or 1-bromo-4-methylbenzene (1.0 mmol), 4-methylphenyl boronic acid (1.2 mmol, 0.163 g), Pd_{np}-$TPEPTA_{(L)}$-GO (30 mg), K_2CO_3 (2.0 mmol, 0.276 g), and DMF: H_2O (2:1) (6.0 mL) were placed in a 25 mL round-bottom-flask equipped with a condenser and magnetic stirring bar and heated at 80 °C. The progress of the reaction was monitored using TLC. After completion of the reaction, 5.0 mL of hot water and 5.0

mL of ethyl acetate or ethanol were added to the reaction mixture. The Pd_{np}-$TPEPTA_{(L)}$-GO catalyst was separated under reduced pressure using vacuum pomp over sinter-glass grade-4. The organic solution was evaporated on rotary evaporator and the crude compound P10 was obtained. The crude product was purified by column chromatography, eluting with hexane/EtOAc, to afford a pure product of compound P10. (X: I; isolated yield after column chromatography: 96%, 175 mg), (X: Br; isolated yield after column chromatography: 95%, 173 mg).

IR (KBr): ν (cm^{-1}) 3062, 2932, 1585, 1476, 746, 699. ^1H NMR (250 MHz, $CDCl_3$): δ (ppm) = 2.33 (s, 6H), 7.12 (d, J = 6.2 Hz, 4H), 7.40 (d, J = 6.2 Hz, 4H). ^{13}CNMR (62.5 MHz, $CDCl_3$): δ (ppm) = 21.1, 126.8, 129.4, 136.9, 138.3.

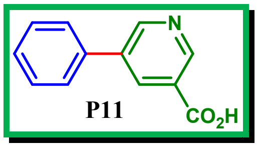

5-Phenylnicotinic acid (Scheme 3, compound P11)
M.p.: 252-254 °C; CAS number: (10177-12-5)
R_f (*n*-hexane/EtOAc 18:2): 0.71

To a solution of 5-bromopyridine-3-carboxylic acid (1.0 mmol, 0.202 g), phenyl boronic acid (1.2 mmol, 0.146 g), Pd_{np}-$TPEPTA_{(L)}$-GO (30 mg), K_2CO_3 (2.0 mmol, 0.276 g), and DMF: H_2O (2:1) (6.0 mL) were placed in a 25 mL round-bottom-flask equipped with a condenser and magnetic stirring bar and heated at 80 °C. The progress of the reaction was monitored using TLC. After completion of the reaction, 10.0 mL of hot water and 5.0 mL of ethyl acetate or acetonitrile were added to the reaction mixture. The Pd_{np}-$TPEPTA_{(L)}$-GO catalyst was separated under reduced pressure using vacuum pomp over sinter-glass grade-4. The organic solution was evaporated on rotary evaporator and the crude compound P11 was obtained. The crude product was purified by column chromatography, eluting with hexane/EtOAc, and then recrystalized from ethanol, to afford a pure product of compound P11. (X: Br; isolated yield after column chromatography: 82%, 163 mg).

IR (KBr), ν (cm^{-1}): 3431, 3076, 1676, 1603, 1442. ^1H NMR (400 MHz, $CDCl_3$): δ (ppm) 7.51 (3H, t, J = 7.6 Hz), 7.59 (2H, t, J = 7.6 Hz), 7.69 (1H, s), 8.23 (1H, s), 8.25 (1H, s). Anal. Calcd for $C_{12}H_9NO_2$: C, 72.35; H, 4.55; N, 7.03. Found: C, 71.97; H, 4.36; N, 6.98.

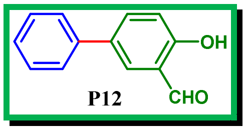

2-Hydroxy-5-phenylbenzaldehyde (Scheme 3, compound P12)
M.p.: 96-99 °C; CAS number: (1761-63-3)
R_f (*n*-hexane/EtOAc 18:2): 0.56

To a solution of 5-bromo-2-hydroxybenzaldehyde or 5-chloro-2-hydroxybenzaldehyde (1.0 mmol), phenyl boronic acid (1.2 mmol, 0.146 g), Pd_{np}-$TPEPTA_{(L)}$-GO (30 mg), K_2CO_3 (2.0 mmol, 0.276 g), and DMF: H_2O (2:1) (6.0 mL) were placed in a 25 mL round-bottom-flask equipped with a condenser and magnetic stirring bar and heated at 80 °C. The progress of the reaction was monitored using TLC. After completion of the reaction, 10.0 mL of hot water and 5.0 mL of ethyl acetate were added to the reaction mixture. The Pd_{np}-$TPEPTA_{(L)}$-GO catalyst was separated under reduced pressure using vacuum pomp over sinter-glass grade-4. The organic solution was evaporated on rotary evaporator and the crude compound P12 was obtained. The crude product was purified by column chromatography, eluting with hexane/EtOAc, and then recrystalized from ethanol, to afford a pure product of compound P12. (X: Br; isolated yield after column chromatography: 89%, 176 mg), (X: Cl; isolated yield after column chromatography: 82%, 162 mg).

IR (KBr), ν (cm^{-1}): 3419, 3043, 2856, 1673, 1603, 1465. ^1H NMR (400 MHz, $CDCl_3$): δ (ppm) 6.94 (1H, d, J = 8.8 Hz), 7.54 (2H, t, J = 7.6 Hz), 7.63 (2H, m), 7.70 (1H, d, J = 2.8 Hz), 8.26 (1H, d, J = 1.2 Hz), 8.29 (1H, d, J = 1.2 Hz), 10.96 (1H, s). Anal. Calcd for $C_{13}H_{10}O_2$: C, 78.77; H, 5.09. Found: C, 77.97; H, 4.76.

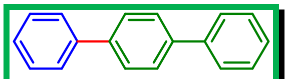

P13

1,4-Diphenylbenzene (Scheme 3, compound P13)
M.p.: 189-192 °C; CAS number: (92-94-4)
R_f (*n*-hexane/EtOAc 18:2): 0.42

To a solution of 4-bromobiphenyl (1.0 mmol, 0.233 g), phenyl boronic acid (1.2 mmol, 0.146 g), Pd_{np}-$TPEPTA_{(L)}$-GO (30 mg), K_2CO_3 (2.0 mmol, 0.276 g), and DMF: H_2O (2:1) (6.0 mL) were placed in a 25 mL round-bottom-flask equipped with a condenser and magnetic stirring bar and heated at 80 °C. The progress of the reaction was monitored using TLC. After completion of the reaction, 5.0 mL of hot water and 5.0 mL of ethyl acetate or ethanol were added to the reaction mixture. The Pd_{np}-$TPEPTA_{(L)}$-GO catalyst was separated under reduced pressure using vacuum pomp over sinter-glass grade-4. The organic solution was evaporated on rotary evaporator and the crude compound P13 was obtained. The crude product was purified by column chromatography, eluting with hexane/EtOAc, to afford a pure product of compound P13. (X: Br; isolated yield after column chromatography: 90%, 207 mg).

IR (KBr), ν (cm^{-1}): 3030, 1621, 1461. ^1H NMR (400 MHz, $CDCl_3$): δ (ppm) 7.27-7.31 (2H, m), 7.39 (4H, t, *J* = 7.6 Hz), 7.57 (4H, d, *J* = 7.2 Hz), 7.61 (4H, s). Anal. Calcd for $C_{18}H_{14}$: C, 93.87; H, 6.13. Found: C, 92.97; H, 5.36.

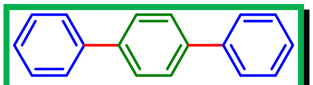

P14

1,4-Diphenylbenzene (Scheme 3, compound P14)
M.p.: 188-190 °C; CAS number: (92-94-4)
R_f (*n*-hexane/EtOAc 18:2): 0.42

To a solution of 1,4-dibromobenzene (1.0 mmol, 0.235 g), phenyl boronic acid (2.4 mmol, 0.292 g), Pd_{np}-$TPEPTA_{(L)}$-GO (30 mg), K_2CO_3 (4.0 mmol, 0.552 g), and DMF: H_2O (2:1) (6.0 mL) were placed in a 25 mL round-bottom-flask equipped with a condenser and magnetic stirring bar and heated at 80 °C. The progress of the reaction was monitored using TLC. After completion of the reaction, 5.0 mL of hot water and 5.0 mL of ethyl acetate or ethanol were added to the reaction mixture. The Pd_{np}-$TPEPTA_{(L)}$-GO catalyst was separated under reduced pressure using vacuum pomp over sinter-glass grade-4. The organic solution was evaporated on rotary evaporator and the crude compound P14 was obtained. The crude product was purified by column chromatography, eluting with hexane/EtOAc, to afford a pure product of compound P14. (X: Br; isolated yield after column chromatography: 95%, 218 mg).

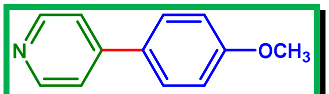

P15

4-(4-methoxyphenyl)pyridine (Scheme 3, compound P15)
M.p.: 89-91 °C; CAS number: (5938-16-9)
R_f (*n*-hexane/EtOAc 18:2): 0.63

To a solution of 4-bromopyridine (1.0 mmol, 0.157 g), 4-methoxyphenyl boronic acid (1.2 mmol, 0.182 g), Pd_{np}-$TPEPTA_{(L)}$-GO (30 mg), K_2CO_3 (2.0 mmol, 0.276 g), and DMF: H_2O (2:1) (6.0 mL) were placed in a 25 mL round-bottom-flask equipped with a condenser and magnetic stirring bar and heated at 80 °C. The progress of the reaction was monitored using TLC. After completion of the reaction, 5.0 mL of hot water and 5.0 mL of ethyl acetate or ethanol were added to the reaction mixture. The Pd_{np}-$TPEPTA_{(L)}$-GO catalyst was separated under reduced pressure using vacuum pomp over sinter-glass grade-4. The organic solution was evaporated on rotary evaporator and the crude compound P15 was obtained. The crude product was purified by column chromatography, eluting with

hexane/EtOAc, and recrystalized from ethanol/n-hexane (1:10) to afford a pure product of compound P15. (X: Br; isolated yield after column chromatography: 86%, 159 mg).

^{1}H NMR (250 MHz, $CDCl_3$): δ (ppm) = 3.87 (s, 3H), 7.00 (d, J = 8.3 Hz, 2H), 7.50 (d, J = 5.4 Hz, 2H), 7.62 (d, J = 8.3 Hz, 2H), 8.62 (d, J = 5.4 Hz, 2H). ^{13}C NMR (62.5 MHz, $CDCl_3$): δ (ppm) = 55.4, 114.5, 121.2, 128.0, 130.4, 147.8, 150.1, 150.7, 160.8.

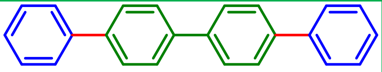

P16

Quaterpheny (Scheme 3, compound P16)
M.p.: 298-300 °C; CAS number (135-70-6)
R_f (n-hexane/EtOAc 18:2): 0.30

To a solution of 1,4-dibromobenzene (1.0 mmol, 0.235 g), phenyl boronic acid (2.4 mmol, 0.292 g), Pd_{np}-$TPEPTA_{(L)}$-GO (30 mg), K_2CO_3 (2.0 mmol, 0.276 g), and DMF: H_2O (2:1) (6.0 mL) were placed in a 25 mL round-bottom-flask equipped with a condenser and magnetic stirring bar and heated at 80 °C. The progress of the reaction was monitored using TLC. After completion of the reaction, 5.0 mL of hot water and 5.0 mL of ethyl acetate or ethanol were added to the reaction mixture. The Pd_{np}-$TPEPTA_{(L)}$-GO catalyst was separated under reduced pressure using vacuum pomp over sinter-glass grade-4. The organic solution was evaporated on rotary evaporator and the crude compound P16 was obtained. The crude product was purified by column chromatography, eluting with hexane/EtOAc, to afford a pure product of compound P16. (X: Br; isolated yield after column chromatography: 89%, 272 mg).

IR (KBr), ν (cm^{-1}): 3031, 1620, 1479. ^{1}H NMR (400 MHz, $CDCl_3$): δ (ppm) 7.36-7.40 (2H, m), 7.48 (4H, t, J = 7.2 Hz), 7.66 (4H, d, J = 9.2 Hz), 7.70 (8H, s). Anal. Calcd for $C_{24}H_{18}$: C, 94.08; H, 5.92; Found: C, 93.17; H, 4.76.

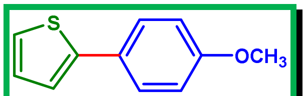

P17

2-(4-Methoxyphenyl)thiophene (Scheme 3, compound P17)
M.p.: 103-105 °C; CAS number: (42545-43-7)
R_f (n-hexane/EtOAc 18:2): 0.49

To a solution of 2-iodothiophene (1.0 mmol, 0.210 g), 4-methoxyphenyl boronic acid (1.2 mmol, 0.182 g), Pd_{np}-$TPEPTA_{(L)}$-GO (30 mg), K_2CO_3 (2.0 mmol, 0.276 g), and DMF: H_2O (2:1) (6.0 mL) were placed in a 25 mL round-bottom-flask equipped with a condenser and magnetic stirring bar and heated at 80 °C. The progress of the reaction was monitored using TLC. After completion of the reaction, 5.0 mL of hot water and 5.0 mL of ethyl acetate or ethanol were added to the reaction mixture. The Pd_{np}-$TPEPTA_{(L)}$-GO catalyst was separated under reduced pressure using vacuum pomp over sinter-glass grade-4. The organic solution was evaporated on rotary evaporator and the crude compound P17 was obtained. The crude product was purified by column chromatography, eluting with hexane/EtOAc, and then recrystalized from ethanol to afford a pure product of compound P17. (X: I; isolated yield after column chromatography: 96%, 182 mg).

^{1}H NMR (250 MHz, $CDCl_3$): δ (ppm) = 3.83 (s, 3H), 6.90-7.06 (m, 3H), 7.20-7.26 (m, 2H), 7.53 (d, J = 4.7 Hz, 2H). ^{13}C NMR (62.5 MHz, $CDCl_3$): δ (ppm) = 55.4, 114.1, 122.0, 123.8, 127.2, 127.4, 127.8, 143.9, 159.1.

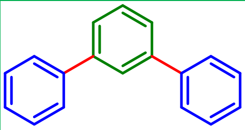

P18

1,3-Diphenylbenzene (Scheme 3, compound P18)
M.p.: 135-138 °C; CAS number: (92-06-8)
R_f(*n*-hexane/EtOAc 18:2): 0.33

To a solution of 1,3-dibromobenzene or 1,3-dichlorobenzene (1.0 mmol), phenyl boronic acid (2.4 mmol, 0.292 g), Pd_{np}-$TPEPTA_{(L)}$-GO (30 mg), K_2CO_3 (4.0 mmol, 0.552 g), and DMF: H_2O (2:1) (6.0 mL) were placed in a 25 mL round-bottom-flask equipped with a condenser and magnetic stirring bar and heated at 80 °C. The progress of the reaction was monitored using TLC. After completion of the reaction, 5.0 mL of hot water and 5.0 mL of ethyl acetate or ethanol were added to the reaction mixture. The Pd_{np}-$TPEPTA_{(L)}$-GO catalyst was separated under reduced pressure using vacuum pomp over sinter-glass grade-4. The organic solution was evaporated on rotary evaporator and the crude compound P18 was obtained. The crude product was purified by column chromatography, eluting with hexane/EtOAc, to afford a pure product of compound P18. (X: Br; isolated yield after column chromatography: 90%, 207 mg), (X: Cl; isolated yield after column chromatography: 88%, 202 mg).

IR (KBr): ν (cm^{-1}) 3076, 1588, 1426, 735. ^1H NMR (400 MHz, $CDCl_3$) δ(ppm): 7.37-7.51 (7H, m, phenyl), 7.57 (2H, d, *J=7.6 Hz,* phenyl), 7.64 (4H, d, *J=7.8 Hz,* phenyl), 7.80 (1H, s, phenyl).

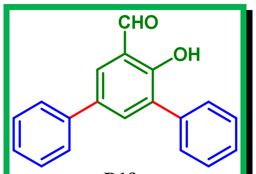

P19

2-Hydroxy-3,5-diphenylbenzaldehyde (Scheme 3, compound P19)
M.p.:114-116 °C;
R_f(*n*-hexane/EtOAc 18:2): 0.62

To a solution of 2-hydroxy-3,5-diiodobenzaldehyde (1.0 mmol, 0.373 g), phenyl boronic acid (2.4 mmol, 0.292 g), Pd_{np}-$TPEPTA_{(L)}$-GO (30 mg), K_2CO_3 (4.0 mmol, 0.552 g), and DMF: H_2O (2:1) (6.0 mL) were placed in a 25 mL round-bottom-flask equipped with a condenser and magnetic stirring bar and heated at 80 °C. The progress of the reaction was monitored using TLC. After completion of the reaction, 5.0 mL of hot water and 5.0 mL of ethyl acetate or ethanol were added to the reaction mixture. The Pd_{np}-$TPEPTA_{(L)}$-GO catalyst was separated under reduced pressure using vacuum pomp over sinter-glass grade-4. The organic solution was evaporated on rotary evaporator and the crude compound P19 was obtained. The crude product was purified by column chromatography, eluting with hexane/EtOAc, to afford a pure product of compound P19. (X: I; isolated yield after column chromatography: 85%, 233 mg).

IR (KBr), ν (cm^{-1}): 3426, 3047, 2848, 1651, 1607, 1459. ^1H NMR (400 MHz, $CDCl_3$): δ (ppm) 7.38-7.44 (2H, m), 7.46-7.50 (4H, m), 7.59-7.67 (4H, m), 7.78 (1H, d, *J* = 2.4 Hz), 7.88 (1H, d, *J* = 2.4 Hz), 10.05 (1H, s), 11.52 (1H, s). Anal. Calcd for $C_{19}H_{14}O_2$: C, 83.19; H, 5.14; Found: C, 83.01; H, 4.96.

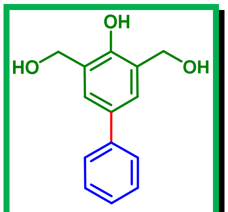

P20

(4-hydroxy-[1,1'-biphenyl]-3,5-diyl)dimethanol (Scheme 3, compound P20)
M.p.: 90-93 °C;
R_f(*n*-hexane/EtOAc 18:2): 0.69

To a solution of 2,6-bis(hydroxymethyl)-4-bromophenol or 2,6-bis(hydroxymethyl)-4-chlorophenol (1.0 mmol), phenyl boronic acid (1.2 mmol, 0.146 g), Pd_{np}-$TPEPTA_{(L)}$-GO (30 mg), K_2CO_3 (2.0 mmol, 0.276 g), and DMF: H_2O (2:1) (6.0 mL) were placed in a 25 mL round-bottom-flask equipped with a condenser and magnetic stirring bar and

heated at 80 °C. The progress of the reaction was monitored using TLC. After completion of the reaction, 5.0 mL of hot water and 5.0 mL of ethyl acetate or ethanol were added to the reaction mixture. The Pd_{np}-$TPEPTA_{(L)}$-GO catalyst was separated under reduced pressure using vacuum pomp over sinter-glass grade-4. The organic solution was evaporated on rotary evaporator and the crude compound P20 was obtained. The crude product was purified by column chromatography, eluting with hexane/EtOAc, and then recrystalized from ethanol to afford a pure product of compound P20. (X: Br; isolated yield after column chromatography: 90%, 207 mg), (X: Cl; isolated yield after column chromatography: 87%, 200 mg).

IR (KBr), ν (cm^{-1}): 3412, 3028, 1601, 1480. ^{1}H NMR (400 MHz, $CDCl_3$): δ (ppm) 4.90 (2H, s), 5.23 (2H, s), 7.13 (1H, s), 7.44 (2H, t, J = 7.2 Hz), 7.49 (1H, s), 7.54 (2H, t, J = 7.6 Hz), 7.93 (1H, s), 7.95 (1H, s). Anal. Calcd for $C_{14}H_{14}O_3$: C, 73.03; H, 6.13; Found: C, 72.17; H, 5.36.

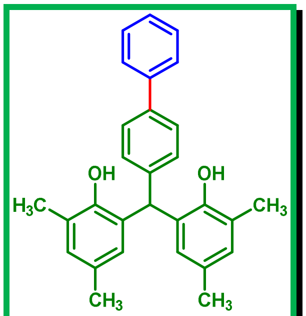

P21

6,6'-([1,1'-biphenyl]-4-ylmethylene)bis(2,4-dimethylphenol) (Scheme 3, compound P21)
M.p: 108-110 °C
R_f(*n*-hexane/EtOAc 18:2): 0.51
For general procedure, please see part 1.8 in supporting information.

IR (KBr): ν (cm^{-1}) 3339, 3021, 2920, 1600, 1481, 1447, 1384, 1292, 1186, 1142, 864, 702. ^{1}H-NMR (400 MHz, DMSO-d_6) δ(ppm): 2.06 (6H, s, Me), 2.08 (6H, s, Me), 6.13 (1H, s, Ar_3CH), 6.29 (2H, s, OH), 6.70 (2H, s, 3-H DMP), 6.97 (3H, t, *J=8 Hz*), 7.11 (2H, t, *J=8 Hz*), 7.19 (2H, d, *J=8 Hz*), 7.23 (2H, d, *J=8 Hz*), 7.93 (2H, s). Anal. Calcd for $C_{29}H_{28}O_2$: C, 85.26; H, 6.91. Found: C, 84.94; H, 6.74.

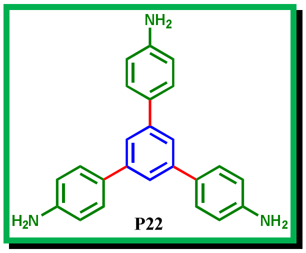

P22

1,3,5-Tris(4-aminophenyl)benzene (compound P22)
M.p.: 162-164 °C; CAS number (118727-34-7)
R_f(*n*-hexane/EtOAc 18:2): 0.37
For general procedure, please see part 1.9 in the supporting information.

IR (KBr): ν (cm^{-1}) 3420, 3006, 2931, 1589, 1531, 1432, 1382, 1086, 864, 702. ^{1}H-NMR (250 MHz, DMSO-d_6) δ(ppm): 7.51 (s, 3H), 7.49 (d, *J= 8.8 Hz*, 6H), 6.68 (d, *J= 8.8 Hz*, 6H), 5.21 (s, 6H); ^{13}C NMR (62.5 NHz, DMSO-d_6) δ(ppm): 114.6, 120.8, 127.9, 128.5, 142.0, 148.8.

Hard copies of ^{1}H NMR and ^{13}C NMR of the synthesized compounds

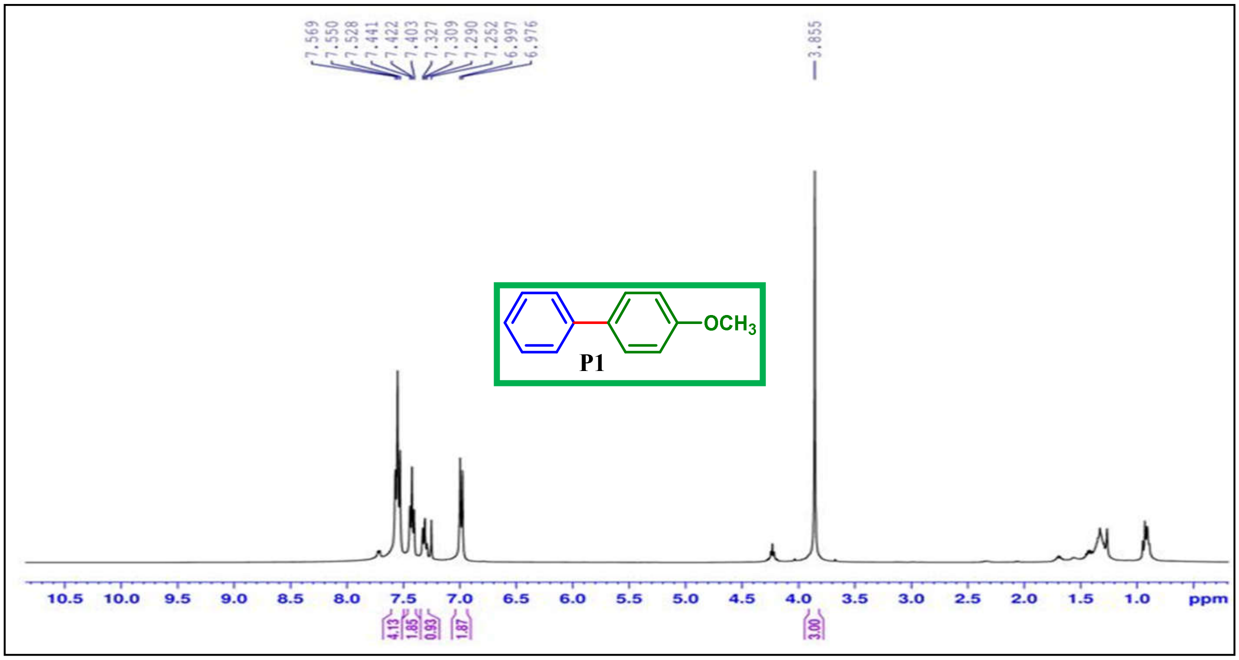

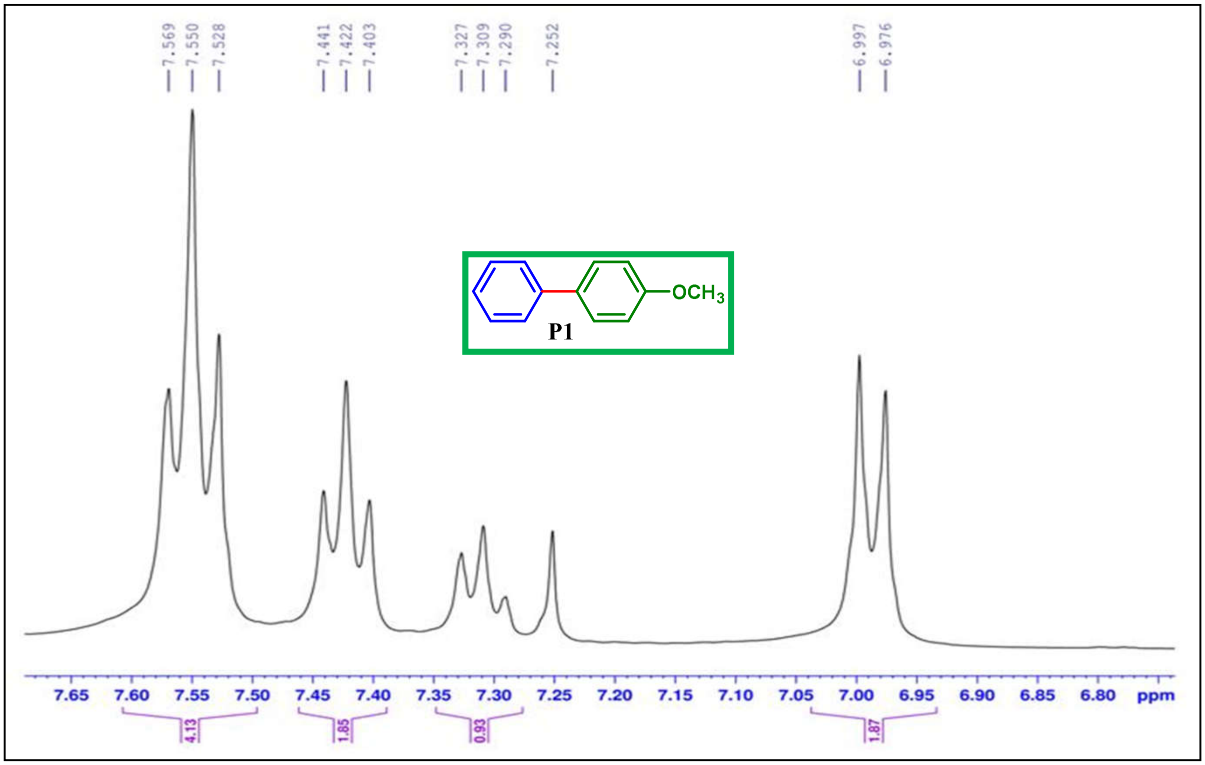

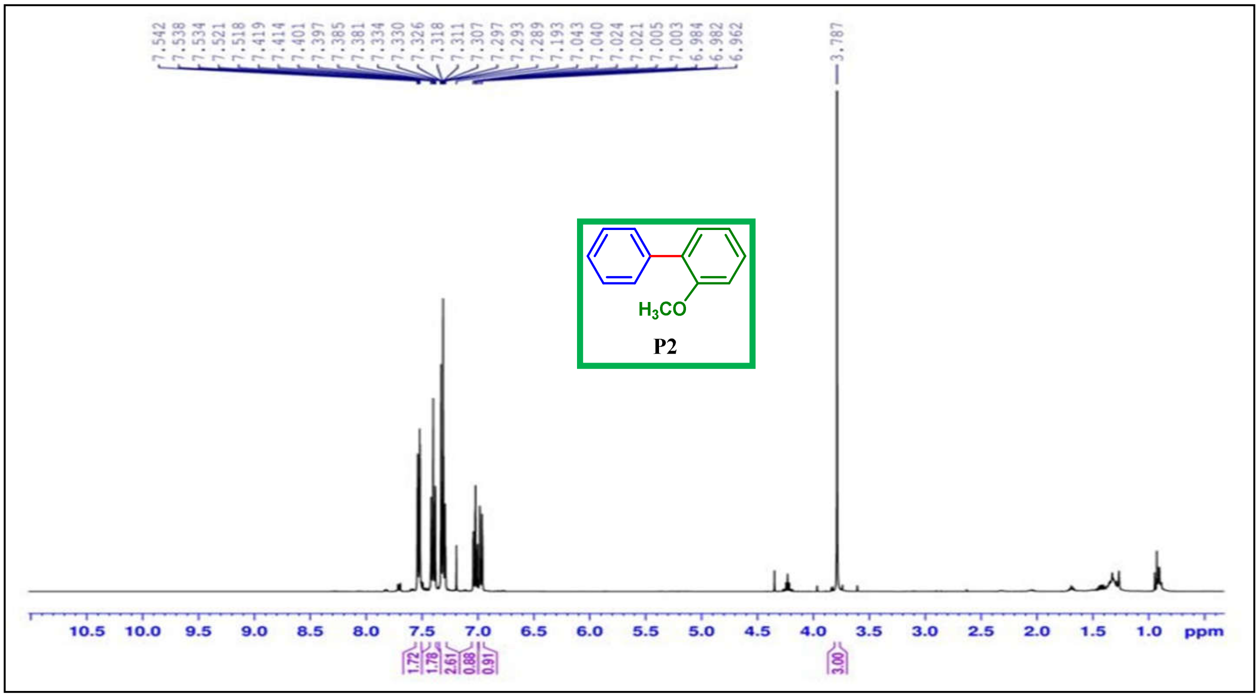
7.542
7.538
7.534
7.521
7.518
7.419
7.414
7.401
7.397
7.385
7.381
7.334
7.330
7.326
7.318
7.311
7.307
7.297
7.293
7.289
7.193
7.043
7.040
7.024
7.021
7.005
7.003
6.984
6.982
6.962
3.787
H3CO
P2
10.5 10.0 9.5 9.0 8.5 8.0 7.5 7.0 6.5 6.0 5.5 5.0 4.5 4.0 3.5 3.0 2.5 2.0 1.5 1.0 ppm
1.72
1.78
2.61
0.88
0.91
3.00

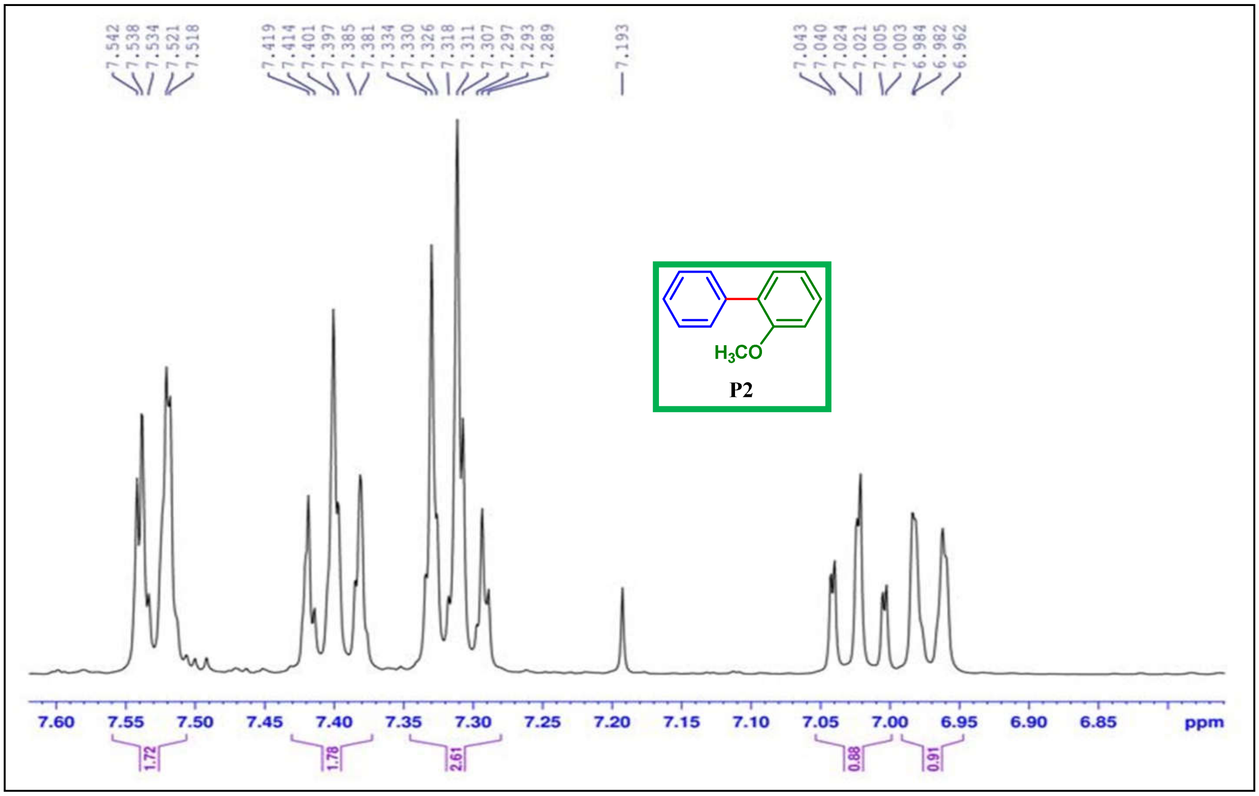
7.542
7.538
7.534
7.521
7.518
7.419
7.414
7.401
7.397
7.385
7.381
7.334
7.330
7.326
7.318
7.311
7.307
7.297
7.293
7.289
7.193
7.043
7.040
7.024
7.021
7.005
7.003
6.984
6.982
6.962
H3CO
P2
7.60 7.55 7.50 7.45 7.40 7.35 7.30 7.25 7.20 7.15 7.10 7.05 7.00 6.95 6.90 6.85 ppm
1.72
1.78
2.61
0.88
0.91

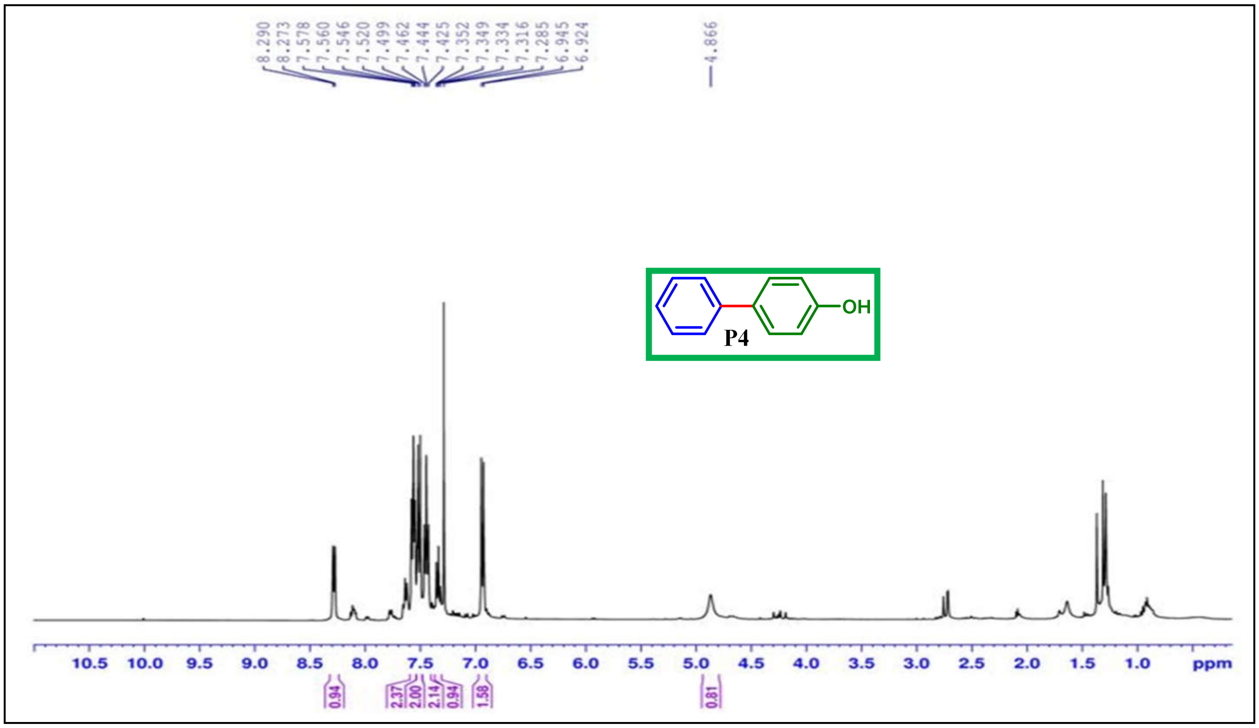
8.290
8.273
7.578
7.560
7.546
7.520
7.499
7.462
7.444
7.425
7.352
7.349
7.334
7.316
7.285
6.945
6.924
4.866
OH
P4
ppm
0.94
2.37
2.00
2.14
0.94
1.58
0.81

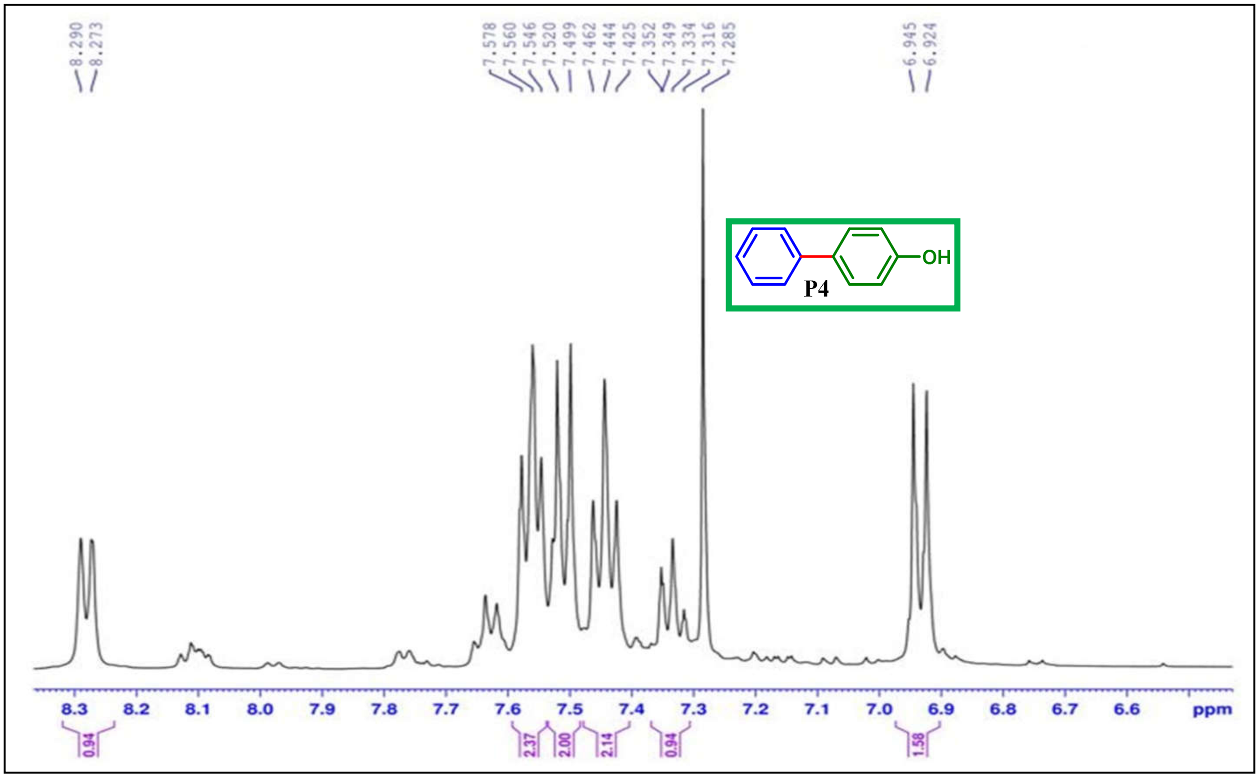
8.290
8.273
7.578
7.560
7.546
7.520
7.499
7.462
7.444
7.425
7.352
7.349
7.334
7.316
7.285
6.945
6.924
OH
P4
ppm
0.94
2.37
2.00
2.14
0.94
1.58

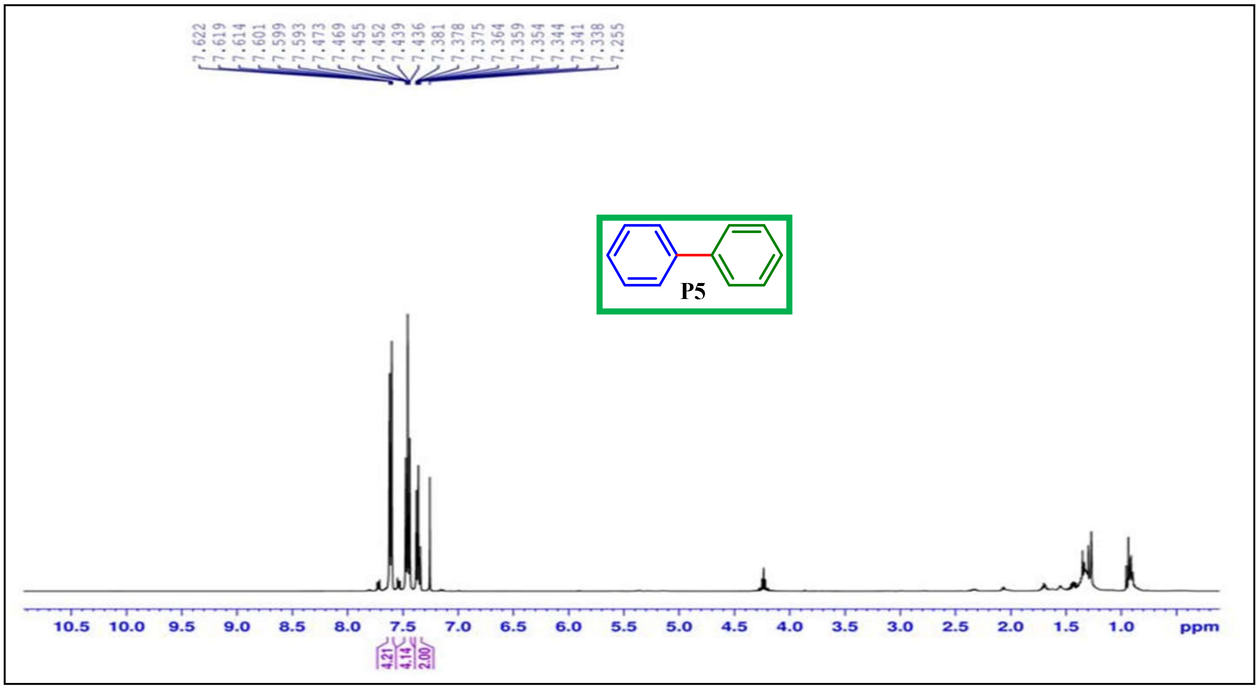
7.622
7.619
7.614
7.601
7.599
7.593
7.473
7.469
7.455
7.452
7.439
7.436
7.381
7.378
7.375
7.364
7.359
7.354
7.344
7.341
7.338
7.255
P5
10.5 10.0 9.5 9.0 8.5 8.0 7.5 7.0 6.5 6.0 5.5 5.0 4.5 4.0 3.5 3.0 2.5 2.0 1.5 1.0 ppm
4.21
4.14
2.00

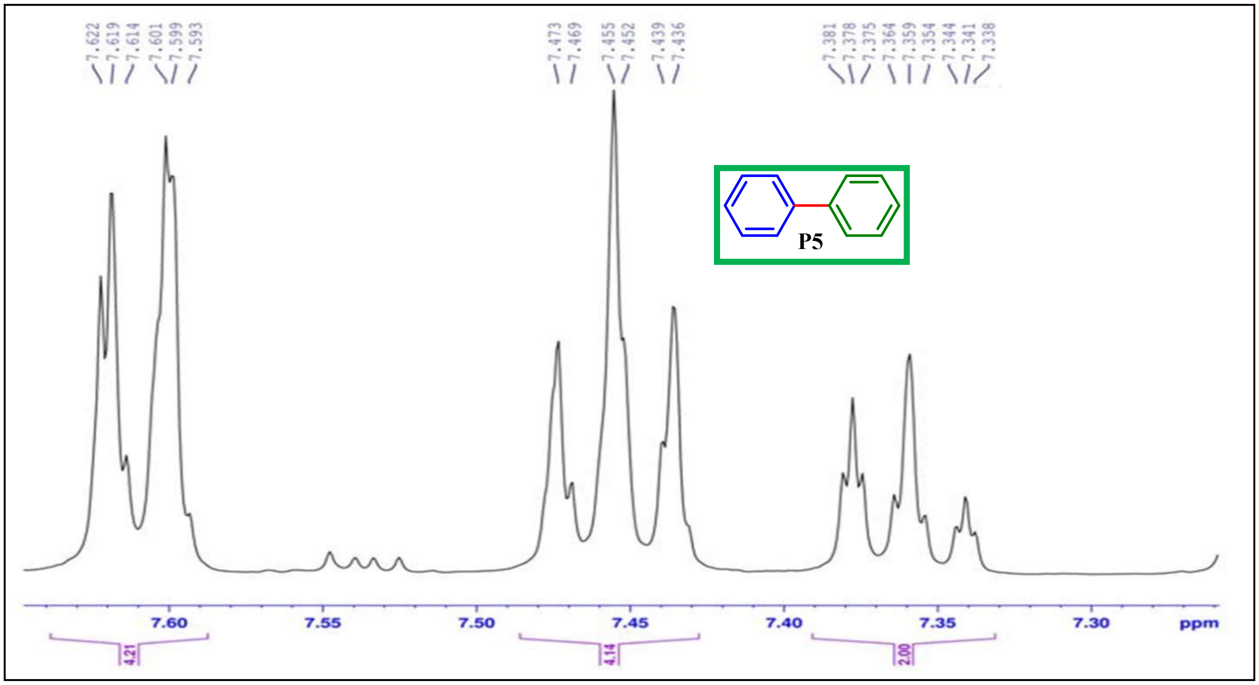
7.622
7.619
7.614
7.601
7.599
7.593
7.473
7.469
7.455
7.452
7.439
7.436
7.381
7.378
7.375
7.364
7.359
7.354
7.344
7.341
7.338
P5
7.60 7.55 7.50 7.45 7.40 7.35 7.30 ppm
4.21
4.14
2.00

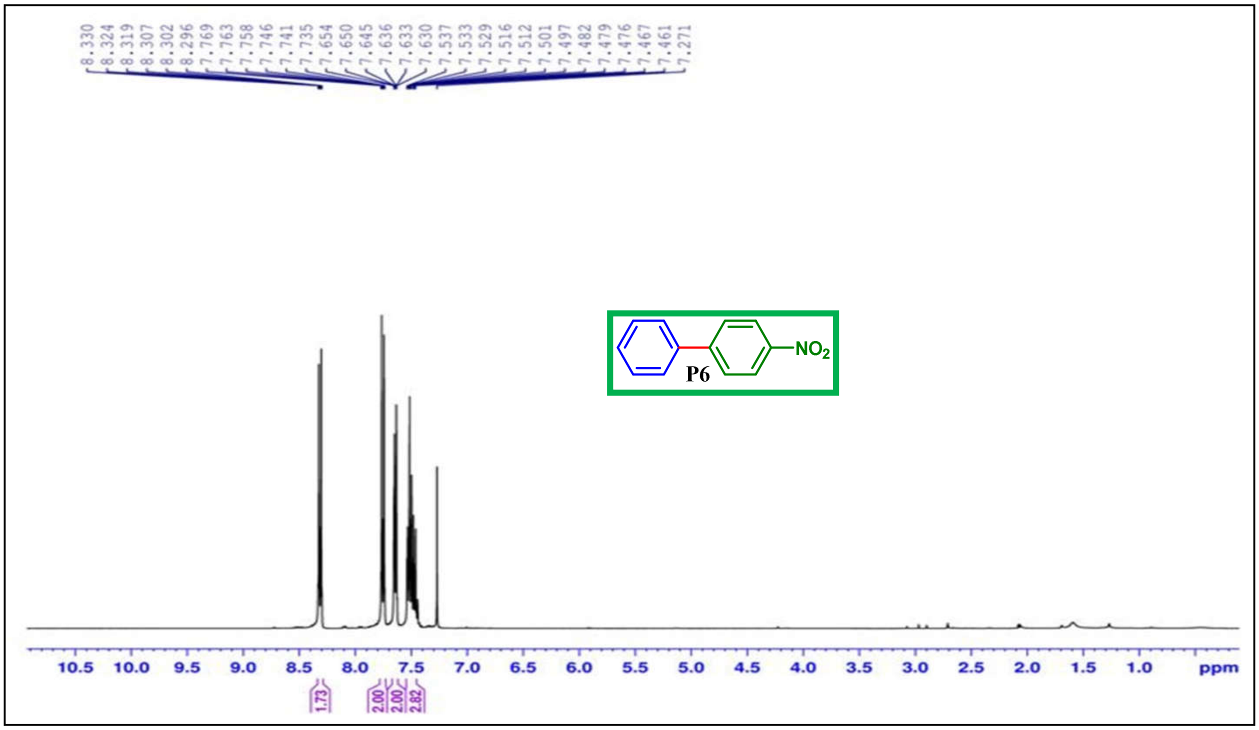
8.330 8.324 8.319 8.307 8.302 8.296 7.769 7.763 7.758 7.746 7.741 7.735 7.654 7.650 7.645 7.636 7.633 7.630 7.537 7.533 7.529 7.516 7.512 7.501 7.497 7.482 7.479 7.476 7.467 7.461 7.271
NO2
P6
10.5 10.0 9.5 9.0 8.5 8.0 7.5 7.0 6.5 6.0 5.5 5.0 4.5 4.0 3.5 3.0 2.5 2.0 1.5 1.0 ppm
1.73 2.00 2.00 2.82

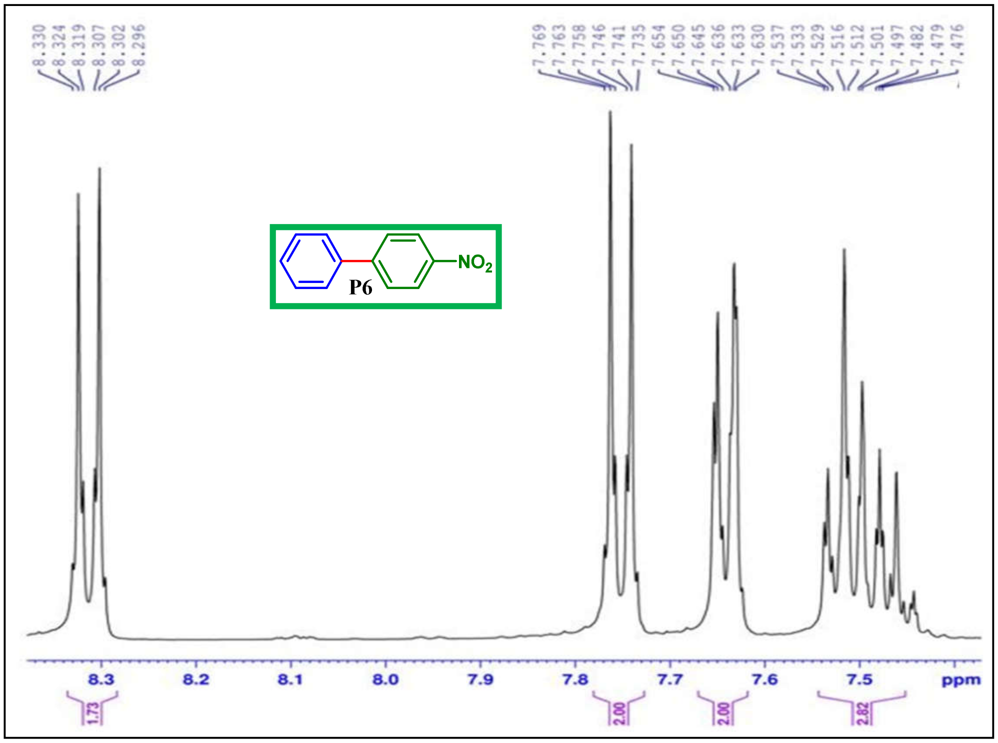
8.330 8.324 8.319 8.307 8.302 8.296
7.769 7.763 7.758 7.746 7.741 7.735 7.654 7.650 7.645 7.636 7.633 7.630 7.537 7.533 7.529 7.516 7.512 7.501 7.497 7.482 7.479 7.476
NO2
P6
8.3 8.2 8.1 8.0 7.9 7.8 7.7 7.6 7.5 ppm
1.73 2.00 2.00 2.82

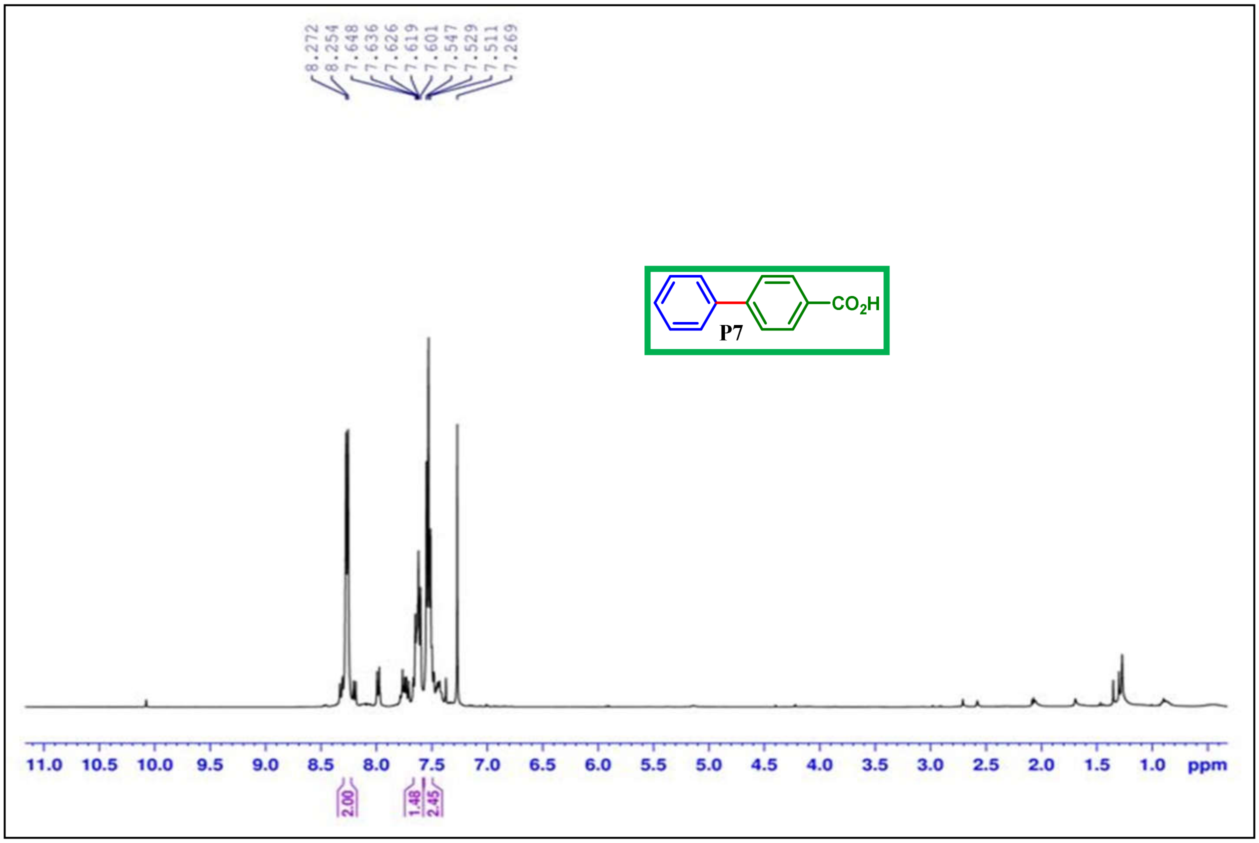
8.272
8.254
7.648
7.636
7.626
7.619
7.601
7.547
7.529
7.511
7.269
CO2H
P7
11.0 10.5 10.0 9.5 9.0 8.5 8.0 7.5 7.0 6.5 6.0 5.5 5.0 4.5 4.0 3.5 3.0 2.5 2.0 1.5 1.0 ppm
2.00
1.48
2.45

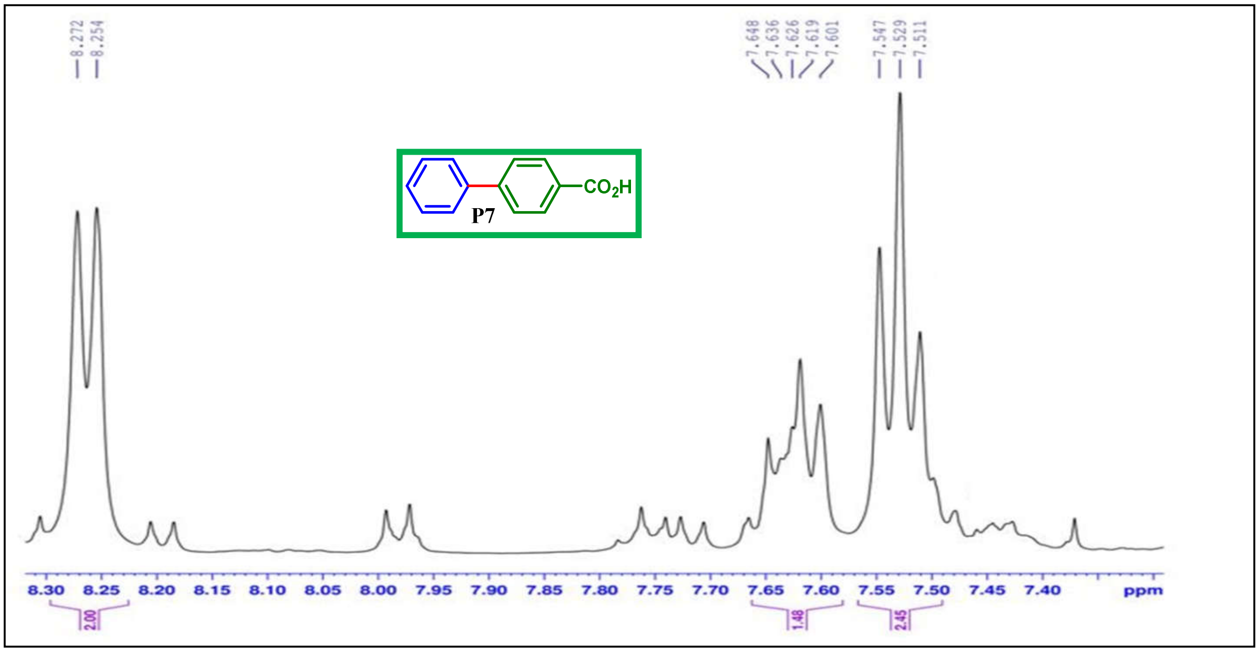
8.272
8.254
7.648
7.636
7.626
7.619
7.601
7.547
7.529
7.511
CO2H
P7
8.30 8.25 8.20 8.15 8.10 8.05 8.00 7.95 7.90 7.85 7.80 7.75 7.70 7.65 7.60 7.55 7.50 7.45 7.40 ppm
2.00
1.48
2.45

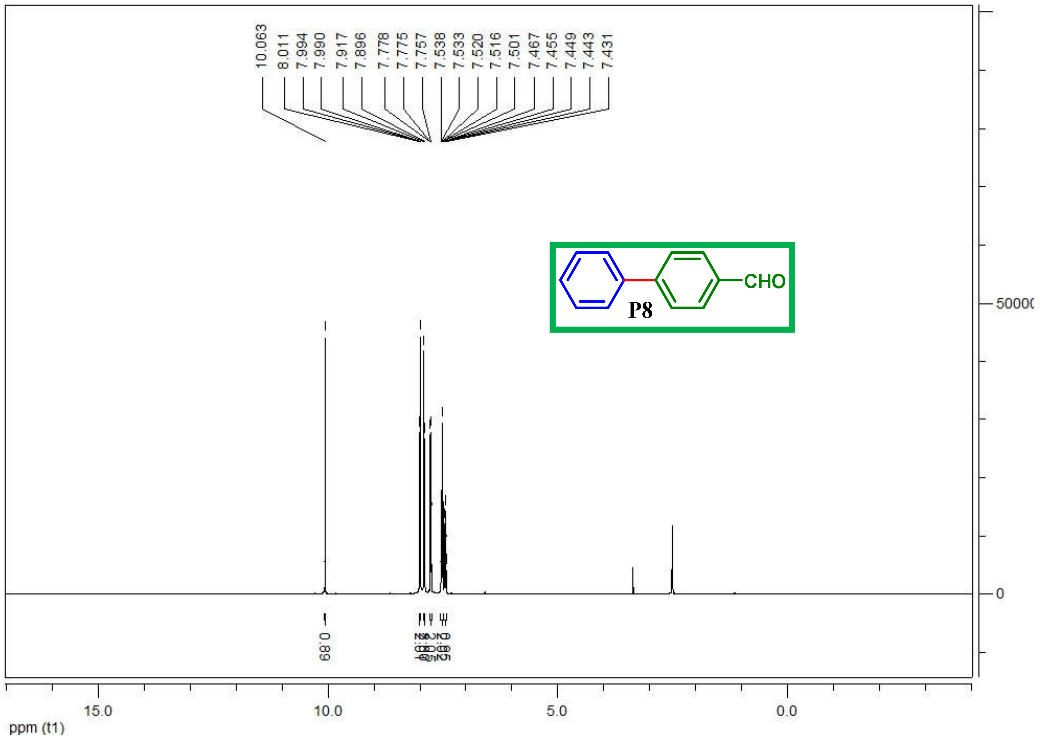
10.063
8.011
7.994
7.990
7.917
7.896
7.778
7.775
7.757
7.538
7.533
7.520
7.516
7.501
7.467
7.455
7.449
7.443
7.431
CHO
P8
0.89
15.0
10.0
5.0
0.0
ppm (t1)
50000
0

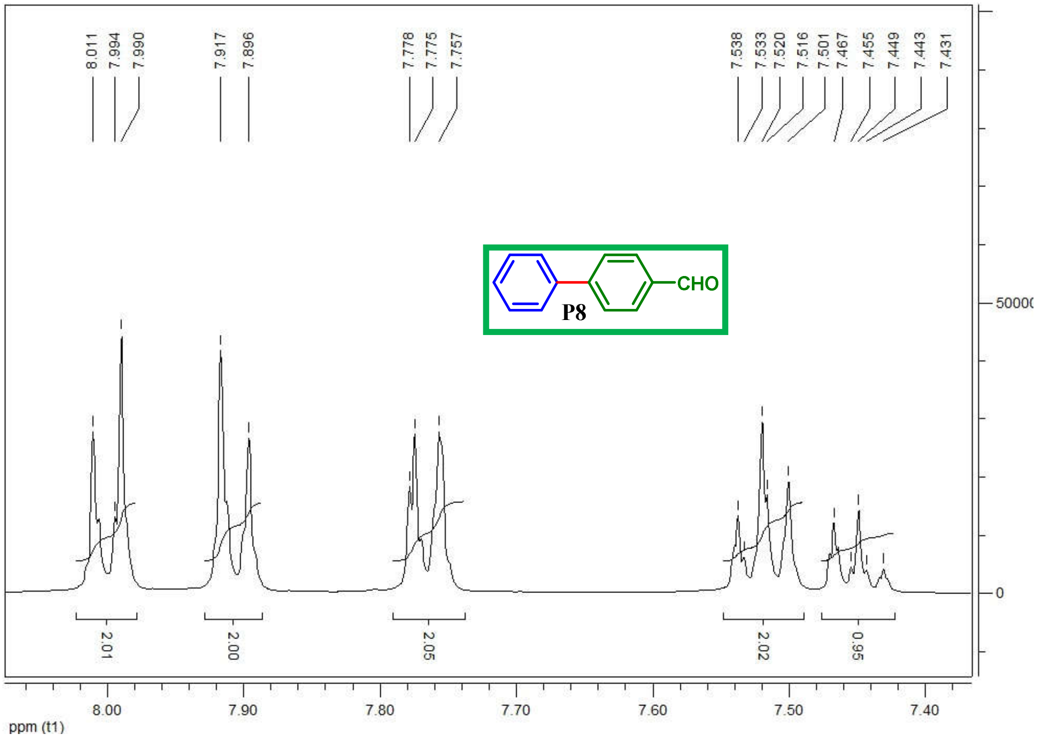
8.011
7.994
7.990
7.917
7.896
7.778
7.775
7.757
7.538
7.533
7.520
7.516
7.501
7.467
7.455
7.449
7.443
7.431
CHO
P8
2.01
2.00
2.05
2.02
0.95
8.00
7.90
7.80
7.70
7.60
7.50
7.40
ppm (t1)
50000
0

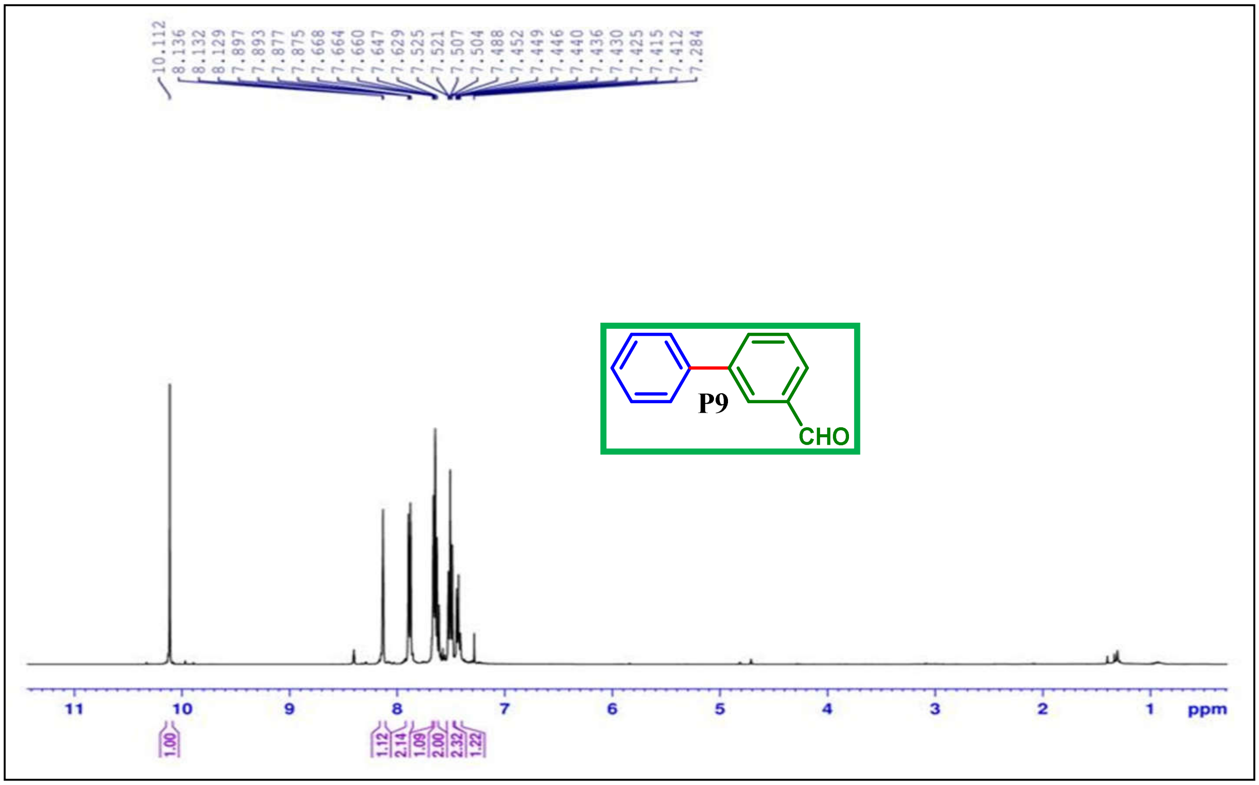
10.112
8.136
8.132
8.129
7.897
7.893
7.877
7.875
7.668
7.664
7.660
7.647
7.629
7.525
7.521
7.507
7.504
7.488
7.452
7.449
7.446
7.440
7.436
7.430
7.425
7.415
7.412
7.284
P9
CHO
11 10 9 8 7 6 5 4 3 2 1 ppm
1.00
1.12
2.14
1.09
2.00
2.32
1.22

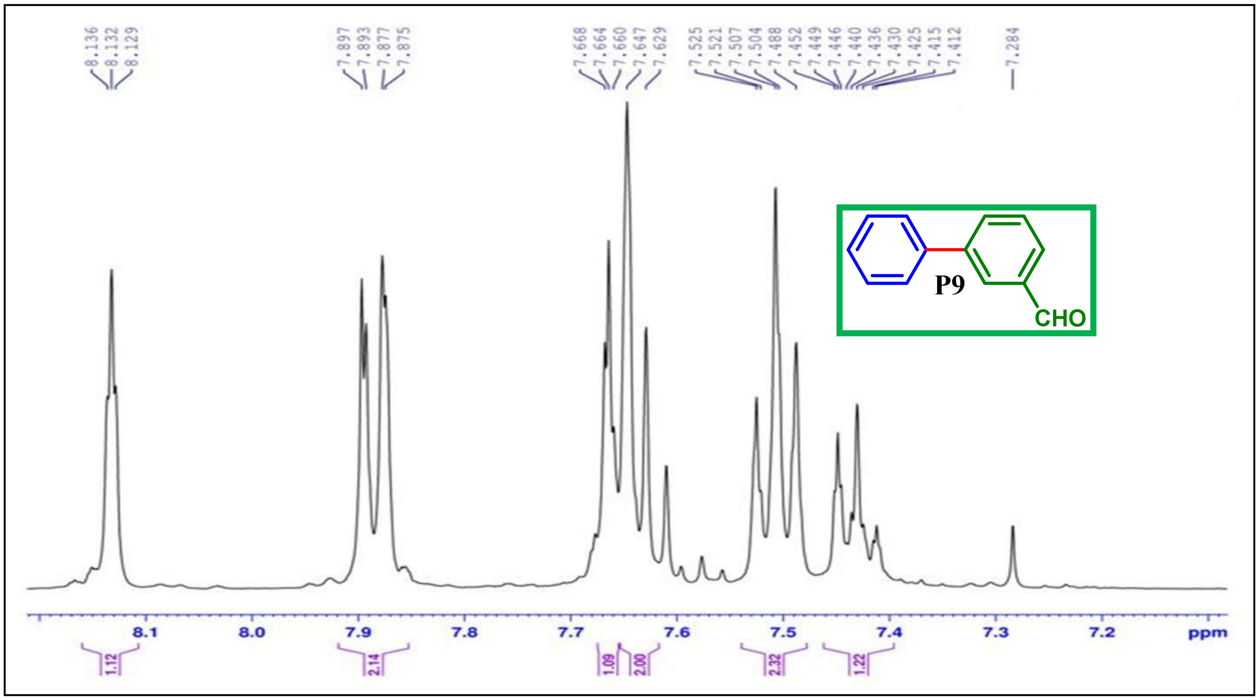
8.136
8.132
8.129
7.897
7.893
7.877
7.875
7.668
7.664
7.660
7.647
7.629
7.525
7.521
7.507
7.504
7.488
7.452
7.449
7.446
7.440
7.436
7.430
7.425
7.415
7.412
7.284
P9
CHO
8.1 8.0 7.9 7.8 7.7 7.6 7.5 7.4 7.3 7.2 ppm
1.12
2.14
1.09
2.00
2.32
1.22

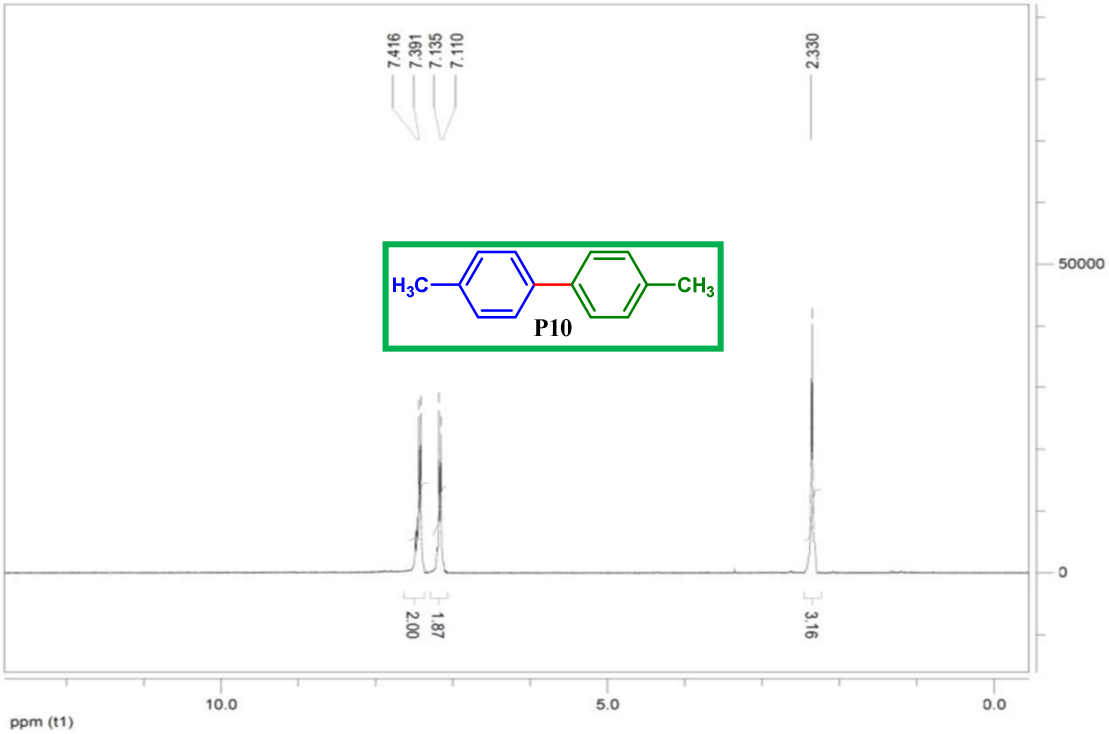
7.416
7.391
7.135
7.110
2.330
H3C
CH3
P10
50000
0
2.00
1.87
3.16
10.0
5.0
0.0
ppm (t1)

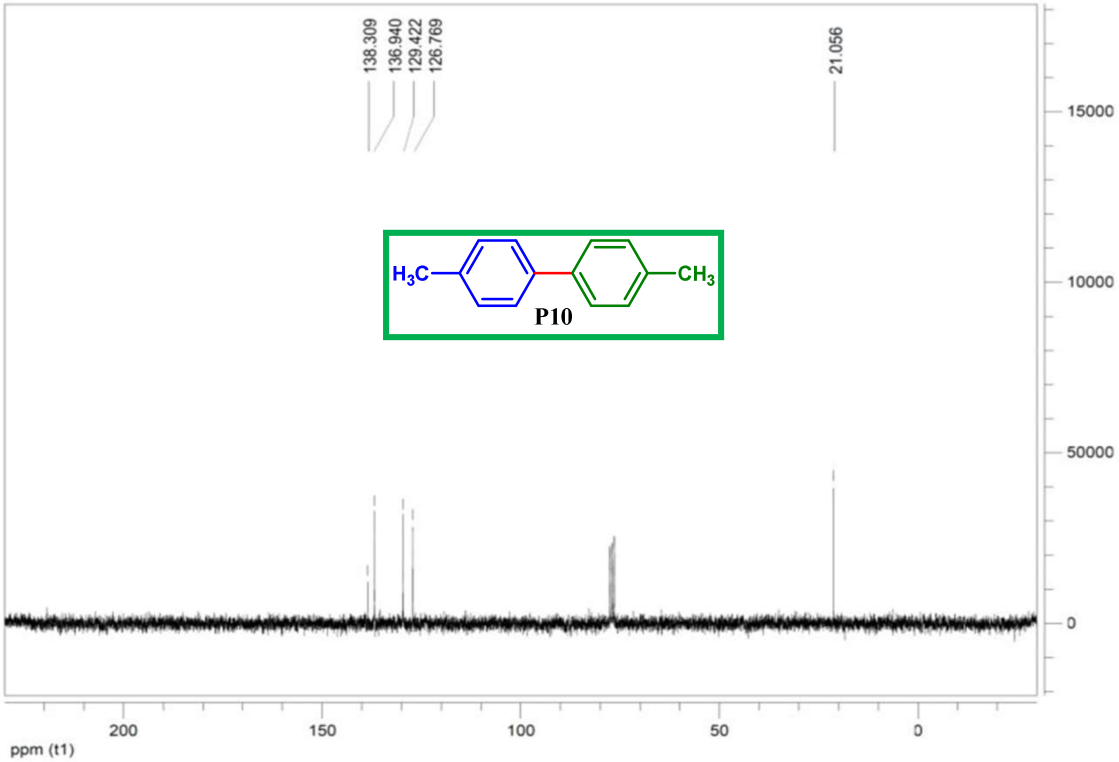
138.309
136.940
129.422
126.769
21.056
H3C
CH3
P10
15000
10000
50000
0
200
150
100
50
0
ppm (t1)

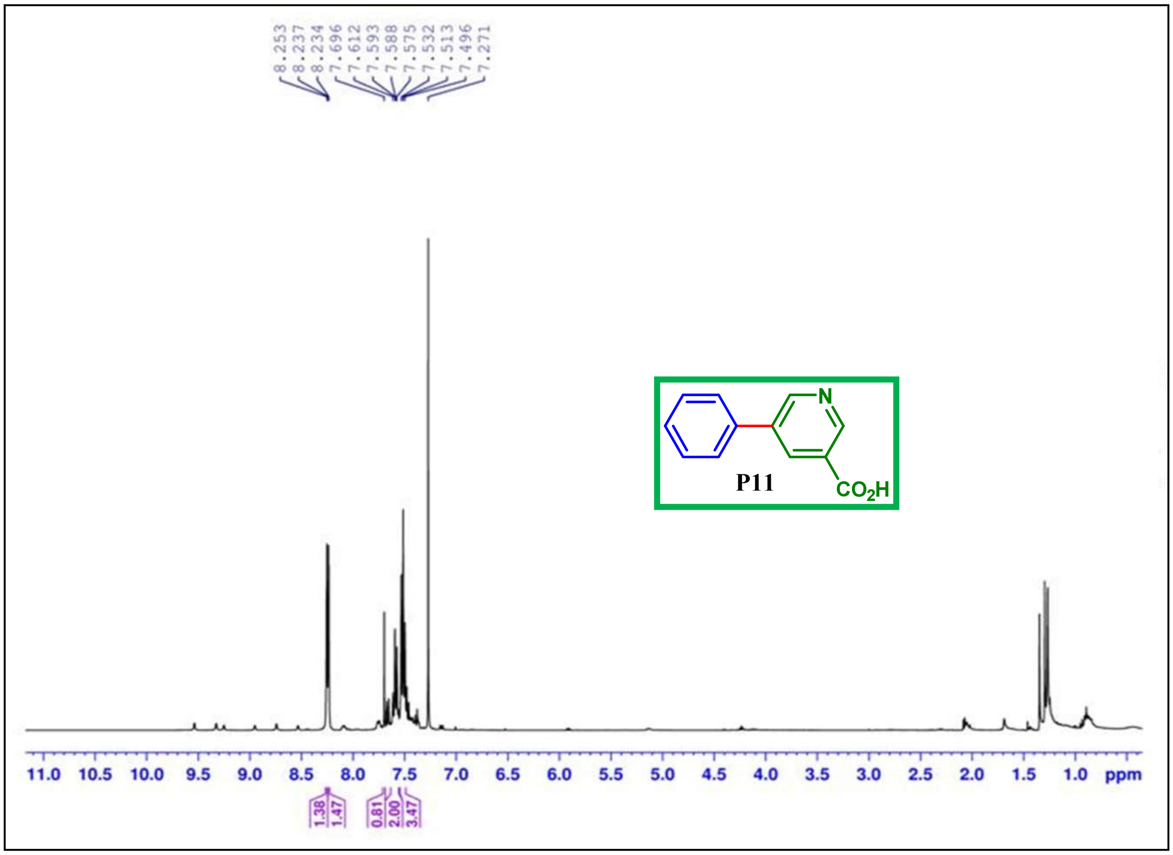
8.253
8.237
8.234
7.696
7.612
7.593
7.588
7.575
7.532
7.513
7.496
7.271
P11
CO_2H
N
11.0 10.5 10.0 9.5 9.0 8.5 8.0 7.5 7.0 6.5 6.0 5.5 5.0 4.5 4.0 3.5 3.0 2.5 2.0 1.5 1.0 ppm
1.38
1.47
0.81
2.00
3.47

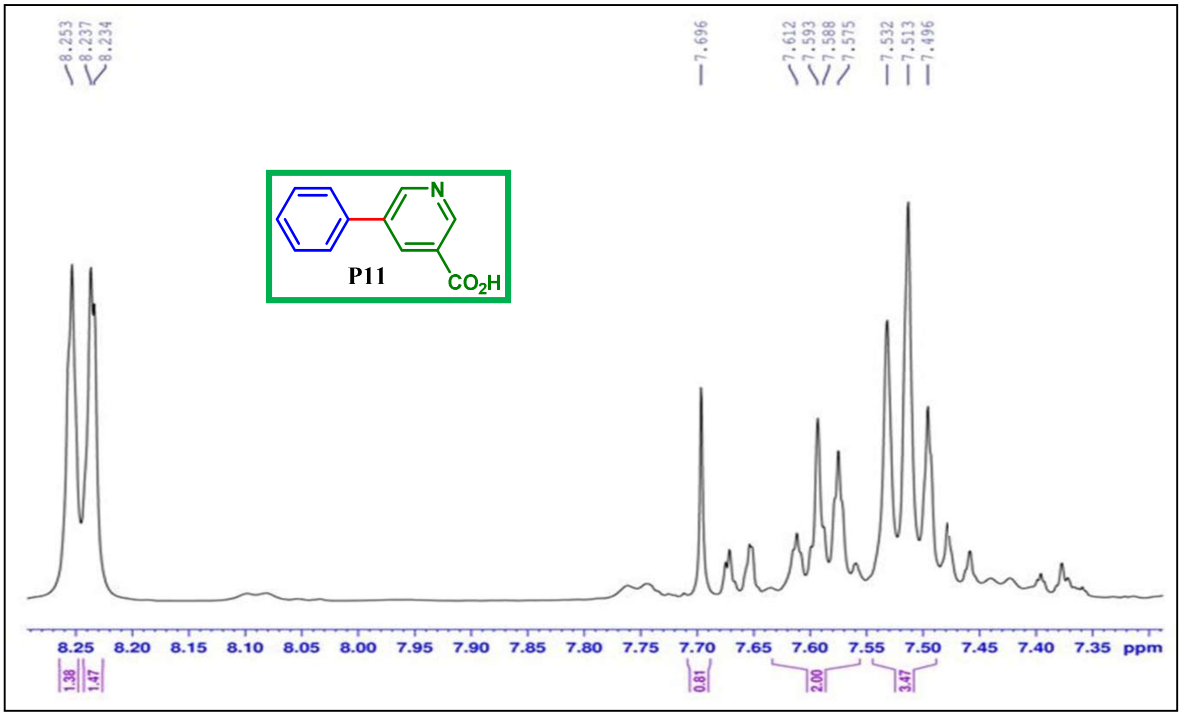
8.253
8.237
8.234
7.696
7.612
7.593
7.588
7.575
7.532
7.513
7.496
P11
CO_2H
N
8.25 8.20 8.15 8.10 8.05 8.00 7.95 7.90 7.85 7.80 7.75 7.70 7.65 7.60 7.55 7.50 7.45 7.40 7.35 ppm
1.38
1.47
0.81
2.00
3.47

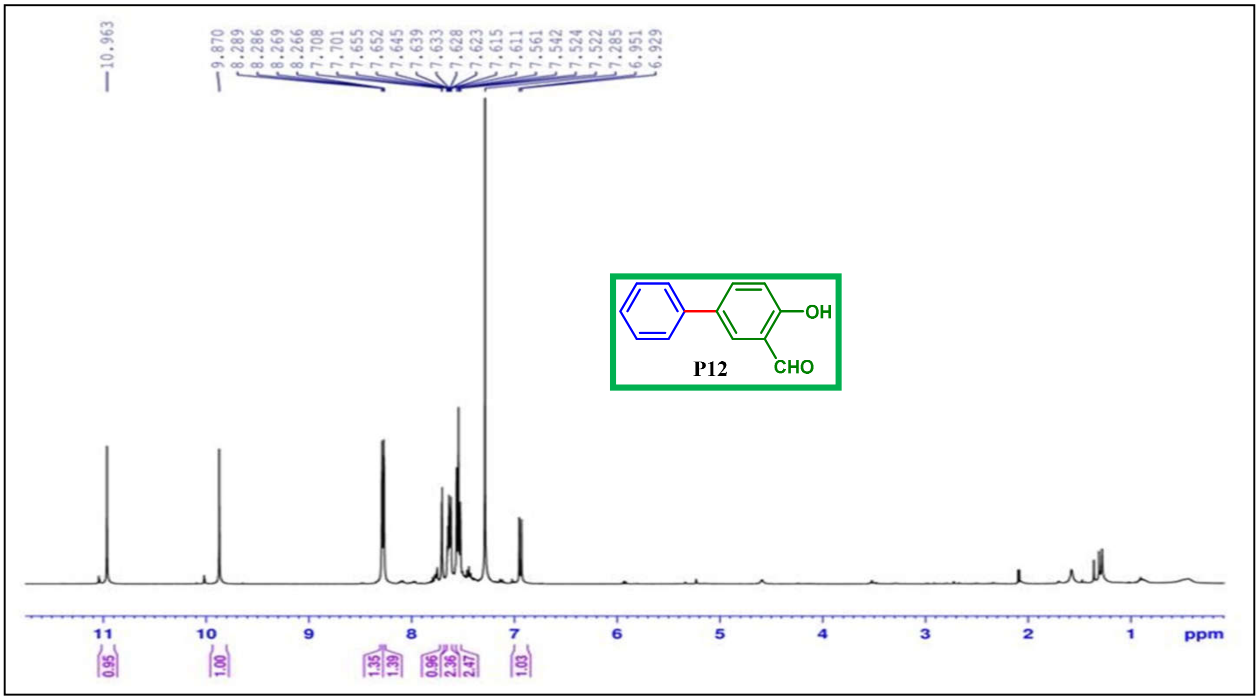

10.963
9.870
8.289
8.286
8.269
8.266
7.708
7.701
7.655
7.652
7.645
7.639
7.633
7.628
7.623
7.615
7.611
7.561
7.542
7.524
7.522
7.285
6.951
6.929
OH
CHO
P12
ppm
0.95
1.00
1.35
1.39
0.96
2.36
2.47
1.03

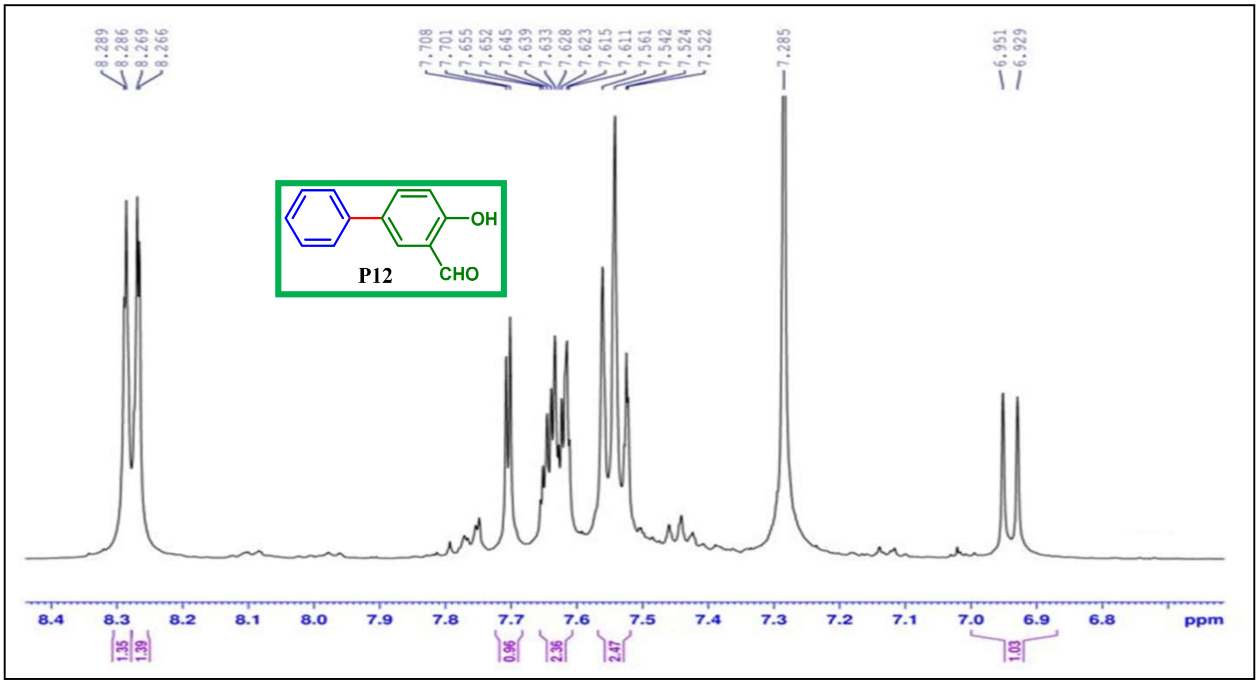

8.289
8.286
8.269
8.266
7.708
7.701
7.655
7.652
7.645
7.639
7.633
7.628
7.623
7.615
7.611
7.561
7.542
7.524
7.522
7.285
6.951
6.929
OH
CHO
P12
ppm
1.35
1.39
0.96
2.36
2.47
1.03

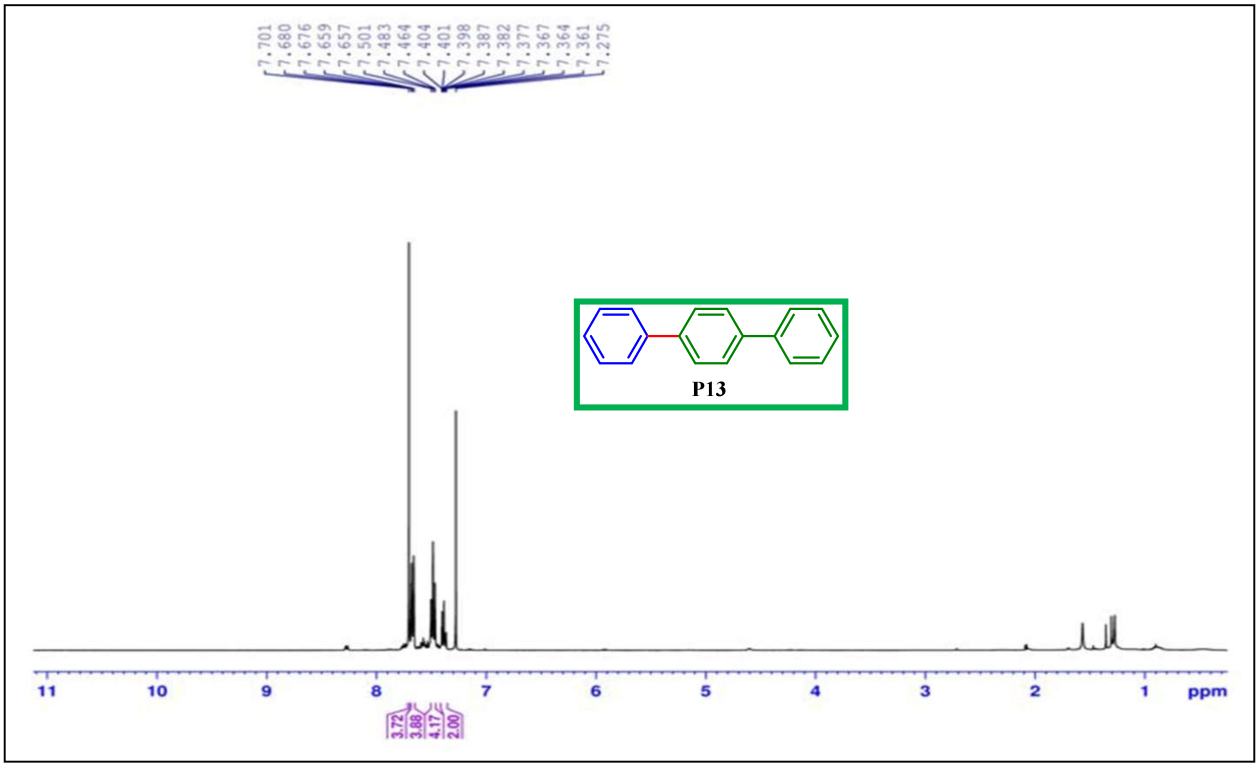
7.701
7.680
7.676
7.659
7.657
7.501
7.483
7.464
7.404
7.401
7.398
7.387
7.382
7.377
7.367
7.364
7.361
7.275
P13
11 10 9 8 7 6 5 4 3 2 1 ppm
3.72
3.88
4.17
2.00

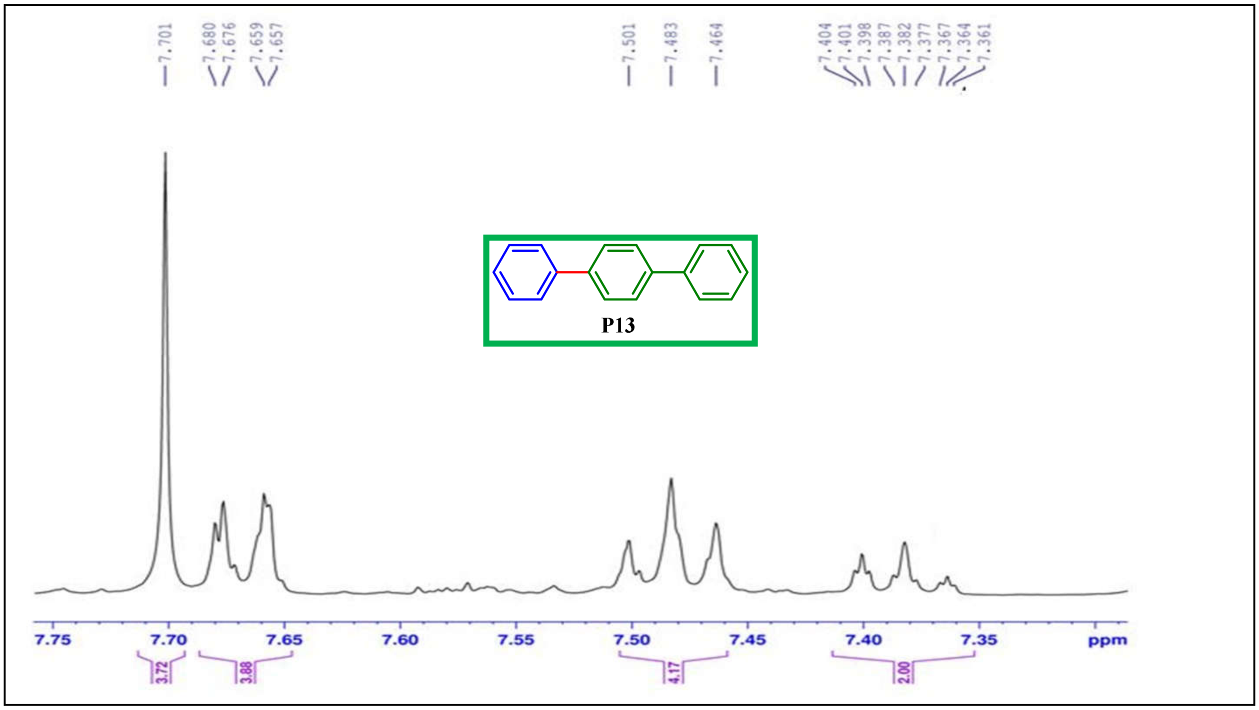
7.701
7.680
7.676
7.659
7.657
7.501
7.483
7.464
7.404
7.401
7.398
7.387
7.382
7.377
7.367
7.364
7.361
P13
7.75 7.70 7.65 7.60 7.55 7.50 7.45 7.40 7.35 ppm
3.72
3.88
4.17
2.00

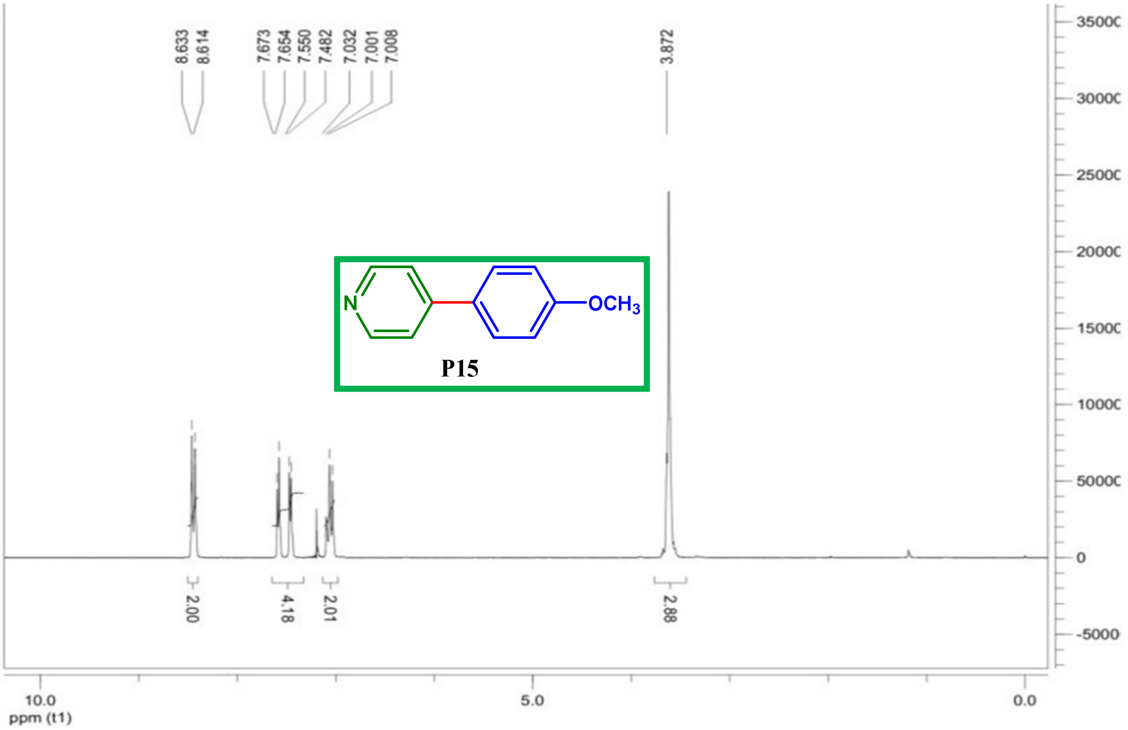
8.633
8.614
7.673
7.654
7.550
7.482
7.032
7.001
7.008
3.872
N
OCH3
P15
2.00
4.18
2.01
2.88
10.0
ppm (t1)
5.0
0.0

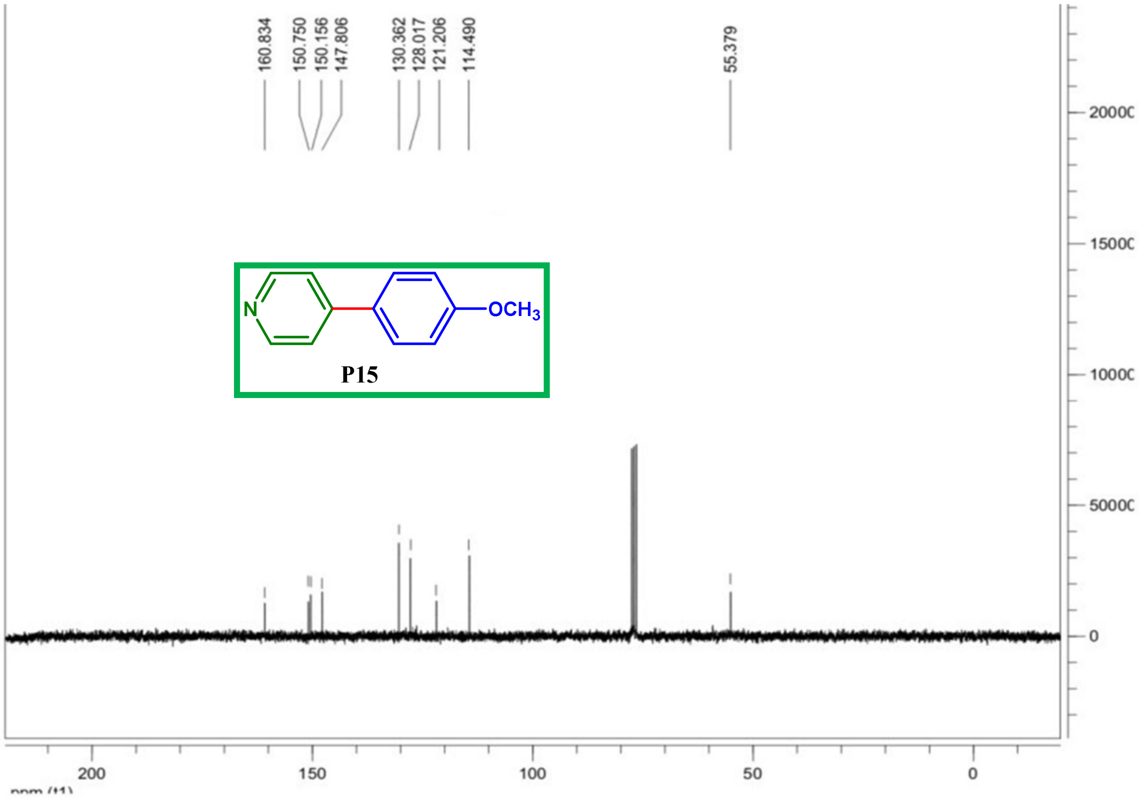
160.834
150.750
150.156
147.806
130.362
128.017
121.206
114.490
55.379
N
OCH3
P15
200
150
100
50
0

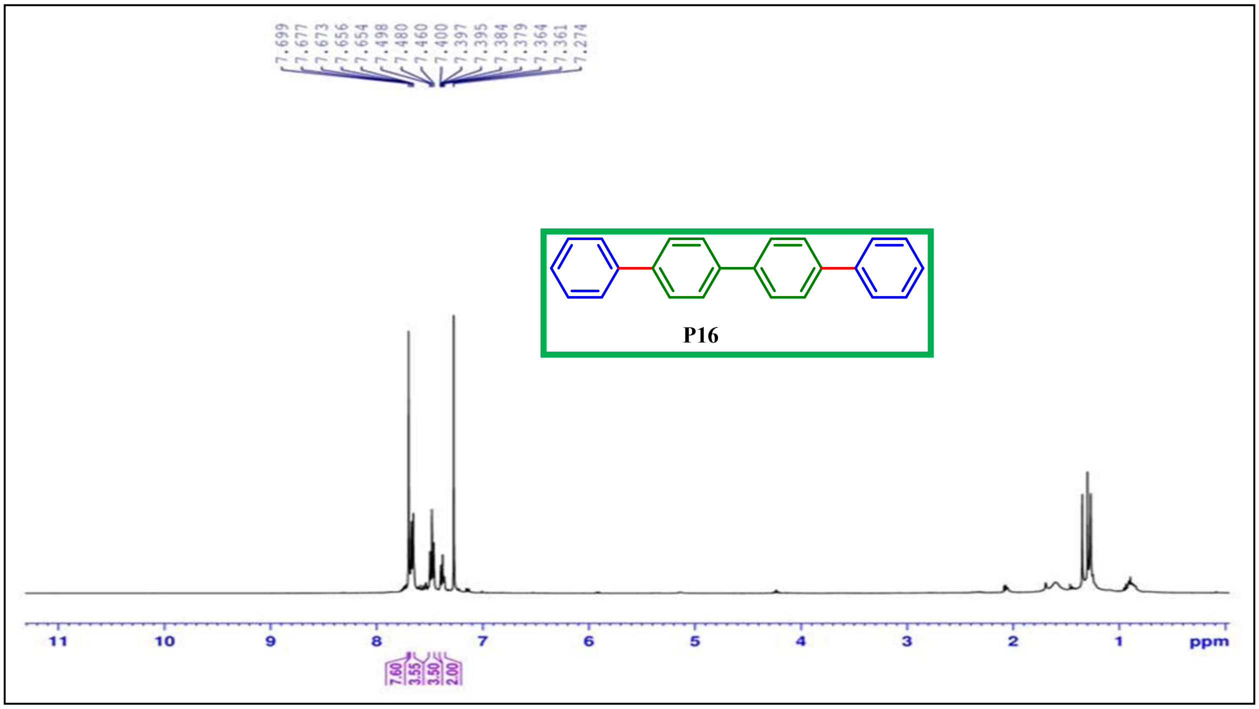
P16
ppm

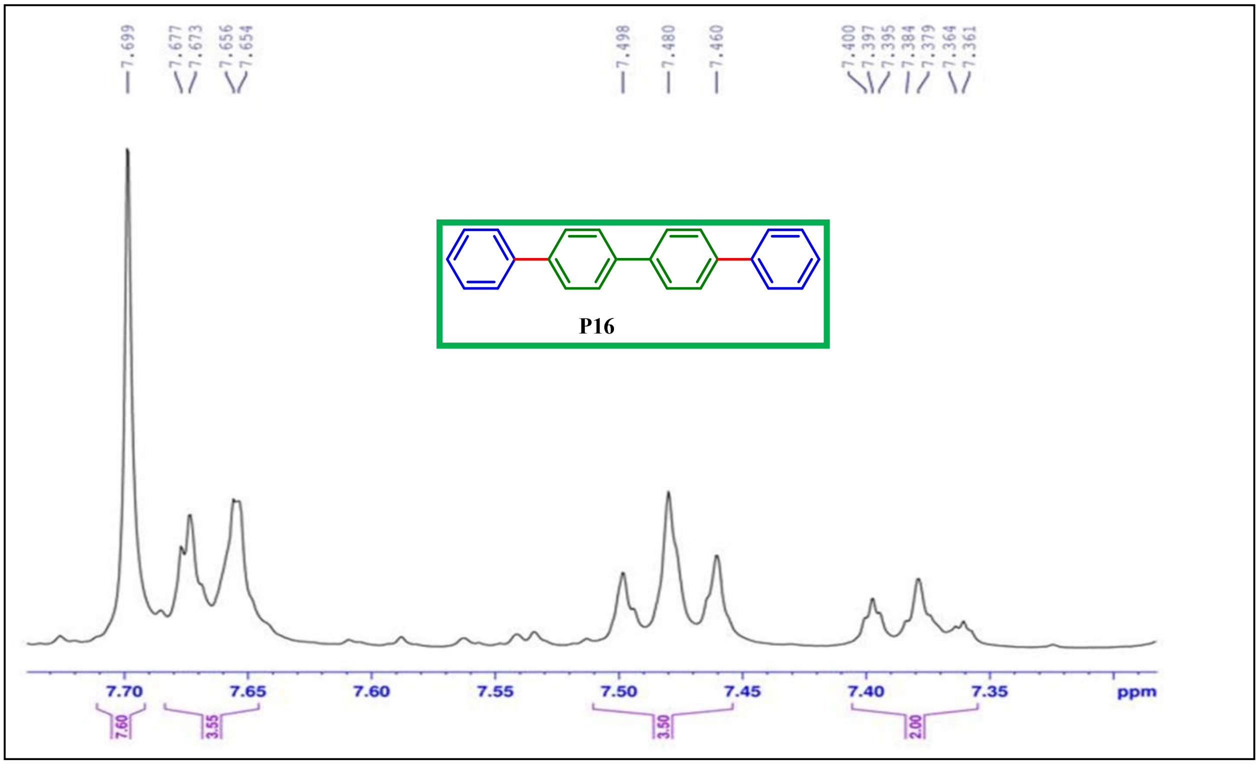
P16
ppm

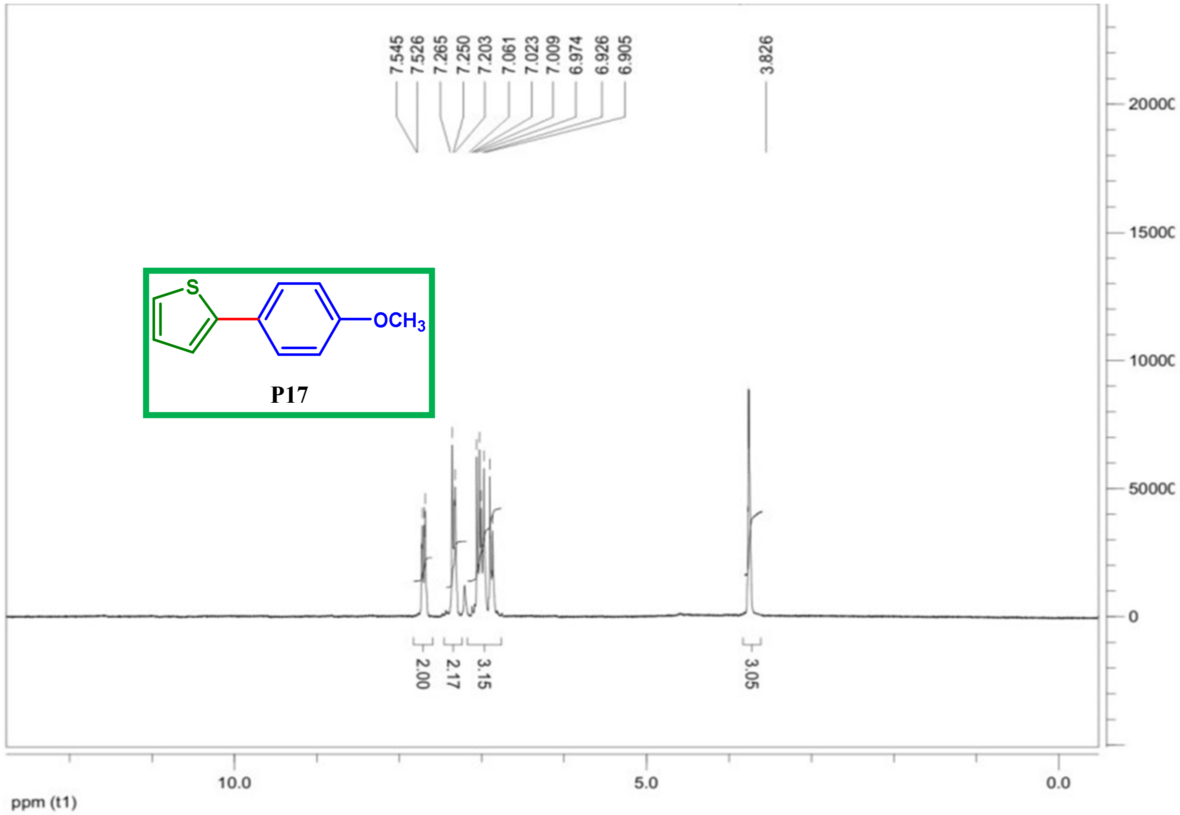
7.545
7.526
7.265
7.250
7.203
7.061
7.023
7.009
6.974
6.926
6.905
3.826
S
OCH3
P17
2.00
2.17
3.15
3.05
20000
15000
10000
5000
0
10.0
5.0
0.0
ppm (t1)

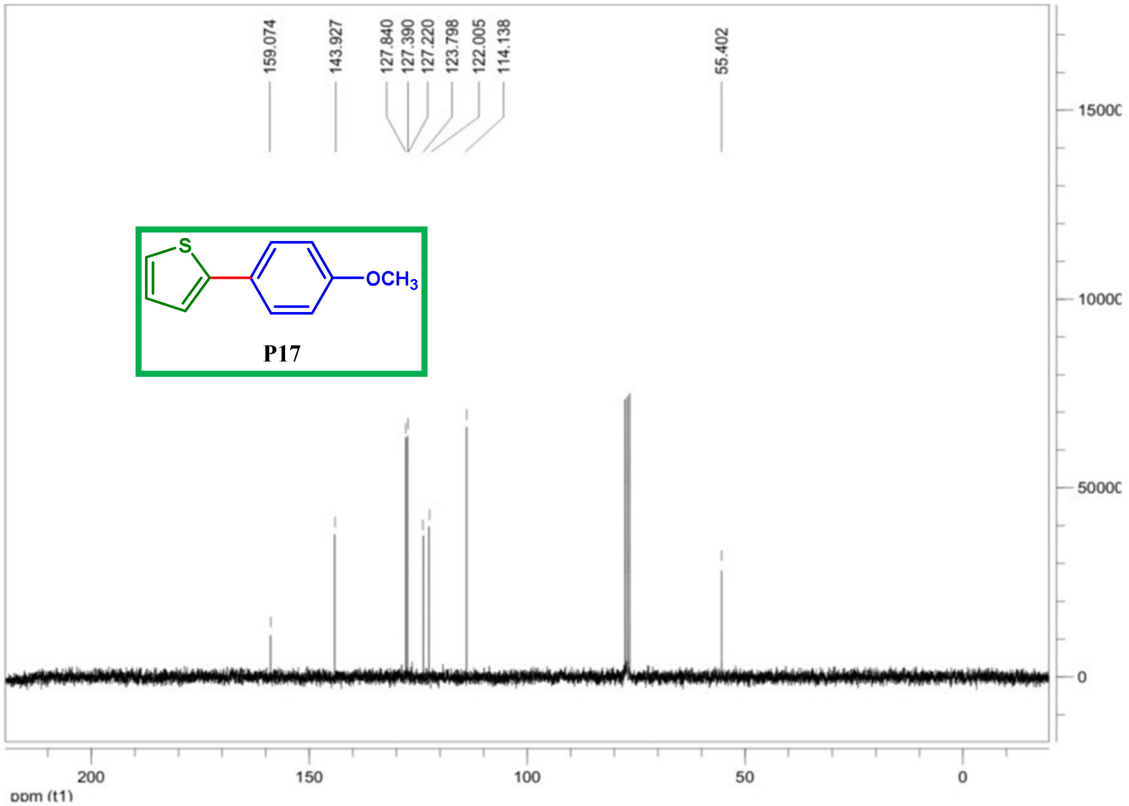
159.074
143.927
127.840
127.390
127.220
123.798
122.005
114.138
55.402
S
OCH3
P17
15000
10000
5000
0
200
150
100
50
0
ppm (t1)

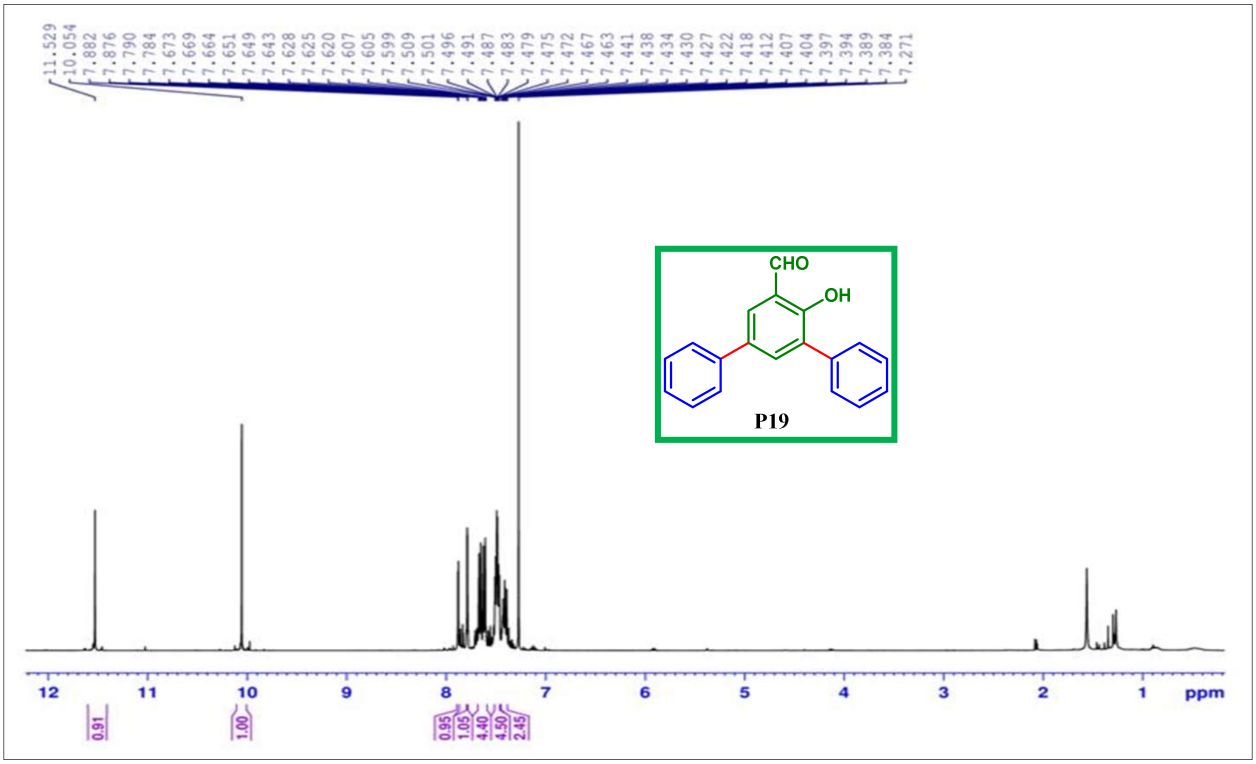
11.529
10.054
7.882
7.876
7.790
7.784
7.673
7.669
7.664
7.651
7.649
7.643
7.628
7.625
7.620
7.607
7.605
7.599
7.509
7.501
7.496
7.491
7.487
7.483
7.479
7.475
7.472
7.467
7.463
7.441
7.438
7.434
7.430
7.427
7.422
7.418
7.412
7.407
7.404
7.397
7.394
7.389
7.384
7.271
CHO
OH
P19
12
11
10
9
8
7
6
5
4
3
2
1
ppm
0.91
1.00
0.95
1.05
4.40
4.50
2.45

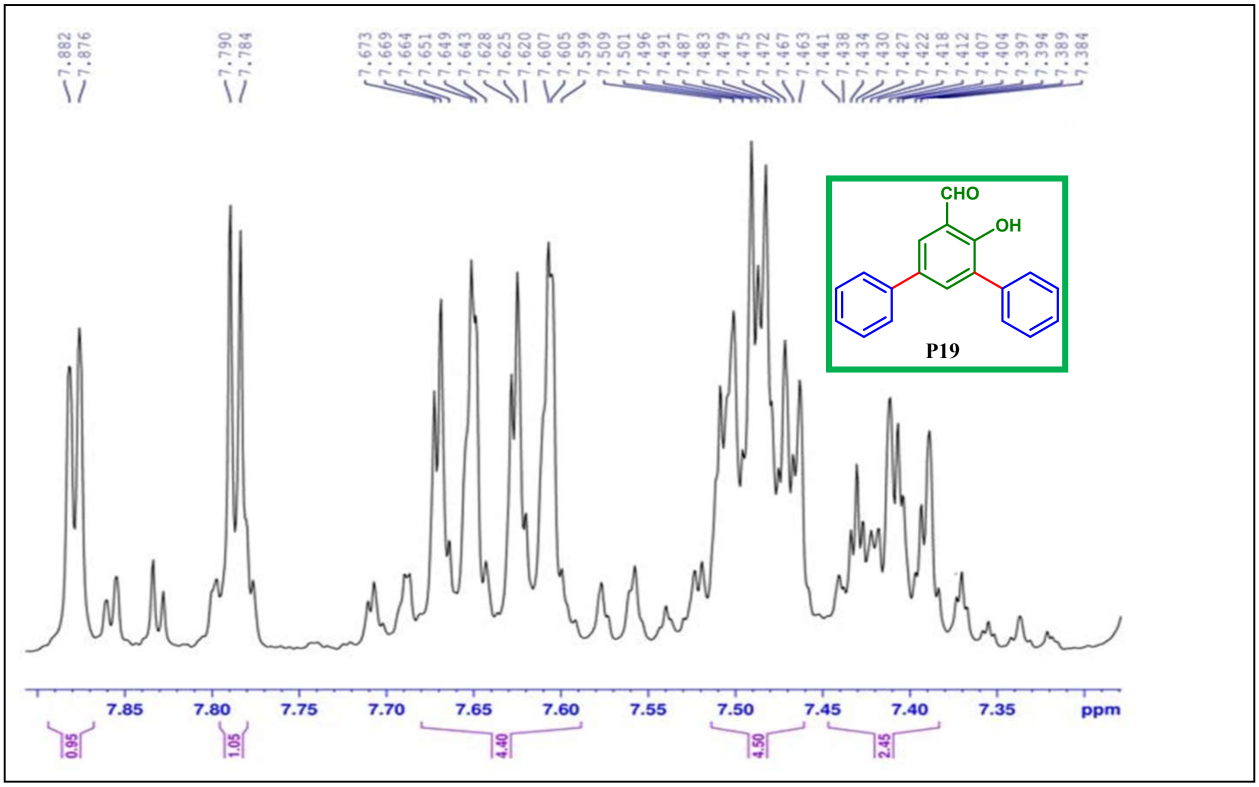
7.882
7.876
7.790
7.784
7.673
7.669
7.664
7.651
7.649
7.643
7.628
7.625
7.620
7.607
7.605
7.599
7.509
7.501
7.496
7.491
7.487
7.483
7.479
7.475
7.472
7.467
7.463
7.441
7.438
7.434
7.430
7.427
7.422
7.418
7.412
7.407
7.404
7.397
7.394
7.389
7.384
CHO
OH
P19
7.85
7.80
7.75
7.70
7.65
7.60
7.55
7.50
7.45
7.40
7.35
ppm
0.95
1.05
4.40
4.50
2.45

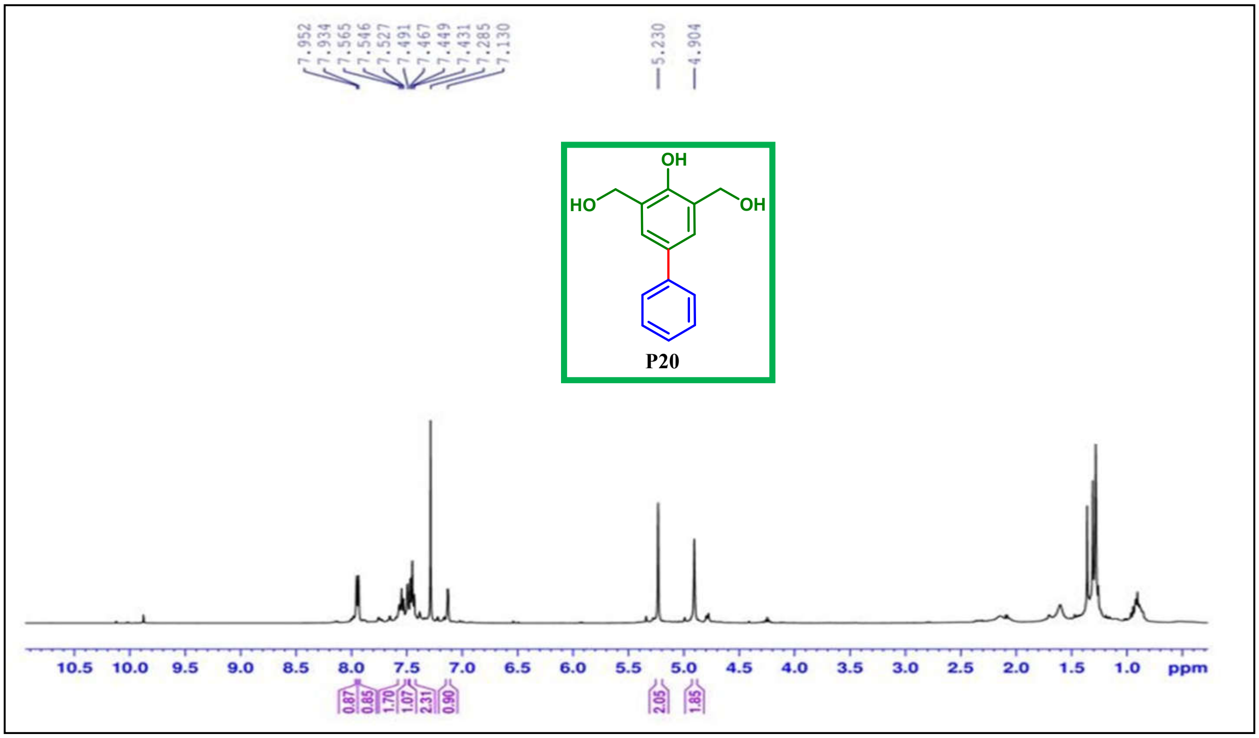
7.952
7.934
7.565
7.546
7.527
7.491
7.467
7.449
7.431
7.285
7.130
5.230
4.904
OH
HO
OH
P20
10.5
10.0
9.5
9.0
8.5
8.0
7.5
7.0
6.5
6.0
5.5
5.0
4.5
4.0
3.5
3.0
2.5
2.0
1.5
1.0
ppm
0.87
0.85
1.70
1.07
2.31
0.90
2.05
1.85

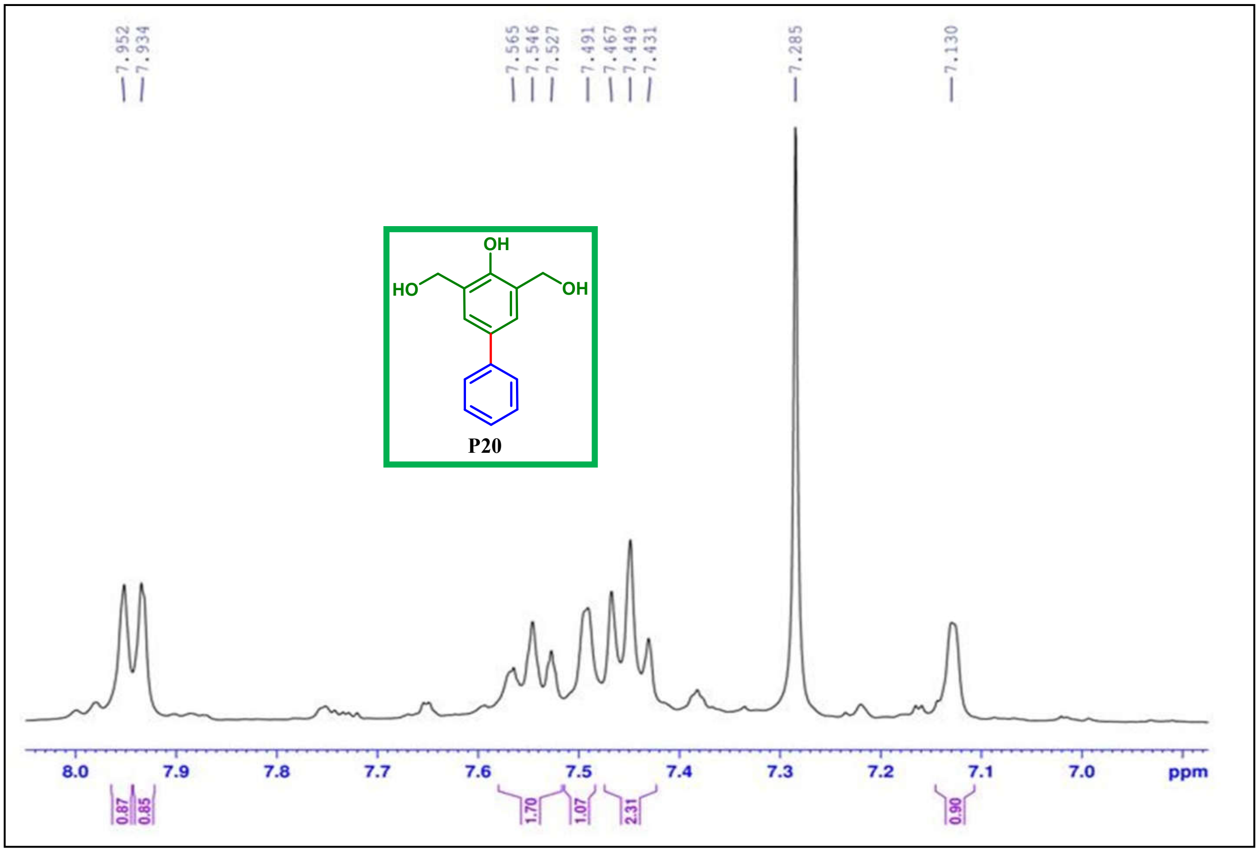
7.952
7.934
7.565
7.546
7.527
7.491
7.467
7.449
7.431
7.285
7.130
OH
HO
OH
P20
8.0
7.9
7.8
7.7
7.6
7.5
7.4
7.3
7.2
7.1
7.0
ppm
0.87
0.85
1.70
1.07
2.31
0.90

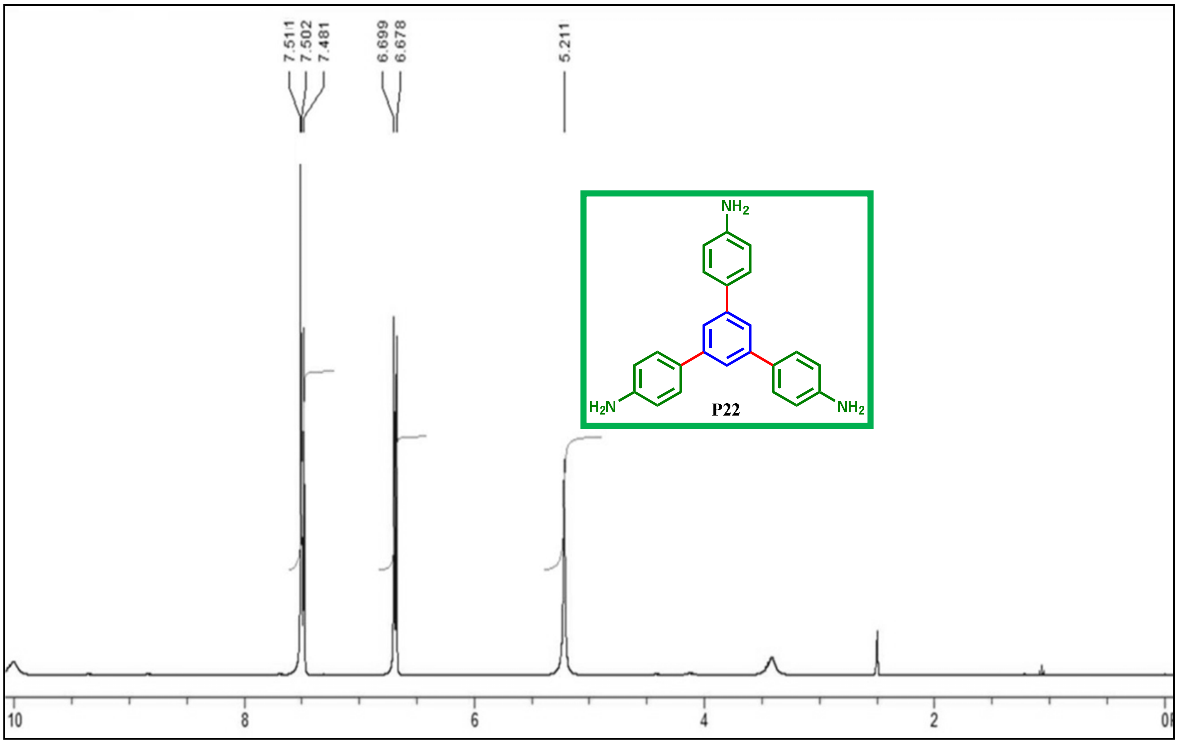

7.511
7.502
7.481
6.699
6.678
5.211
NH2
H2N
NH2
P22
10
8
6
4
2

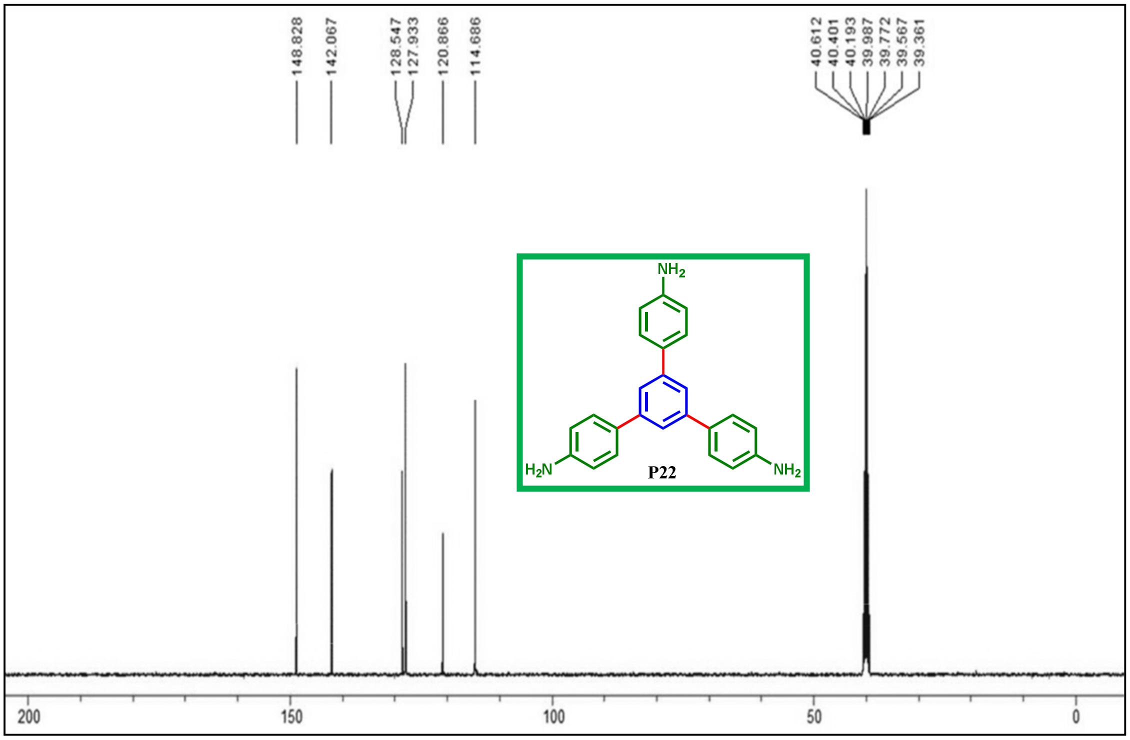

148.828
142.067
128.547
127.933
120.866
114.686
40.612
40.401
40.193
39.987
39.772
39.567
39.361
NH2
H2N
NH2
P22
200
150
100
50
0

Supporting References:

1. Tokalıoğlu Ş, Oymak T, Kartal Ş (2004) Determination of palladium in various samples by atomic absorption spectrometry after preconcentration with dimethylglyoxime on silica gel. Anal Chim Acta 511 (2):255-260. doi:https://doi.org/10.1016/j.aca.2004.02.015

2. Erinc G, Magee RJ (1964) The determination of palladium by atomic absorption spectroscopy. Anal Chim Acta 31:197-205. doi:https://doi.org/10.1016/S0003-2670(00)88809-1

3. Sarvestani M, Azadi R (2017) Palladium nanoparticles deposited on a graphene–benzimidazole support as an efficient and recyclable catalyst for aqueous-phase Suzuki–Miyaura coupling reaction. Appl Organomet Chem 31 (8):e3667. doi:doi:10.1002/aoc.3667

4. Fareghi-Alamdari R, Golestanzadeh M, Zekri N, Mavedatpoor Z (2015) Multi SO3H supported on carbon nanotubes: a practical, reusable, and regioselective catalysts for the tert-butylation of p-cresol under solvent-free conditions. J Iran Chem Soc 12 (3):537-549. doi:10.1007/s13738-014-0511-x

5. Naeimi H, Golestanzadeh M (2014) Highly sulfonated graphene and graphene oxide nanosheets as heterogeneous nanocatalysts in green synthesis of bisphenolic antioxidants under solvent free conditions. RSC Adv 4 (99):56475-56488. doi:10.1039/C4RA10177D

6. Varala R, Nuvula S, Adapa SR (2006) Molecular Iodine-Catalyzed Facile Procedure for N-Boc Protection of Amines. J Org Chem 71 (21):8283-8286. doi:10.1021/jo0612473
